# Ageing‐Dependent Thyroid Hormone Receptor α Reduction Activates IP3R1‐Meditated Ca^2+^ Transfer in MAM and Exacerbates Skeletal Muscle Atrophy in Mice

**DOI:** 10.1111/cpr.70120

**Published:** 2025-08-24

**Authors:** Runqing Shi, Yusheng Zhang, Gong Chen, Jiru Zhang, Jing Liu, Hao Zhu, Minne Sun, Yu Duan

**Affiliations:** ^1^ Department of Gerontology The First Affiliated Hospital With Nanjing Medical University Nanjing China

**Keywords:** IP3R1, MAM, mitochondrial Ca^2+^ overload, sarcopenia, senescence, thyroid hormone receptor α

## Abstract

Sarcopenia profoundly impacts the quality of life and longevity in elderly populations. Notably, alterations in thyroid hormone (TH) levels during ageing are intricately linked to the development of sarcopenia. In skeletal muscle, the primary action of TH is mediated through the thyroid hormone receptor alpha (TRα). Emerging evidence suggests that decreased TRα expression may precipitate mitochondrial dysfunction in ageing skeletal muscle tissues. Yet, the precise mechanisms and the potential causative role of TRα deficiency in sarcopenia are not fully understood. This study suggests that TRα may regulate mitochondrial calcium (Ca^2+^) transport across membranes by targeting the inositol 1,4,5‐trisphosphate receptor 1 (IP3R1), as evidenced by ChIP‐seq and RNA‐seq analyses. Experiments using naturally aged mice, skeletal muscle‐specific TRα knockout (SKT) mice, and C2C12 myoblasts were conducted to investigate this process further. Findings include increased IP3R1, mitochondria‐associated endoplasmic reticulum membranes (MAM), and mitochondrial Ca^2+^ in aged skeletal muscle. Additionally, SKT mice exhibited smaller muscle fibres, increased IP3R1 and MAM, and mitochondrial dysfunction. ChIP‐qPCR and TRα manipulation in C2C12 cells showed that TRα negatively regulates IP3R1 transcription. Moreover, TRα knockdown cells exhibited increased Ca^2+^ transfer in MAM and mitochondrial dysfunction, which was ameliorated by the IP3R1 inhibitor 2‐aminoethoxydiphenyl borate. Reintroduction of TRα improved IP3R1‐mediated mitochondrial Ca^2+^ overload in aged cells. Our findings uncover a novel mechanism by which TRα deficiency induces mitochondrial Ca^2+^ overload through IP3R1‐mediated Ca^2+^ transfer in MAM, exacerbating skeletal muscle atrophy during ageing. The TRα/IP3R1 pathway in MAM Ca^2+^ transfer presents a potential therapeutic target for sarcopenia.

## Graphical abstract

During skeletal muscle ageing, down‐regulation of TRα expression activates IP3R1 transcriptional expression, promotes MAM calcium transport, triggers mitochondrial calcium overload and apoptosis, leading to skeletal muscle atrophy. Restoring TRα expression can improve IP3R1‐mediated mitochondrial calcium overload and apoptosis in ageing skeletal muscle cells.

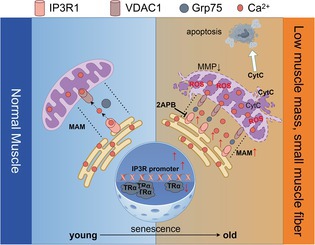

## 1 Introduction

Primary sarcopenia is recognised as a progressive, systemic skeletal muscle disorder associated with ageing, marked by reductions in muscle mass, strength, and functional capacity. This condition significantly impairs elderly mobility and increases fall risk, posing severe threats to their health and longevity [1]. Various age‐related factors such as stem cell depletion, hormonal disorders, mitochondrial dysfunction, chronic inflammation, and apoptosis contribute to sarcopenia [2], yet its pathological mechanisms remain poorly understood. Consequently, effective prevention and treatment methods are lacking.

Skeletal muscle, the body's largest organ of energy metabolism, contains a high concentration of mitochondria [3]. The balance of mitochondrial calcium (Ca^2+^) is vital for maintaining mitochondrial homeostasis [4], which is essential for normal skeletal muscle function. Insufficient mitochondrial Ca^2+^ levels inhibit energy production, impacting skeletal muscle fibre function [5]. Conversely, excessive mitochondrial Ca^2+^ can lead to overload [6], disrupting oxidative phosphorylation, enhancing cytochrome C (CytC) oxidase activity excessively, and increasing reactive oxygen species (ROS) production [7]. Excess Ca^2+^ disrupts the electrochemical gradient of the inner membrane, reducing mitochondrial membrane potential (MMP), and damages mitochondrial membrane integrity by activating phospholipases that decompose membrane phospholipids [8]. Furthermore, under conditions of Ca^2+^ overload, the mitochondrial permeability transition pore may open abnormally, releasing mitochondrial contents such as CytC into the cytoplasm and activating intrinsic apoptotic signalling pathways [9]. Increased apoptosis in skeletal muscle, which numerous studies have demonstrated in the elderly [10, 11], is closely linked to sarcopenia. In summary, mitochondrial Ca^2+^ overload leads to mitochondrial dysfunction and apoptosis, contributing to impaired skeletal muscle function and cell loss.

The maintenance of mitochondrial Ca^2+^ homeostasis is governed by a balance between Ca^2+^ uptake and efflux [12]. Primarily sourced from the endoplasmic reticulum (ER), mitochondrial Ca^2+^ is transferred mainly through the mitochondria‐associated endoplasmic reticulum membrane (MAM) [13]. As a critical interface between the ER and mitochondria, MAM regulates mitochondrial Ca^2+^ homeostasis and lipid metabolism, among other vital processes, and is intimately linked to ageing [14]. Recent studies have shown that an increase in MAM accelerates cellular senescence [15, 16]. The process of Ca^2+^ transfer in MAM starts with its release from the inositol 1,4,5‐trisphosphate receptor (IP3R) located on the ER membrane, mediated by the glucose‐regulated protein 75 (Grp75), and its subsequent entry into mitochondria through the voltage‐dependent anion channel 1 (VDAC1) on the outer mitochondrial membrane [17]. This assembly of IP3R, Grp75, and VDAC1 forms not only the structural foundation of MAM but also the principal pathway for Ca^2+^ movement. IP3R, a key regulator of Ca^2+^ release from the ER, plays a pivotal role in the formation and functionality of the IP3R/Grp75/VDAC1 complex [18]. IP3R has three isoforms: IP3R1, IP3R2 and IP3R3. Studies demonstrate that altering IP3R expression markedly affects this complex's formation [19], and IP3R2 knockout reduces MAM and improves ageing indicators in aged mice [20]. Additionally, increased IP3R1 and mitochondrial Ca^2+^ levels in aged C2C12 myotubes underscore IP3R's significant role in skeletal muscle ageing [21].

Age‐related alterations in thyroid hormones (THs) are closely associated with sarcopenia [22, 23]. THs, key regulators of skeletal muscle, exert biological effects predominantly through binding to TH receptors (TR) in muscle tissue. TR, functioning as a nuclear transcription factor, regulates gene transcription by interacting with thyroid hormone response element (TRE) sequences in these genes' promoter regions. TRα is the most prevalent type in skeletal muscle [24]. Studies have confirmed that TRα modulates the proliferation and differentiation of mouse myoblasts, impacting muscle regeneration and repair [25]. Furthermore, decreased TRα expression in aged skeletal muscle suggests its involvement in ageing‐related processes, including mitophagy, mitochondrial dynamics, and oxidative phosphorylation [26]. However, the specific mechanisms through which TRα down‐regulation leads to mitochondrial dysfunction and affects skeletal muscle during ageing remain to be elucidated.

In this study, we first integrated chromatin immunoprecipitation sequencing (ChIP‐seq) and RNA sequencing (RNA‐seq) analyses of skeletal muscle tissues from naturally ageing mice to identify IP3R1 as a potential target of TRα in skeletal muscle ageing. Based on this, we hypothesized that TRα downregulation targets IP3R1, thereby affecting MAM‐mediated mitochondrial Ca^2+^ balance and subsequently disrupting mitochondrial homeostasis in skeletal muscle cells. We further investigated how TRα downregulation disrupts mitochondrial Ca^2+^ homeostasis and contributes to sarcopenia using naturally ageing mice, skeletal muscle‐specific TRα knockout (SKT) mice, and C2C12 cells.

## 2 Materials and Methods

### 2.1 Experimental Animal

A total of 48 male C57BL/6J mice were included in this study, of which 36 were purchased from the Experimental Animal Center of Nanjing Medical University. The mice were divided into young (6 months), middle‐aged (15 months), and old (21 months) groups (*n* = 8 per group). Additionally, skeletal muscle‐specific TRα knockout mice were obtained from GemPharmatech (Nanjing, China). TRα^fl/fl^ and Myf5‐Cre mice were crossbred to produce skeletal muscle‐specific knockout mice. The TRα^fl/fl^ mice served as controls (*n* = 6), and the Myf5‐Cre mice with TRα deletion were designated as CKO mice (*n* = 6). Genomic DNA was extracted from tail samples, and PCR analysis was conducted; primer details are provided in Table S1. Phenotypes were assessed and tissues collected at 8 weeks of age. All mice were housed in groups of 3–5 per cage under specific‐pathogen‐free (SPF) conditions, maintained on a 12‐h light/dark cycle, and acclimatised under appropriate temperature and humidity conditions for 1 week prior to experiments. Food and water were provided ad libitum. Grip strength and body weight were measured every 24 h for 72 h. Subsequently, the mice were anaesthetised, and the gastrocnemius (GA) muscles of the mice were excised and weighed. The sample size for animal experiments was calculated using the “resource equation” method [27]. Mice were randomly selected from each group using a random number table for subsequent experiments, and sample tubes were digitally labelled to ensure examiner blinding. All experiments were conducted in accordance with the ethical approval of the Institutional Animal Care and Use Committee of Nanjing Medical University on June 3, 2024 (Ethical approval number: IACAC‐2406006). The anaesthetic 2,2,2‐tribromoethanol and other necessary measures were employed to minimise animal suffering during all experimental procedures.

### 2.2 Culture and Treatment of C2C12 Myoblasts

C2C12 myoblasts were purchased from the Cell Bank of the Chinese Academy of Sciences. C2C12 cells were cultured at 37°C in a 5% CO_2_ and high‐glucose DMEM environment with 10% fetal bovine serum (FBS). Ageing was induced in vitro by treating the cells with D‐galactose (D‐gal, Beyotime, China) for 24 h. TRα‐targeting siRNAs and overexpression plasmids (GenePharma, Shanghai, China) were introduced into C2C12 cells using Lipofectamine 3000 (Invitrogen, USA), and replaced with high‐glucose DMEM with 10% FBS the next day. For inhibitor experiments, 2‐aminoethoxydiphenyl borate (2APB, 50 μM, Selleck, USA, S6657) was administered for 12 h.

### 2.3 Grip Strength Test

Each mouse was instructed to grasp the dynamometer bar with its forepaw and was gently pulled backward by its tail. Built‐in sensors automatically recorded peak tension until the mouse released the bar. Each measurement was repeated three times, and the average of these three measurements was recorded [28].

### 2.4 Histology

The GA tissues preserved in 4% paraformaldehyde were removed and embedded in paraffin. The embedded GA tissues were cut into 3 μm thick sections for haematoxylin–eosin (HE) staining. After completion of staining, each section was scanned using a panoramic slice scanner (3DHISTECH, Hungary). For each slice, we measured no less than 100 muscle fibre cross‐sectional areas.

### 2.5 ROS Staining

By staining GA muscle with dihydroethidium (Servicebio, China), we were able to measure ROS levels. Pannoramic slice scanning images were taken by 3DHISTECH. Three separate muscle samples were examined for every group. Using Image J software (NIH, Bethesda, MD, USA), the mean fluorescence intensity (MFI) was assessed.

### 2.6 Transmission Electron Microscopy

Following three washes with phosphate‐buffered saline, the GA muscle and treated C2C12 cells were fixed with 2.5% glutaraldehyde in sodium phosphate buffer. The samples were subsequently embedded in Araldite after being post‐fixed in 1% osmium tetroxide and dehydrated via a graduated series of alcohols. Parts that were extremely thin were sliced and stained twice: once with uranyl acetate and again with lead citrate. Using a transmission electron microscope (HT7800, Hitachi, Japan), MAMs were analysed. By taking readings at three different spots between the membranes of the two organelles at random, we were able to calculate the average thickness of each MAM. As a fraction of the mitochondrial perimeter surrounded by MAMs, MAM coverage was computed [29].

### 2.7 Real‐Time qPCR

We isolated total RNA and synthesised cDNA from GA muscle tissue or C2C12 cells using TRIzol technology and PrimeScript RT Master Mix kit (Vazyme Biotech, China). Subsequently, RT‐qPCR was performed on a LightCycler480 II system (Roche, USA) using the SYBR‐Green kit (Vazyme Biotech, China). Table S2 contains the primer sequences. The 2^ΔΔ−CT^ was used to measure relative mRNA levels.

### 2.8 Western Blot

Protein samples were transferred to PVDF membranes using SDS–PAGE and, after blocking with 5% non‐fat milk, the membranes were placed in the corresponding primary antibodies and incubated overnight at 4°C. Detailed antibody information is provided in Table S3. The following day, membranes were incubated with the corresponding secondary antibodies (1:5000, SA00001‐1, SA00001‐2, Proteintech) for 2 h at room temperature and subsequently developed using the Tanon luminescence imaging system (China). The band density was calculated using Image J.

### 2.9 RNA‐Seq Analysis

RNA‐seq data of GA tissue from 6‐month‐old and 21‐month‐old mice that we have published and made publicly available were used for analysis (GEO, GSE281839) [30]. Gene sets from the Molecular Signatures Database and GSEA were used for functional annotation of gene sets.

### 2.10 ChIP‐Seq Analysis and ChIP‐qPCR

ChIP‐seq data of GA tissue from 6, 15, and 21‐month‐old mice that we have published and made publicly available was used for analysis (GEO, GSE281837) [30]. Gene sets from the Molecular Signatures Database and functional annotations were applied to gene sets using KEGG pathways and GO enrichment.

For ChIP‐qPCR, C2C12 cells were cross‐linked with 1% formaldehyde, resuspended in lysis buffer, and then disrupted by sonication to obtain DNA fragments ranging from 200 to 500 bp in length. 10 μg of anti‐TRα antibody (ab53729, Abcam) was incubated with 100 μL of chromatin overnight at 4°C. The next day, 30 μL protein magnetic beads were added, washed, and dissolved in elution buffer. The cells were then treated with 8 μg/mL RNase A and incubated for 6 h at 65°C. Finally, proteinase K was used to uncross‐link at 45°C. The samples were analysed by ChIP‐qPCR using the primer sequences in Table S4.

### 2.11 Immunofluorescence Staining

Frozen sections of mouse GA muscle tissue or treated C2C12 cells, fixed with 4% paraformaldehyde, were subjected to IF staining. Sections (3 μm thick) from three mice per group were prepared. The sections were prepared for primary antibody incubation at 4°C overnight after permeabilisation with 0.2% Triton X‐100 in PBS and blocking with goat serum. The antibodies used were translocator of the outer mitochondrial membrane 20 (TOM20) (1:200, 11802‐1‐AP, Proteintech) and cytochrome C (CytC) (1:200, 66264‐1‐Ig, Proteintech). Two hours of room temperature application of secondary antibodies tagged with fluorescein was performed the following day. The nuclei were stained with 4,6‐diamidino‐2‐phenylindole (DAPI, #4083, CST).

### 2.12 Mitochondrial Fluorescent Staining

To detect MMP, ROS, Ca^2+^, and mitochondria, respectively, live C2C12 cells in confocal dishes were incubated with JC‐1 (Beyotime, China), MitoSOX Red (5 μM, Thermo Fisher Scientific, USA, M36008), Rhod 2 AM (Maokang Biotech, China), and MitoTracker Green (200 nM, Thermo Fisher Scientific, USA, M7514). As needed, Hoechst stain (KeyGEN, China) was applied to the nuclei. The mitochondria and ER were both labelled with Mito‐Tracker and ER‐Tracker (KeyGEN, China, KGMP016‐1) in MAM. Stellaris STED (LEICA, Germany) was used to acquire the images. In order to analyse MFI and MGV, Image J was used. We computed the Pearson correlation coefficient to find out how well the fluorophores were colocalised.

### 2.13 Flow Cytometry

Rhod 2 AM, MitoSOX Red, and JC‐1 were used to measure mitochondrial ROS and mitochondrial Ca^2+^ levels, respectively, in live C2C12 cells. The fluorescence intensity of single cells suspended in solution was measured using FC. The MFI was analysed using the FlowJo programme.

### 2.14 Co‐Immunoprecipitation

Lysis buffer (Beyotime, China) was used to lyse GA muscle tissues or treated C2C12 cells for co‐immunoprecipitation (CoIP). Before being incubated with Protein A/G Magnetic Beads (HY‐K0202, MCE, China), proteins were incubated with an IP3R antibody (1 μg per 1 mg of total protein, 19962‐1‐AP, Proteintech) overnight at 4°C. Mix 500 μL of the antibody–antigen sample with 25 μL of magnetic bead slurry, and then incubate at 4°C with rotation for 4 h. Before being analysed by immunoblotting, the beads were boiled in 40 μL of sample loading buffer (P0015B, Beyotime, China).

### 2.15 Terminal Deoxynucleotidyl Transferase dUTP Nick End Labeling

Sectioned tissues or treated C2C12 cells were subjected to the TUNEL assay after being permeabilised with 0.2% Triton X‐100 in PBS. Following the manufacturer's instructions, apoptotic cells were detected using the TUNEL cell apoptosis detection kit (KeyGEN, China, KGA1407‐10) by integrating fluorescein‐12‐dUTP at the 3′ OH ends of damaged DNA using recombinant Tdt enzyme. The nuclei were stained with DAPI after the reaction, and the slides were covered after applying an anti‐fluorescence quenching agent.

### 2.16 Statistical Analysis

Data analysis was conducted using Prism 9 software (GraphPad Software, San Diego, CA). For two‐group comparisons, the Student's *t*‐test was utilised, while one‐way ANOVA was applied for analyses involving multiple groups. Results are presented as means ± SD derived from three separate experiments. All experiments were repeated at least three times. Significance was denoted as follows: *p < 0.05, **p < 0.01, ***p < 0.001, and ****p < 0.0001. Results not reaching statistical significance were noted as (ns).

## 3 Results

### 3.1 IP3R1 is a Potential Target for TRα to Regulate Mitochondrial Ca^2+^ Influx in Ageing Skeletal Muscle

**FIGURE 1:**
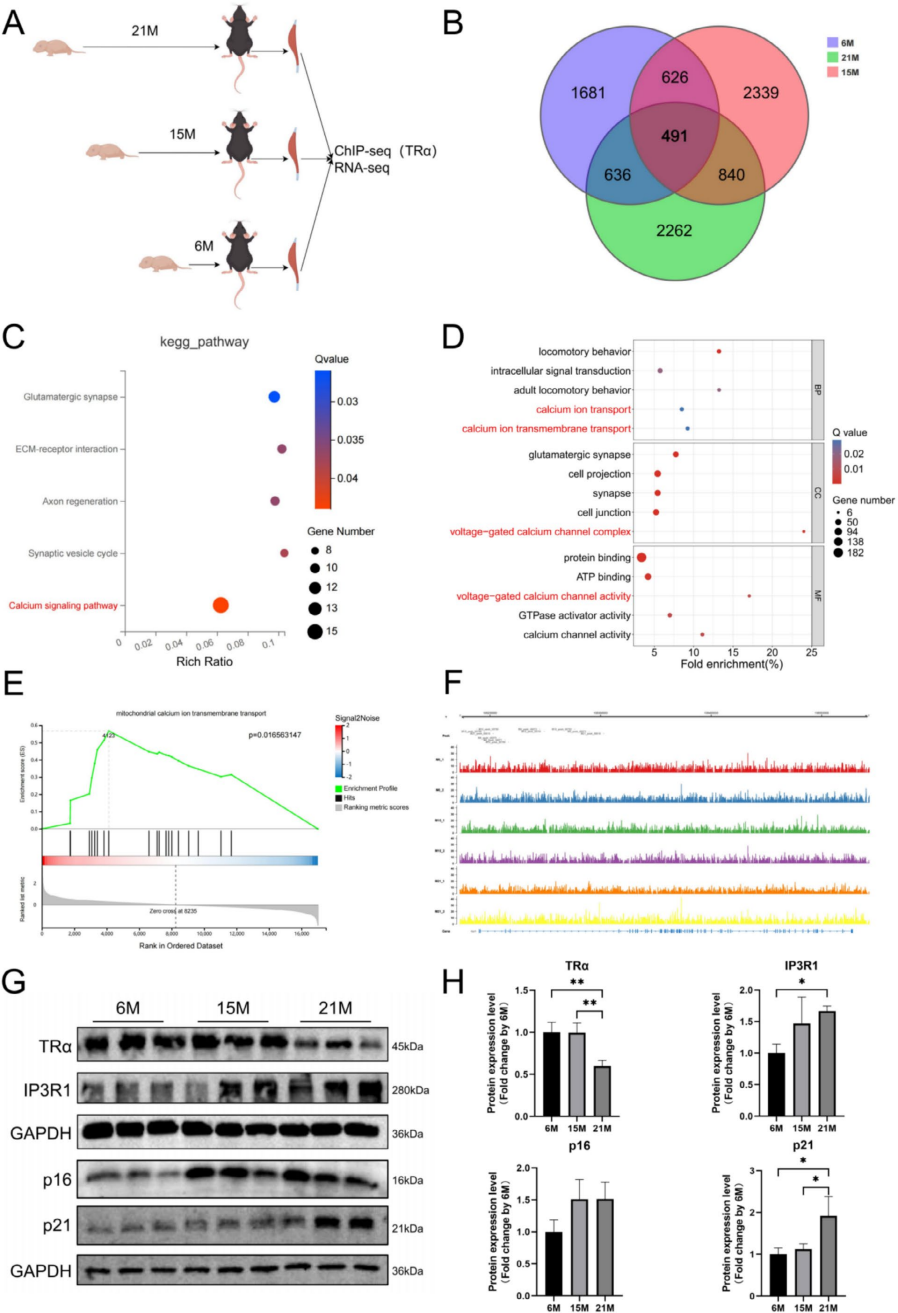
Inositol 1,4,5‐trisphosphate receptor 1 (IP3R1) is a potential target for thyroid hormone receptor α (TRα) to regulate mitochondrial Ca^2+^ influx in ageing skeletal muscle. (A) Flow chart of aged mouse model construction. (B) The Venn diagram shows the intersection of TRα target genes in gastrocnemius (GA) muscle of different ages mice identified by chromatin immunoprecipitation sequencing (ChIP‐seq). Blue represents 6 months old, red represents 15 months old, and green represents 21 months old. (C) Bubble chart of KEGG pathway enrichment for 491 common target genes. (D) Bubble chart of GO enrichment for 491 common target genes. (E) GSEA on the GO of “mitochondrial calcium transmembrane transport” is shown. (F) Integrative genomics viewer showing the recruitment of TRα to the IP3R1 promoter region in the ChIP‐seq datasets. (G, H) TRα, IP3R1, p16, and p21 protein expressions measured using WB in GA muscle of mice at different age with corresponding statistics (*n* = 3). One‐way ANOVA; *p < 0.05, and **p < 0.01.

ChIP‐seq of TRα in the GA muscle of young, middle‐aged, and old mice (Figure 1A) identified 491 stable target genes of TRα during ageing (Figure 1B). KEGG pathways (Figure 1C) and GO (Figure 1D) analyses highlighted TRα's involvement in Ca^2+^ signalling pathways, Ca^2+^ channel complexes, and Ca^2+^ transmembrane transport during ageing. Analysis of RNA‐seq data through GSEA revealed that only the mitochondrial Ca^2+^ transmembrane transport pathway was significantly upregulated in old mice (Figure 1E). Among the 491 target genes, IP3R1 was found to be closely related to mitochondrial Ca^2+^ transmembrane transport. ChIP‐seq analysis demonstrated that TRα could bind to the TSS of IP3R1 across all age groups (Figure 1F), suggesting IP3R1 as a potential target gene of TRα for regulating mitochondrial Ca^2+^ transmembrane transport in aged skeletal muscle.

With increased expression of ageing markers p16 and p21, elevated IP3R1 protein (Figure 1G,H) and mRNA levels were detected in aged skeletal muscle (Figure S1A), suggesting activation of mitochondrial Ca^2+^ influx as the primary mechanism for enhanced mitochondrial Ca^2+^ transmembrane transport. Correspondingly, TRα expression decreased in aged skeletal muscle (Figure 1G,H), aligning with findings from our previous study [26]. These results indicate that TRα down‐regulation may target IP3R1 to activate mitochondrial Ca^2+^ influx in ageing skeletal muscle.

### 3.2 IP3R1‐Mediated MAM Formation and Mitochondrial Ca^2+^ Overload Are Increased in Ageing Skeletal Muscle

**FIGURE 2:**
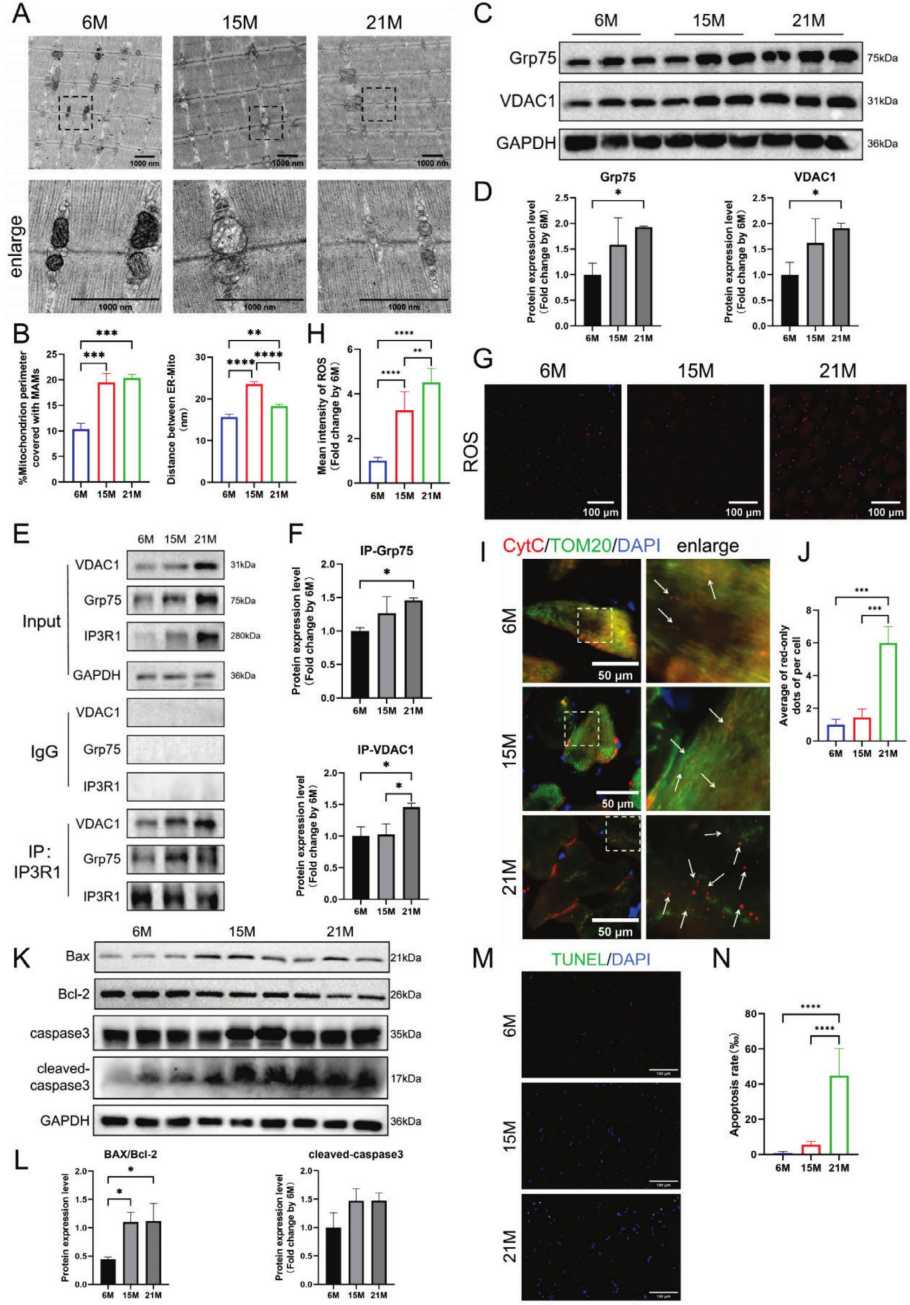
IP3R1‐mediated mitochondria‐associated endoplasmic reticulum membrane (MAM) formation and mitochondrial Ca^2+^ overload are increased in ageing skeletal muscle. (A) Representative TEM images from GA muscle used to analyse MAMs (scale bar: 1000 nm). (B) Statistics of MAM coverage and thickness in GA muscle of mice of different ages (*n* = 3). (C, D) Glucose‐regulated protein 75 (Grp75) and voltage‐dependent anion channel 1 (VDAC1) protein expressions measured using WB in GA muscle of mice at different age with corresponding statistics (*n* = 3). (E, F) CO‐IP analysis of IP3R1 interacting with Grp75, VDAC1 and GAPDH in GA muscle of mice at different age with corresponding statistics (*n* = 3). (G, H) Representative ROS fluorescence staining of GA muscles and quantification (scale bar: 100 μm, *n* = 3). (I) Representative images of translocator of the outer mitochondrial membrane 20 (TOM20) (green) co‐stained with cytochrome C (CytC) (red) in GA muscle of mice at different ages, and arrows indicate spots of CytC red fluorescence that do not colocalize with TOM20 (scale bar: 50 μm). (J) The number of CytC red fluorescent spots in each cell of GA muscle of mice at different ages that did not co‐localise with TOM20 was counted (*n* = 3). (K, L) BAX, Bcl‐2, and cleaved‐caspase3 protein expressions measured using WB in GA muscle of mice at different age with corresponding statistics (*n* = 3). (M, N) TUNEL staining was conducted to explore apoptosis in GA muscle of mice at different age and the positive cells ratio was calculated (scale bar: 100 μm, *n* = 3). One‐way ANOVA; *p < 0.05, **p < 0.01, ***p < 0.001 and ****p < 0.0001.

IP3R1 interacts with Grp75 and VDAC1 to facilitate MAM Ca^2+^ flow, regulating mitochondrial Ca^2+^ influx. GSEA indicated that MAM was upregulated in aged GA muscle (Figure S1B), a finding confirmed by TEM, which showed significantly increased MAM coverage in old mice. However, MAM thickness initially increased and then decreased in aged GA muscle (Figure 2A,B). The mRNA levels of IP3R1, Grp75, and VDAC1 were significantly elevated in aged GA muscle (Figure S1A,C,D), along with their corresponding protein levels (Figure 2C,D). CoIP results indicated increased interactions of IP3R1 to Grp75 and VDAC1 in old GA muscle compared to young mice (Figure 2E,F), suggesting that upregulation of IP3R1 contributes to abnormal increases in MAM in ageing GA muscle.

Increased mitochondrial Ca^2+^ influx led to mitochondrial Ca^2+^ overload, causing various mitochondrial dysfunctions. ROS staining of frozen sections showed that ROS MFI in GA muscle increased with age, peaking in old mice (Figure 2G,H). Confocal microscopy showed that, compared to young and middle‐aged mice, aged GA muscle exhibited a significantly higher number of CytC red fluorescent spots not co‐localised with TOM20, indicating marked mitochondrial disorganisation in aged skeletal muscle (Figure 2I,J). Additionally, GSEA showed significantly increased positive regulation of the intrinsic apoptotic signalling pathway in old mice's GA muscle (Figure S1E). Protein levels of apoptotic markers BAX/Bcl‐2 and cleaved‐caspase3 also increased with ageing (Figure 2K,L). The TUNEL assay results further indicated a significant increase in the number of positive green spots in the GA muscle of middle‐aged and old mice (Figure 2M,N), confirming that mitochondrial Ca^2+^ overload occurs in aged skeletal muscle.

### 3.3 IP3R1 Up‐Regulation Induces MAM‐Mediated Mitochondrial Ca^2+^ Overload in Aged Myoblasts

A senescent C2C12 cell model was established using D‐Gal. Upon exposure to D‐Gal concentrations of 10, 20, 40, and 60 mg/mL, significant positive β‐galactosidase staining was observed, with intensity increasing with concentration (Figure S2A,B). CCK8 assays showed a significant decline in myoblast survival rate with increasing D‐Gal concentrations, reaching 50% at 40 mg/mL (Figure S2C). The protein levels of senescence markers p16 and p21 significantly increased at 40 mg/mL compared to control (Figure S2D,E). Based on these findings, 40 mg/mL was chosen as the D‐Gal concentration for inducing senescence in C2C12 cells.

**FIGURE 3:**
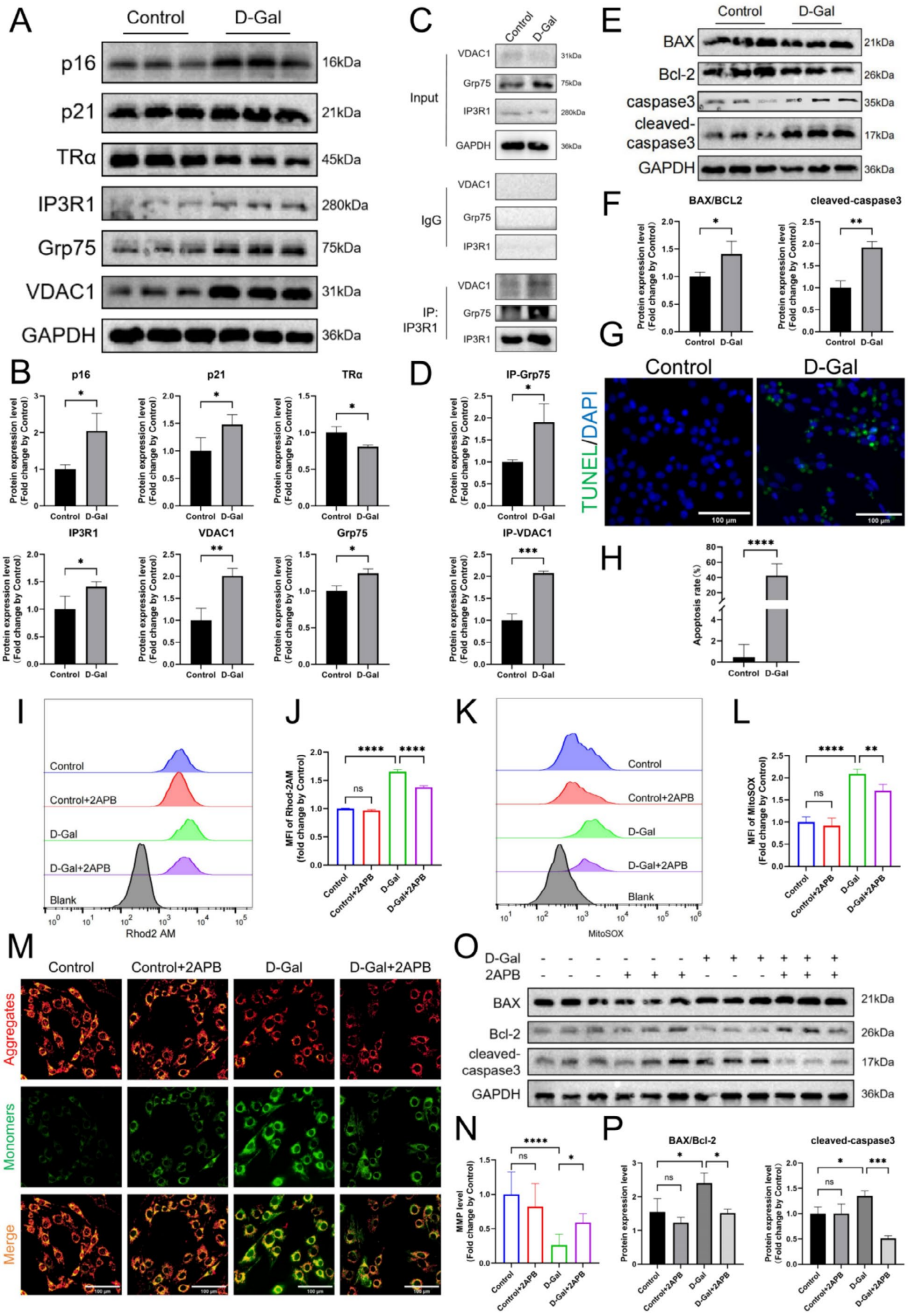
IP3R1 up‐regulation induces MAM‐mediated mitochondrial Ca^2+^ overload in aged myoblasts. (A, B) p16, p21, TRα, IP3R1, Grp75, VDAC1 and GAPDH protein expressions measured using WB in C2C12 cells with corresponding statistics (*n* = 3). (C, D) CO‐IP analysis of IP3R1 interacting with Grp75, VDAC1 and GAPDH in C2C12 cells with corresponding statistics (*n* = 3). (E, F) BAX, Bcl‐2 and cleaved‐caspase3 protein expressions measured using WB in C2C12 cells with corresponding statistics (*n* = 3). (G, H) TUNEL staining was conducted to explore apoptosis in C2C12 cells and the positive cells ratio was calculated (scale bar: 100 μm, *n* = 3). (I, J) Flow cytometry results showing the fluorescence intensity of Rhod2 AM (*n* = 6). (K, L) Flow cytometry results showing the fluorescence intensity of MitoSOX (*n* = 6). (M, N) Results of JC‐1 staining and corresponding red‐green fluorescence ratio analysis (scale bar: 100 μm, *n* = 3). (O, P) BAX, Bcl‐2 and cleaved‐caspase3 protein expressions measured using WB in different group with corresponding statistics (*n* = 3). Unpaired Student's *t*‐tests for two groups and one‐way ANOVA for multiple groups; *p < 0.05, **p < 0.01, ***p < 0.001 and ****p < 0.0001.

In senescent cells, the mRNA levels of p21 and p53 were markedly elevated compared to control cells, and p16 mRNA was not detected due to minimal expression levels. The expression of mRNA for IP3R1, Grp75, and VDAC1 also saw significant increases (Figure S2F). At the protein level, alongside notable increases in p16 and p21, TRα expression was significantly decreased, whereas IP3R1, Grp75, and VDAC1 were significantly up‐regulated (Figure 3A,B). CoIP results showed enhanced interaction of IP3R1 to Grp75 and VDAC1 in senescent cells (Figure 3C,D). Furthermore, D‐Gal stimulation significantly elevated the expression levels of apoptotic markers BAX/Bcl‐2 and cleaved caspase‐3 (Figure 3E,F). TUNEL assay results also showed a marked increase in apoptosis in aged cells (Figure 3G,H). These findings suggest that D‐Gal‐induced alterations in C2C12 cells resemble those observed in the skeletal muscle of aged mice.

Subsequent experiments using the IP3R inhibitor 2APB explored the role of IP3R1 in cellular senescence. Mitochondrial calcium, as indicated by Rhod2 AM staining, and FC demonstrated a significant rise in the mean MFI of Rhod2 AM in the D‐Gal treated group, whereas a reduction was observed in the senescence group treated with 2APB (Figure 3I,J). Additionally, MitoSOX levels were elevated in the D‐Gal group and reduced significantly in the 2APB‐treated senescence group (Figure 3K,L). The JC‐1 red‐green fluorescence ratio significantly declined in senescent cells, a change that 2APB treatment reversed (Figure 3M,N). WB showed that 2APB significantly attenuated the increased levels of BAX/Bcl‐2 and cleaved‐caspase3 in senescent cells (Figure 3O,P). These results suggest that IP3R1 up‐regulation induces mitochondrial Ca^2+^ overload in senescent myoblasts.

### 3.4 TRα Knockout in Skeletal Muscle of Mice Leads to IP3R1‐Mediated MAM Formation, Mitochondrial Dysfunction, and Skeletal Muscle Atrophy

**FIGURE 4:**
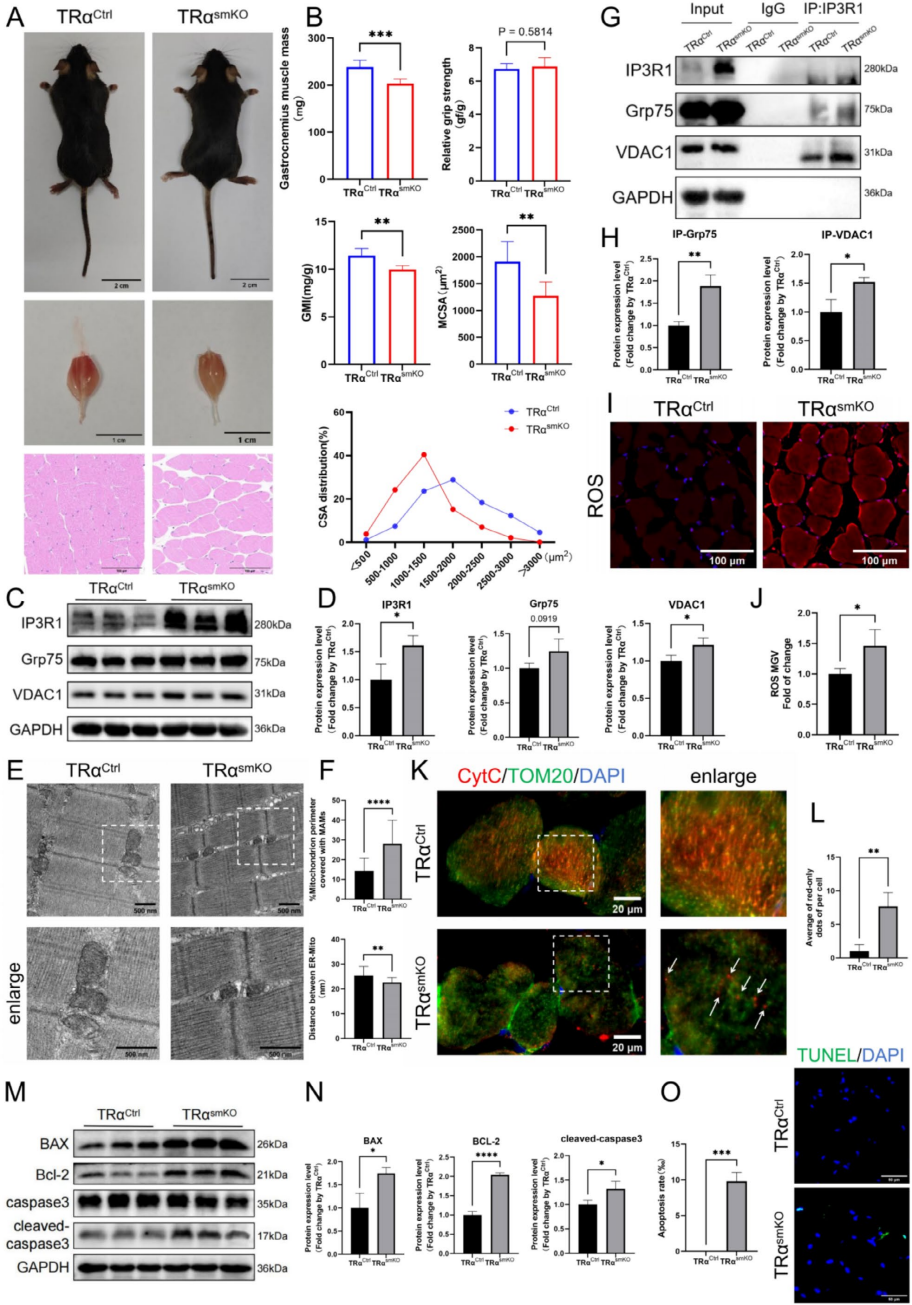
TRα knockout in skeletal muscle of mice leads to IP3R1‐mediated MAM formation, mitochondrial dysfunction, and skeletal muscle atrophy. (A) Photographs of body (scale bar: 2 cm) and GA muscle (scale bar: 1 cm) in control and skeletal muscle‐specific knockout mice of TRα (SKT); H&E‐stained cross sections in GA muscle of control and SKT mice (scale bar: 1000 nm). (B) GA muscle mass, relative grip strength, GA muscle index (GMI), mean cross‐sectional area (CSA), and distribution of CSA of GA muscle in mice (*n* = 6). (C, D) IP3R1, Grp75, VDAC1 and GAPDH protein expressions measured using WB in GA muscle of control and SKT mice with corresponding statistics (*n* = 3). (E) Representative TEM images from GA muscle used to analyse MAMs (scale bar: 500 nm). (F) Effect of TRα knockout on MAM coverage and thickness in GA muscle (*n* = 3). (G, H) CO‐IP analysis of IP3R1 interacting with Grp75, VDAC1 and GAPDH in GA muscle with corresponding statistics (*n* = 3). (I, J) Representative ROS fluorescence staining of GA muscles and quantification (scale bar: 100 μm, *n* = 3). (K) Representative images of TOM20 co‐stained with CytC in GA muscle of mice, and arrows indicate spots of CytC red fluorescence that do not colocalize with TOM20 (scale bar: 20 μm). (L) The number of CytC red fluorescent spots in each cell of GA muscle of mice that did not co‐localise with TOM20 was counted (*n* = 3). (M, N) BAX, Bcl‐2 and cleaved‐caspase3 protein expressions measured using WB in GA muscle of mice with corresponding statistics (*n* = 3). (O) TUNEL staining was conducted to explore apoptosis in GA muscle of mice at different age and the positive cells ratio was calculated (scale bar: 100 μm, *n* = 3). Unpaired Student's *t*‐tests; *p < 0.05, **p < 0.01, ***p < 0.001 and ****p < 0.0001.

The generation of SKT mice was confirmed by tail identification and qPCR, verifying the correct genotype (Figure S3A). WB revealed a significant reduction in TRα expression in the GA muscle of these mice (Figure S3B,C). No significant differences were observed in body size, body weight (Figure S3D), grip strength (Figure S3E), or relative grip strength index between SKT mice and controls. However, the GA muscle in SKT mice was smaller, and both the muscle mass and the GA muscle index (GMI), adjusted by body weight, were significantly decreased. Additionally, HE staining indicated a significant reduction in the mean cross‐sectional area (MCSA) of GA muscle fibres and an increased proportion of small‐area muscle fibres compared to controls (Figure 4A,B). These findings suggest that deletion of TRα in skeletal muscle leads to muscle atrophy in mice.

mRNA levels of IP3R1 were significantly upregulated in the GA muscle of SKT mice relative to controls, whereas Grp75 and VDAC1 mRNA remained stable (Figure S3F). Conversely, protein concentrations of IP3R1, Grp75, and VDAC1 were significantly elevated (Figure 4C,D). TEM indicated significant increases in MAM coverage and decreases in MAM thickness in these mice (Figure 4E,F). CoIP showed stronger interactions of IP3R1 to Grp75 and VDAC1 in the knockout mice (Figure 4G,H). Elevated ROS levels were detected in the GA muscle of the knockout mice (Figure 4I,J). Co‐staining for TOM20 and CytC revealed disrupted mitochondrial architecture, indicated by a significant increase in CytC red fluorescent spots that did not colocalize with TOM20 (Figure 4K,L). Both mRNA (Figure S3H) and protein expressions of pro‐apoptotic factors BAX and cleaved‐caspase3 were significantly heightened (Figure 4M,N), and TUNEL showed increased apoptosis rates in the knockout mice (Figure 4O). However, despite significant rises in both mRNA and protein levels of the anti‐apoptotic factor Bcl‐2, the BAX/Bcl‐2 remained unchanged (Figure S3G). Additionally, protein levels of p16, p21, and p‐Akt/Akt were significantly elevated in the GA muscle of SKT mice compared to controls, while p53 and PI3K levels remained unchanged (Figure S3I,J). Taken together, these results show that SKT mice exhibit abnormally increased IP3R1 expression, enhanced interaction of IP3R1 with Grp75/VDAC1, increased number of MAM, decreased MAM thickness, and mitochondrial dysfunction in skeletal muscle.

### 3.5 TRα Knockdown Increases IP3R1‐Mediated Ca^2+^ Transfer in MAM and Mitochondrial Ca^2+^ Overload in Myoblasts

**FIGURE 5:**
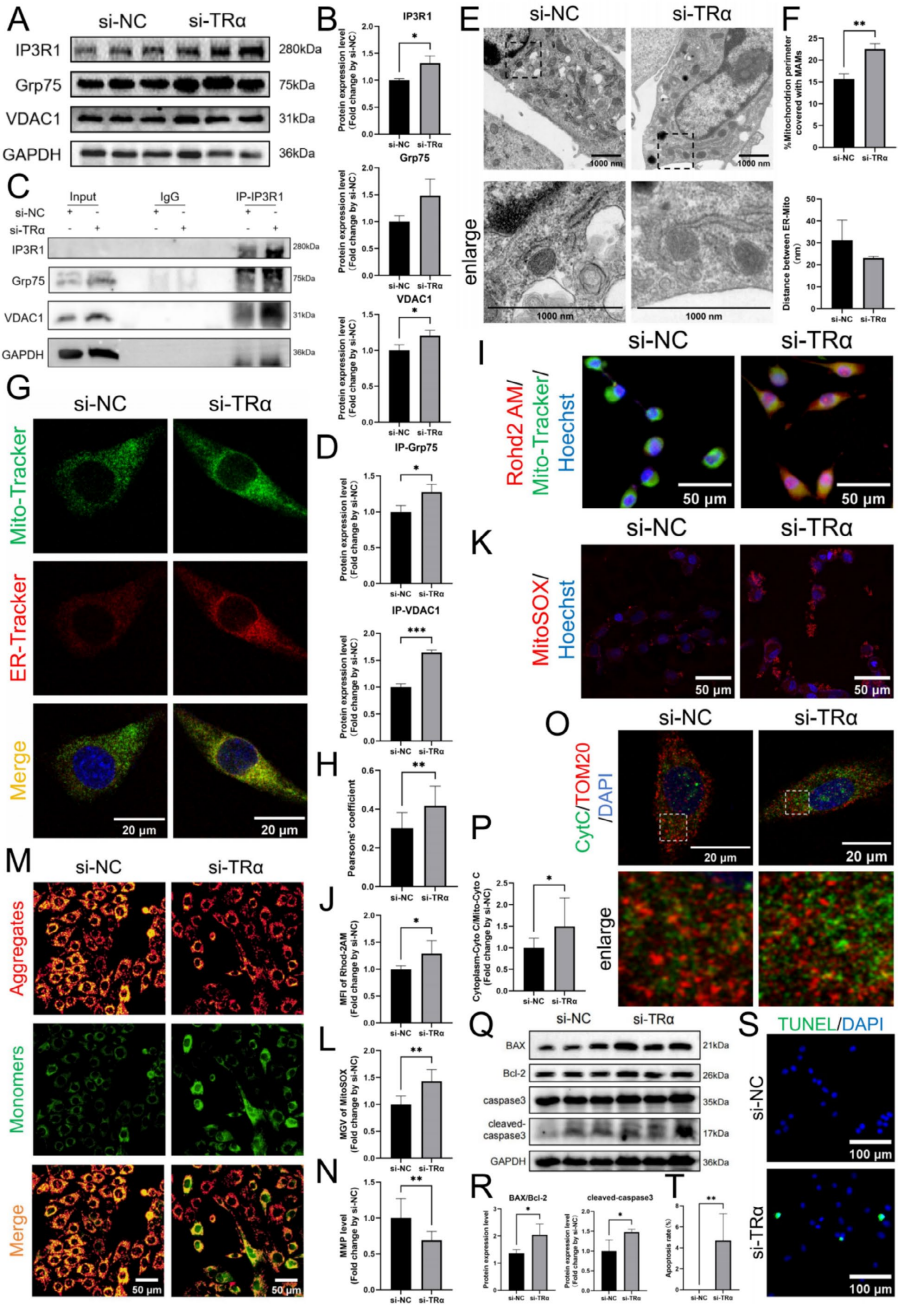
TRα knockdown increases IP3R1‐mediated Ca^2+^ transfer in MAM and mitochondrial Ca^2+^ overload in myoblasts. (A, B) IP3R1, Grp75, VDAC1 and GAPDH protein expressions measured using WB in C2C12 cells with corresponding statistics (*n* = 3). (C, D) CO‐IP analysis of IP3R1 interacting with Grp75, VDAC1 and GAPDH in C2C12 cells with corresponding statistics (*n* = 3). (E) Representative TEM images from C2C12 cells used to analyse MAMs (scale bar: 1000 nm). (F) Effect of TRα knockdown on MAM coverage and thickness in C2C12 cells (*n* = 3). (G, H) ER‐Tracker Red and Mito‐Tracker Green co‐labelling to observe MAMs, and Pearson's correlation coefficient was calculated (scale bar: 20 μm, *n* = 3). (I, J) Representative images of Mito‐Tracker green co‐stained with Rhod2 AM staining and corresponding red fluorescence intensity analysis (scale bar: 50 μm, *n* = 3). (K, L) Results of MitoSOX staining and corresponding red fluorescence intensity analysis (scale bar: 50 μm, *n* = 3). (M, N) Results of JC‐1 staining and corresponding red‐green fluorescence ratio analysis (scale bar: 50 μm, *n* = 3). (O, P) Representative images of TOM20 co‐stained with CytC in C2C12 cells, and the ratio of CytC fluorescence that did not colocalize with TOM20 was counted (scale bar: 20 μm, *n* = 3). (Q, R) BAX, Bcl‐2 and cleaved‐caspase3 protein expressions measured using WB in C2C12 cells with corresponding statistics (*n* = 3). (S, T) TUNEL staining was conducted to explore apoptosis in C2C12 cells and the positive cells ratio was calculated (scale bar: 100 μm, *n* = 3). Unpaired Student's *t*‐tests; *p < 0.05, **p < 0.01 and ***p < 0.001.

TRα knockdown in C2C12 cells was achieved using siRNA, as evidenced by significant reductions in TRα mRNA and protein levels (Figure S4A–C). In these cells, IP3R1 mRNA (Figure S4A) and protein (Figure 5A,B) levels were significantly increased, while Grp75 showed increased levels without statistical significance. VDAC1 mRNA levels remained unchanged (Figure S4A), but its protein levels were significantly increased (Figure 5A,B). CoIP demonstrated enhanced interactions of IP3R1 to Grp75 and VDAC1 following TRα knockdown (Figure 5C,D). TEM analysis revealed significantly increased MAM coverage in C2C12 cells post‐knockdown, while MAM thickness exhibited a decreasing trend, though not statistically significant (Figure 5E,F). Co‐staining with Mito‐Tracker and ER‐Tracker showed that the Pearson correlation coefficients between these markers were significantly increased after TRα knockdown (Figure 5G,H). These results indicate that TRα knockdown leads to increased IP3R1 expression, enhanced interaction between IP3R1, Grp75/VDAC1, and increased MAMs in mouse myoblasts.

Confocal microscopy revealed a significant increase in the MFI of Rhod2 AM in TRα knockdown cells (Figure 5I,J). Additionally, the MGV of MitoSOX was significantly increased following TRα knockdown (Figure 5K,L), and the red‐green fluorescence ratio of JC‐1 was significantly decreased (Figure 5M,N). Co‐staining of TOM20 and CytC showed a significant reduction in their co‐localization following TRα knockdown (Figure 5O,P). These findings suggest that TRα deletion promotes mitochondrial disorganisation. Moreover, levels of BAX/Bcl‐2 and cleaved‐caspase 3 were markedly elevated following TRα reduction (Figure 5Q,R), and TUNEL displayed prominent green fluorescence in cells lacking TRα (Figure 5S) alongside a notable rise in the apoptosis rate (Figure 5T). Additionally, protein levels of p16 and p21 were significantly elevated in TRα knockdown cells compared to controls, while levels of p53, PI3K, and p‐Akt/Akt remained unchanged (Figure S4D,E). Collectively, these results indicate that the suppression of TRα is associated with heightened mitochondrial calcium and impaired mitochondrial function, suggesting a state of mitochondrial calcium overload in mouse myoblasts.

### 3.6 TRα Knockdown Activates IP3R1 Transcriptional Expression to Induce Mitochondrial Ca^2+^ Overload in Myoblasts

**FIGURE 6:**
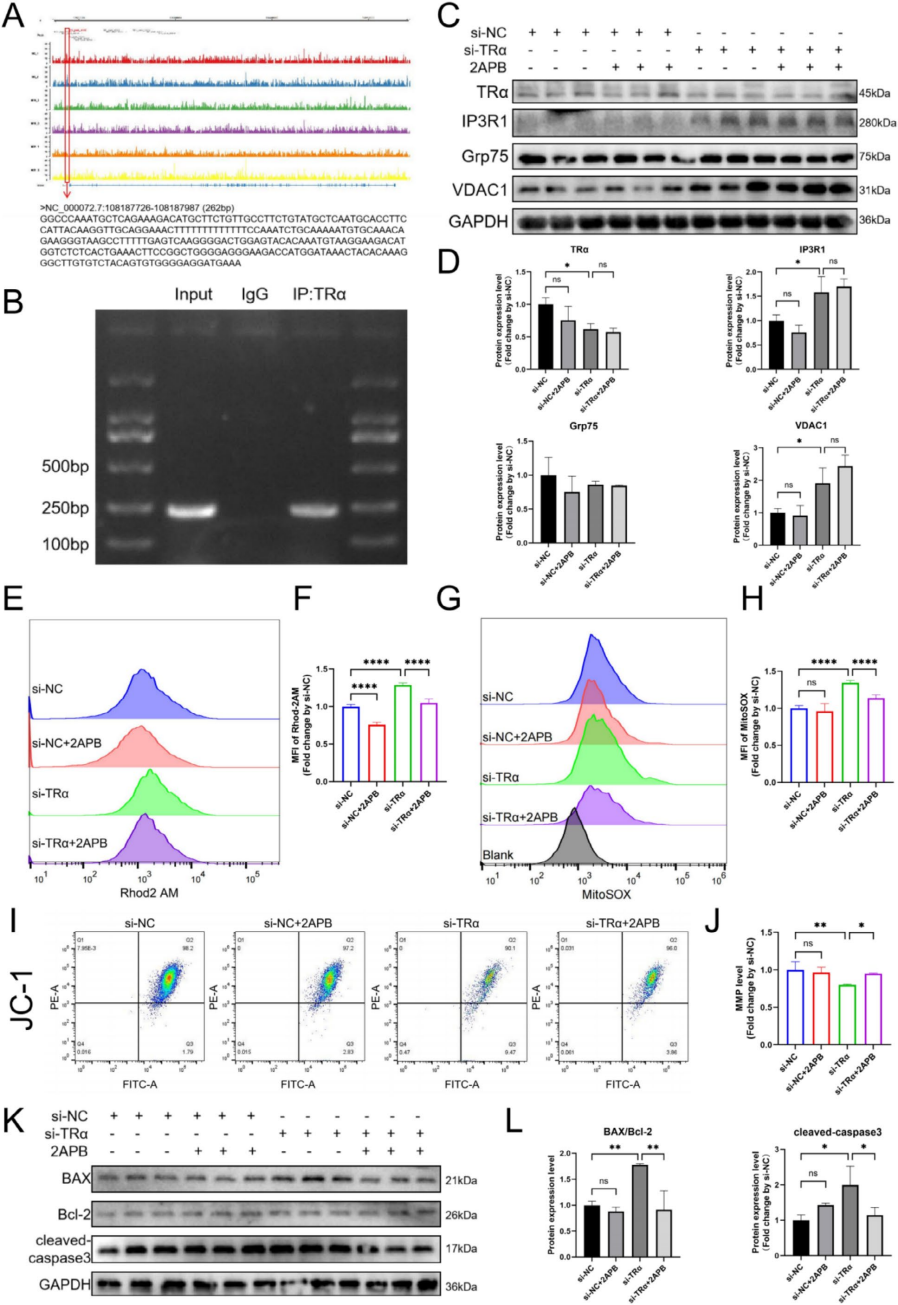
TRα knockdown activates IP3R1 transcriptional expression to induce mitochondrial Ca^2+^ overload in myoblasts. (A) The sequence near the IP3R1 promoter which TRα could bind from ChIP‐seq, and ChIP‐qPCR (B) analysis with anti‐TRα for it. (C, D) TRα, IP3R1, Grp75, VDAC1 and GAPDH protein expressions measured using WB in C2C12 cells with corresponding statistics (*n* = 3). (E, F) Flow cytometry results showing the fluorescence intensity of Rhod2 AM (*n* = 6). (G, H) Flow cytometry results showing the fluorescence intensity of MitoSOX (*n* = 6). (I, J) Flow cytometry results showing the red‐green fluorescence ratio intensity of JC‐1 (*n* = 6). (K, L) BAX, Bcl‐2, cleaved‐caspase3 and GAPDH protein expressions measured using WB in different group with corresponding statistics (*n* = 3). One‐way ANOVA; *p < 0.05, **p < 0.01, and ****p < 0.0001.

ChIP‐seq analysis identified a potential binding sequence of TRα on the IP3R1 promoter region (Figure 6A), and ChIP‐qPCR confirmed TRα's binding to this region (Figure 6B). Previous results have shown that IP3R1 expression significantly increases following TRα knockdown, suggesting that TRα binds to the IP3R1 promoter and negatively regulates its transcription.

IP3R inhibitors were used to determine if TRα deletion triggered mitochondrial Ca^2+^ overload via IP3R1. WB showed that 2APB did not alter IP3R1, Grp75, and VDAC1 protein levels (Figure 6C,D). The increase in Rhod2 AM MFI induced by TRα knockdown was reversed by 2APB (Figure 6E,F), as were the increases in MitoSOX levels and decreases in MMP (Figure 6G–J). Additionally, 2APB inhibited the increase in BAX/Bcl‐2 and cleaved‐caspase3 protein levels in TRα knockdown cells (Figure 6K,L). These results suggest that TRα knockdown induces mitochondrial Ca^2+^ overload through the activation of IP3R1 transcription in myoblasts.

### 3.7 Restoring TRα Expression Improves MAM‐Mediated Mitochondrial Ca^2+^ Overload in Aged Myoblasts

**FIGURE 7:**
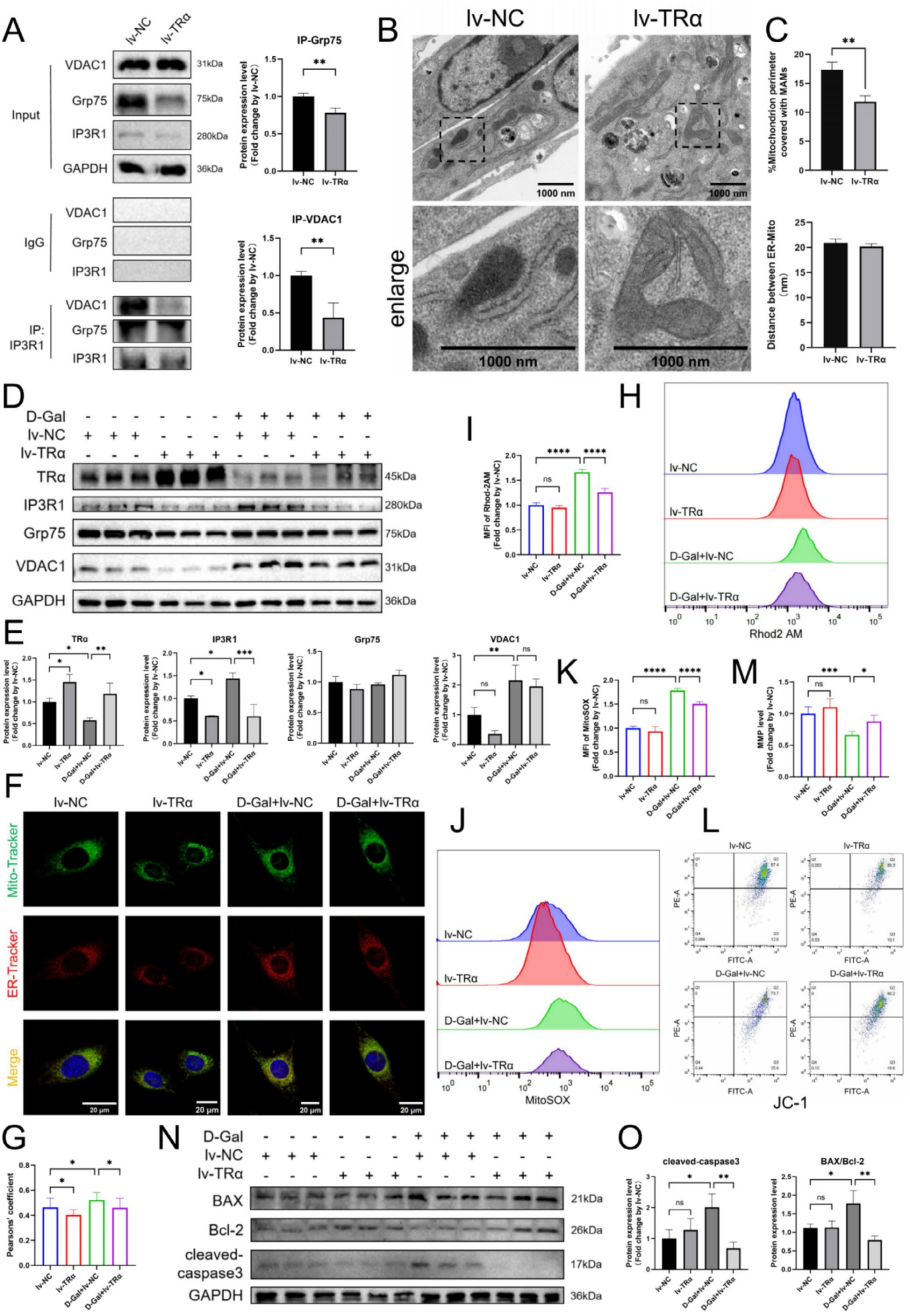
Restoring TRα expression improves MAM‐mediated mitochondrial Ca^2+^ overload in aged myoblasts. (A) CO‐IP analysis of IP3R1 interacting with Grp75, VDAC1 and GAPDH in C2C12 cells with corresponding statistics (*n* = 3). (B) Representative TEM images from C2C12 cells used to analyse MAMs, and MAMs were shown in yellow (scale bar: 1000 nm). (C) Effect of TRα overexpression on MAM coverage and thickness in C2C12 cells (*n* = 3). (D, E) TRα, IP3R1, Grp75, VDAC1 and GAPDH protein expressions measured using WB in C2C12 cells with corresponding statistics (*n* = 3). (F, G) ER‐Tracker Red and Mito‐Tracker Green co‐labelling to observe MAMs, and Pearson's correlation coefficient was calculated (scale bar: 20 μm, *n* = 3). (H, I) Flow cytometry results showing the fluorescence intensity of Rhod2 AM (*n* = 6). (J, K) Flow cytometry results showing the fluorescence intensity of MitoSOX (*n* = 6). (L, M) Flow cytometry results showing the red‐green fluorescence ratio intensity of JC‐1 (*n* = 6). (N, O) BAX, Bcl‐2, cleaved‐caspase3 and GAPDH protein expressions measured using WB in different group with corresponding statistics (*n* = 3). One‐way ANOVA; *p < 0.05, **p < 0.01, ***p < 0.001 and ****p < 0.0001.

A TRα overexpression model in C2C12 cells was established using a plasmid, with successful construction confirmed by PCR and WB. Post‐overexpression, TRα significantly decreased mRNA and protein levels of IP3R1, while Grp75 mRNA levels remained unchanged and its protein levels increased. VDAC1 mRNA levels were significantly elevated, yet its protein levels significantly decreased (Figure S5A–C). Furthermore, interactions of IP3R1 with Grp75/VDAC1 were significantly reduced (Figure 7A). TEM revealed a significant reduction in MAM coverage in TRα overexpression cells, with no significant change in thickness (Figure 7B,C). Co‐staining with Mito‐Tracker and ER‐Tracker showed a significant decrease in the Pearson correlation coefficient between ER and mitochondria (Figure S5D,E). Unexpectedly, the fluorescence intensity of Rhod2 AM, MitoSOX, JC‐1 red‐green fluorescence ratio, co‐staining of TOM20 and CytC, and the protein levels of BAX/Bcl‐2 and cleaved‐caspase3 did not change significantly post‐overexpression (Figure S5F–O).

Overexpression of TRα in D‐Gal‐induced senescent myoblasts significantly inhibited the increase in IP3R1 protein levels (Figure 7D,E). Co‐staining with Mito‐Tracker and ER‐Tracker demonstrated a significant inhibition in the increase of the Pearson correlation coefficient between mitochondria and ER (Figure 7F,G). TRα overexpression also significantly inhibited the increase in Rhod2 AM and MitoSOX levels in senescent cells (Figure 7H–K), and ameliorated the decline in MMP (Figure 7L,M). Additionally, TRα overexpression significantly inhibited the increase in BAX/Bcl‐2 and cleaved‐caspase3 expression in senescent myoblasts (Figure 7N,O).

These findings indicate that restoring TRα expression can inhibit the increased expression of IP3R1, Ca^2+^ transfer in MAM, and improve mitochondrial Ca^2+^ overload in senescent myoblasts.

## 4 Discussion

This study investigates the molecular mechanisms by which TRα regulates skeletal muscle ageing. The principal observations include: (1) A marked reduction in TRα expression both in vitro and in sarcopenia animal models, where its diminished expression contributes to muscle atrophy through the worsening of mitochondrial dysfunction and subsequent apoptosis, presumably via MAM‐mediated mitochondrial calcium overload; (2) Diminished TRα levels led to an upregulation of IP3R1 transcription, enhancing the interaction of IP3R1 to Grp75 and VDAC1, thereby facilitating increased calcium transfer in MAMs, and resulting in mitochondrial calcium overload; (3) Restoration of TRα expression effectively mitigated mitochondrial calcium overload and apoptosis in aged myoblasts by inhibiting IP3R1‐mediated MAM Ca^2+^ transfer.

Sarcopenia is a systemic skeletal muscle disease that emerges due to ageing, significantly impairing the quality of life and lifespan in the elderly [1]. Numerous studies have indicated that mitochondrial dysfunction and apoptosis accelerate skeletal muscle ageing [10, 31, 32]. Mitochondrial damage may trigger abnormal activation of intrinsic apoptosis [33]. In this study, increased mitochondrial ROS levels, abnormal mitochondrial morphology, and CytC localization, as well as enhanced intrinsic apoptosis were observed in skeletal muscle of aged mice and D‐Gal‐induced aged C2C12 cells. Serving as the principal receptor of THs in skeletal muscle, TRα is crucial for the growth and development of skeletal muscle and the regulation of energy metabolism [34]. TRα, a nuclear transcription factor, represses gene transcription by binding to co‐repressors in the absence of THs. However, the binding of THs to TRα alters its conformation and recruits co‐transcriptional activators, thereby activating gene transcription [24]. Previous studies have shown that TRα knockdown impairs the proliferation and differentiation of mouse myoblasts, significantly reduces mitochondrial numbers, inhibits oxidative respiration, and decreases mitophagy [26]. Furthermore, TRα expression was found to be down‐regulated in aged skeletal muscle [35], a finding reconfirmed in this study. These pieces of evidence suggest that TRα down‐regulation during ageing may contribute to the development of sarcopenia by impairing mitochondrial function.

To elucidate the specific mechanism of TRα‐mediated mitochondrial dysfunction in skeletal muscle ageing, ChIP‐seq analysis was initially conducted, revealing that TRα target genes were predominantly involved in the Ca^2+^ signalling pathway. Concurrently, GSEA of RNA‐seq data indicated that mitochondrial Ca^2+^ transmembrane transport, associated with the Ca^2+^ signalling pathway, was significantly upregulated in aged skeletal muscle. Ca^2+^ is absorbed by the outer membrane protein VDAC1 and transported into the mitochondria by the mitochondrial calcium uniporter (MCU), then expelled by the sodium‐Ca^2+^ exchanger and the mitochondrial permeability transition pore [12]. Ca^2+^ plays a crucial role in maintaining mitochondrial respiratory chain function and membrane potential, and in regulating mitophagy and mitochondrial dynamics [36]. Excessive or insufficient Ca^2+^ impacts normal mitochondrial function [5, 6]. Currently, the age‐related changes in mitochondrial Ca^2+^ content are debated. Some studies suggest a decline in MCU's ability to import Ca^2+^ during ageing reduces mitochondrial Ca^2+^ content [37], whereas others indicate that an abnormal increase of Ca^2+^ in aged cells causes mitochondrial Ca^2+^ overload, leading to increased mitochondrial ROS, decreased MMP, and damage to the outer membrane, which triggers the release of mitochondrial content and activates the intrinsic apoptotic pathway [38, 39, 40]. This study's significant upregulation of mitochondrial Ca^2+^ transmembrane transport suggests abnormal activation of Ca^2+^ influx or efflux in aged skeletal muscle, resembling mitochondrial Ca^2+^ overloading observed in GA muscle of old mice and senescent C2C12 cells, with increased mitochondrial Ca^2+^ in aged cells. All of these indicates that abnormal activation of mitochondrial Ca^2+^ influx leads to mitochondrial Ca^2+^ overload in aged skeletal muscle.

Mitochondrial Ca^2+^ primarily originates from the ER, transmitted through MAM, a specialised region linking the ER and mitochondria, primarily formed by anchoring complexes such as IP3R/Grp75/VDAC1 and Mfn1/2. IP3R/Grp75/VDAC1 is both a crucial complex for MAM formation and a major carrier of Ca^2+^ flux within MAM. Ca^2+^ release from the ER is transferred to the outer mitochondrial membrane via Grp75 and subsequently into the mitochondria through VDAC1, forming part of the MAM calcium flux pathway [41]. The operational efficiency of the IP3R/Grp75/VDAC1 complex, as well as the quantity and structural integrity of the MAMs, is a crucial determinant of calcium transfer efficacy from the ER to mitochondria [42]. ChIP‐seq analysis suggested IP3R1 as a potential target gene of TRα, implicating it as a key gene in regulating mitochondrial Ca^2+^ overload in skeletal muscle. As an ER membrane protein that regulates Ca^2+^ release, IP3R acts as a gating switch for Ca^2+^ flow in MAM and is vital for Ca^2+^ transport between the ER and mitochondria [18, 43]. Previous research has demonstrated that IP3R knockdown significantly reduces Ca^2+^ increases in mitochondria and MAM formation in replicative senescent fibroblasts [44]. Additionally, IP3R knockout mice exhibited a longer lifespan, reduced IP3R/Grp75/VDAC1 complex and MAMs, and improved mitochondrial function [20]. In this study, increased IP3R1, IP3R1/Grp75/VDAC1 complex, MAMs, and mitochondrial Ca^2+^ overload were observed in D‐Gal‐induced aged C2C12 cells, and the IP3R inhibitor 2APB improved mitochondrial Ca^2+^ overload. Increased expression of IP3R1 in the GA muscle of aged mice, along with a significant rise in IP3R1/Grp75/VDAC1 complex and MAM numbers, suggests that abnormal IP3R1 activation contributes to mitochondrial Ca^2+^ overload in aged skeletal muscle cells by enhancing Ca^2+^ transfer in MAM.

We generated TRα skeletal muscle‐specific knockout mice to clarify the effects of TRα deficiency on skeletal muscle. Previous studies have used TRα transcriptional mutant mice due to the lethality of TRα knockout mice post‐birth [45]; hence, we opted for a skeletal muscle‐specific knockout approach. The knockout mice survived successfully post‐birth. At 8 weeks of age, no significant change in grip strength was observed, but a notable decrease in GA muscle mass and muscle fibre size indicated that TRα deletion indeed caused skeletal muscle atrophy in mice. Increased IP3R1 expression in the skeletal muscle of these mice confirmed TRα's regulatory role. Additionally, increased protein expression of Grp75 and VDAC1 was observed, though mRNA levels did not change significantly, and the interaction between IP3R1 and these proteins was significantly enhanced, suggesting that the protein increases were mainly due to IP3R1 up‐regulation rather than TRα transcriptional activity. The increase in MAMs, decreased thickness of MAMs, and mitochondrial Ca^2+^ overload‐like changes indicated enhanced MAM Ca^2+^ transfer in the skeletal muscle of knockout mice. Interestingly, the expression of pro‐apoptotic factors BAX and cleaved‐caspase3 and TUNEL positivity suggested increased apoptosis in the skeletal muscle of knockout mice, while the significant up‐regulation of the anti‐apoptotic factor Bcl‐2 might represent a compensatory inhibition of apoptosis in young mice. The PI3K/Akt signalling pathway regulates cell proliferation and apoptosis [46], and is critical for the pathogenesis and progression of sarcopenia [47]. We found that the phosphorylation level of Akt was significantly increased in the skeletal muscle of SKT mice. This increase may activate anti‐apoptotic factors such as Bcl‐2 [48, 49], consistent with the unexpected elevation of Bcl‐2 expression observed in knockout mice. These findings suggest that activation of the PI3K/Akt signalling pathway may serve as an upstream compensatory mechanism underlying the marked upregulation of the anti‐apoptotic factor Bcl‐2 in skeletal muscle from knockout mice. However, further experiments are needed to confirm this. These findings demonstrate for the first time that TRα deficiency induces skeletal muscle atrophy in mice by exacerbating mitochondrial damage and apoptosis, potentially due to MAM‐mediated mitochondrial Ca^2+^ overload.

Further study was conducted on the regulatory role of IP3R1 in MAM calcium transfer by TRα in vitro. ChIP‐qPCR demonstrated that TRα could attach to the promoter region of IP3R1. Cell models with either reduced or increased TRα expression revealed significant alterations in IP3R1 expression and its interaction with Grp75/VDAC1, along with changes in the number of MAMs, suggesting that TRα negatively influences IP3R1 transcription. This modulation affects the formation of the IP3R1/Grp75/VDAC1 complex and MAMs. In TRα knockdown cells, mitochondrial Ca^2+^ significantly increased, along with mitochondrial damage and apoptosis, which could be inhibited by 2APB. Interestingly, TRα overexpression did not reduce mitochondrial Ca^2+^ content or improve mitochondrial function, possibly due to physiological cellular autoregulation. However, TRα overexpression in senescent cells inhibited the increase in IP3R1, suggesting that the increased expression of IP3R1 in ageing skeletal muscle is triggered by the down‐regulation of TRα. Additionally, TRα overexpression also inhibited the formation of the IP3R1/Grp75/VDAC1 complex and MAMs, and improved mitochondrial Ca^2+^ overload in senescent cells. These results suggest that TRα down‐regulation activates Ca^2+^ transfer in MAM by promoting IP3R1 transcription and induces mitochondrial Ca^2+^ overload. Restoring TRα expression can improve MAM‐mediated mitochondrial Ca^2+^ overload in aged mouse myoblasts.

Additionally, some potential limitations of this study should be noted. This study did not provide in vivo evidence that IP3R1 is the sole or most critical downstream regulatory target responsible for mitochondrial dysfunction and skeletal muscle atrophy caused by TRα deficiency in mice. Moreover, ageing is a complex process, and the causal relationship between decreased TRα expression and the ageing phenotype of mouse skeletal muscle remains incompletely elucidated. Additionally, ageing processes differ between mice and humans, and our findings have yet to be validated in human samples. However, patients with sarcopenia indeed exhibit significant apoptosis in skeletal muscle cells and mitochondrial dysfunction [50], which to some extent suggests potential clinical applicability of our results in humans. Furthermore, thyroid hormone receptor β agonists have been extensively developed and applied [51, 52]. Our results indicate that TRα deficiency exacerbates sarcopenia, highlighting the potential for developing and applying TRα agonists. This study employed three experimental models (natural ageing mice, skeletal muscle‐specific TRα knockout mice, and C2C12 myoblast cells) to investigate the role of TRα at multiple levels (animal disease model, tissue‐specific gene regulation, and cellular mechanism), thereby reducing potential bias associated with reliance on a single model. Considering the characteristics of TRα as a transcription factor, ChIP‐seq was selected to identify key TRα target sites involved in skeletal muscle ageing. For each specific observation index, multiple analytical methods were employed to ensure the reliability and robustness of experimental results. In future research, we will collect skeletal muscle biopsy samples from clinical sarcopenia patients to validate our findings in humans. Additionally, by using adeno‐associated virus‐mediated restoration of TRα expression in skeletal muscles of naturally ageing mice, we aim to further clarify the specific mechanisms underlying TRα downregulation in age‐related skeletal muscle atrophy.

## 5 Conclusion

In summary, TRα down‐regulation during ageing increases IP3R1 transcription and enhances the IP3R1/Grp75/VDAC1 interaction, thus promoting Ca^2+^ transfer in MAMs, ultimately triggering mitochondrial Ca^2+^ overload and apoptosis, leading to skeletal muscle atrophy. TRα/IP3R1‐mediated MAM Ca^2+^ transfer may be a promising target for mitigating the progression of sarcopenia in the future.

## Author Contributions

R.S. and Y.D. designed the study. R.S., Y.Z., and G.C. performed the animal experiments. R.S., G.C., and J.Z. performed the cell culture experiments. Y.Z. and J.Z. performed the ChIP‐seq and RNA‐seq analysis. Statistical analysis was performed by R.S., J.L., and H.Z. R.S., Y.D., and M.S. prepared the manuscript. All authors reviewed the manuscript.

## Conflicts of Interest

The authors declare no conflicts of interest.

## Supporting information


**APPENDIX S1:** Supporting information.

**FIGURE S1:** (A) mRNA levels of IP3R1 in GA muscle of mice at different months of age (*n* = 8). (B) GSEA on the GO of “mitochondria‐associated ER membrane (MAM)” is shown. (C, D) mRNA levels of Grp75 and VDAC1 in GA muscle of mice at different months of age (*n* = 8). (E) GSEA on the GO of “positive regulation of intrinsic apoptotic signalling pathway” is shown. One‐way ANOVA; *p < 0.05, **p < 0.01 and ****p < 0.0001.

**FIGURE S2:** 40 mg/mL is the optimal concentration of D‐Gal to induce C2C12 cell senescence. (A, B) Representative images of β‐galactosidase staining of C2C12 cells and corresponding statistics of the proportion of positive cells (scale bar: 200 μm, *n* = 3). (C) The cell survival rate was detected by CCK8 assay (*n* = 5). (D, E) p16, p21 and GAPDH protein expressions measured using WB in C2C12 cells with corresponding statistics (*n* = 3). (F) mRNA levels of p21, p53, IP3R1, Grp75 and VDAC1 of control and D‐Gal group in C2C12 cells (*n* = 6). *p < 0.05, ***p < 0.001 and ****p < 0.0001.

**FIGURE S3:** Identification of TRα skeletal‐muscle‐specific knockout mice. (A) Gel plot of mouse tail genotype identification. (B) mRNA level of TRα in GA muscle of mice (*n* = 6). (C) Protein level of TRα in GA muscle of mice (*n* = 3). (D, E) Body weight and grip strength of mice (*n* = 6). (F) mRNA levels of IP3R1, Grp75 and VDAC1 in GA muscle (*n* = 6). (G) Protein expression ratio of BAX to Bcl‐2 in mice GA muscle (*n* = 3). (H) mRNA levels of BAX, Bcl‐2 and caspase3 in GA muscle (*n* = 6). (I, J) Protein level of p16, p21, p53, PI3K, p‐Akt and Akt in GA muscle of mice (*n* = 3). Unpaired Student's *t*‐tests; *p < 0.05 and **p < 0.01.

**FIGURE S4:** (A) mRNA levels of TRα, IP3R1, Grp75 and VDAC1 in C2C12 cells (*n* = 6). (B, C) TRα and GAPDH protein expressions measured using WB in C2C12 cells with corresponding statistics (*n* = 3). (D, E) p16, p21, p53, PI3K, p‐Akt and Akt protein expressions measured using WB in C2C12 cells with corresponding statistics (*n* = 3). Unpaired Student's *t*‐tests; *p < 0.05 and **p < 0.01.

**FIGURE S5:** Overexpression of TRα reduced IP3R1 expression and MAMs formation. (A) mRNA levels of TRα, IP3R1, Grp75 and VDAC1 in C2C12 cells (*n* = 6). (B) TRα, IP3R1, Grp75, VDAC1 and GAPDH protein expressions measured using WB in C2C12 cells with corresponding statistics (*n* = 3). (D, E) ER‐Tracker Red and Mito‐Tracker Green co‐labelling to observe MAMs, and Pearson's correlation coefficient was calculated (scale bar: 20 μm, *n* = 3). (F, G) Representative images of Mito‐Tracker green co‐stained with Rhod2 AM staining and corresponding red fluorescence intensity analysis (scale bar: 100 μm, *n* = 3). (H, I) Results of MitoSOX staining and corresponding red fluorescence intensity analysis (scale bar: 50 μm, *n* = 3). (J, K) Results of JC‐1 staining and corresponding red‐green fluorescence ratio analysis (scale bar: 100 μm, *n* = 3). (L, M) Representative images of TOM20 co‐stained with CytC in C2C12 cells, and the ratio of CytC fluorescence that did not colocalize with TOM20 was counted (scale bar: 20 μm, *n* = 3). (N, O) BAX, Bcl‐2, and cleaved‐caspase3 protein expressions measured using WB in C2C12 cells with corresponding statistics (*n* = 3). Unpaired Student's *t*‐tests; *p < 0.05, **p < 0.01 and ****p < 0.0001.

**APPENDIX S2:** Supporting information.

## Data Availability

The data that support the findings of this study are openly available in ChIP‐seq and RNA‐seq analysis reveals the association between https://www.ncbi.nlm.nih.gov/geo/query/acc.cgi?acc=GSE281837.
