# Supplementary material for: Ageing‐Dependent Thyroid Hormone Receptor α Reduction Activates IP3R1‐Meditated Ca2+ Transfer in MAM and Exacerbates Skeletal Muscle Atrophy in Mice
Source: Cell Prolif. 2025 Aug 24;59(5):e70120. doi: 10.1111/cpr.70120 (PMC13114768; doi:10.1111/cpr.70120)
Supplement: Supplementary file 1 — APPENDIX S1: Supporting information. [file CPR-59-e70120-s001.pdf]

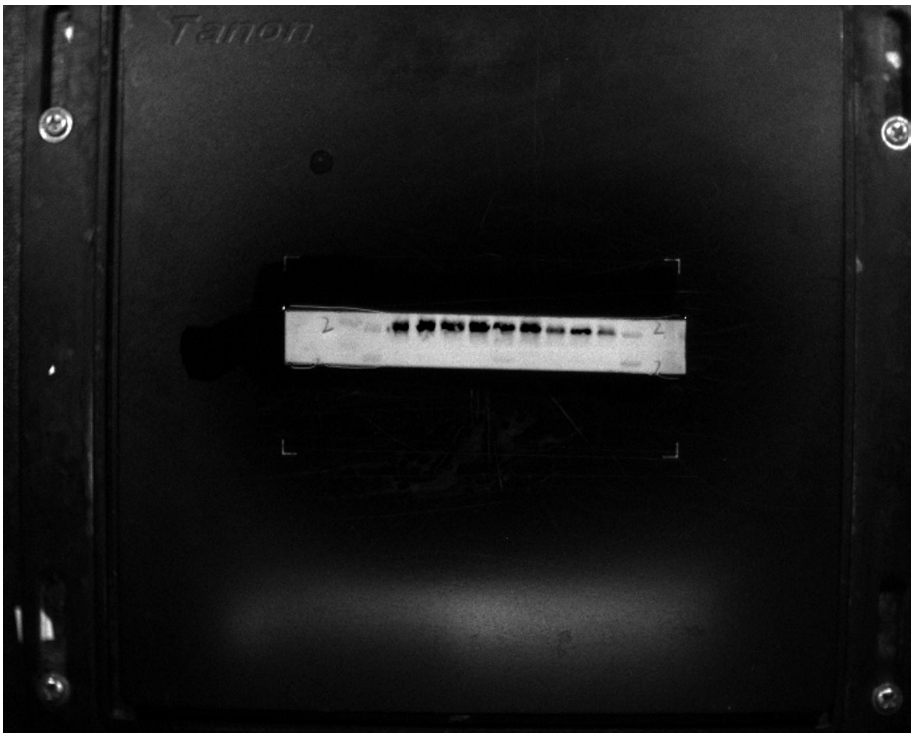

TRα for Fig. 1G.

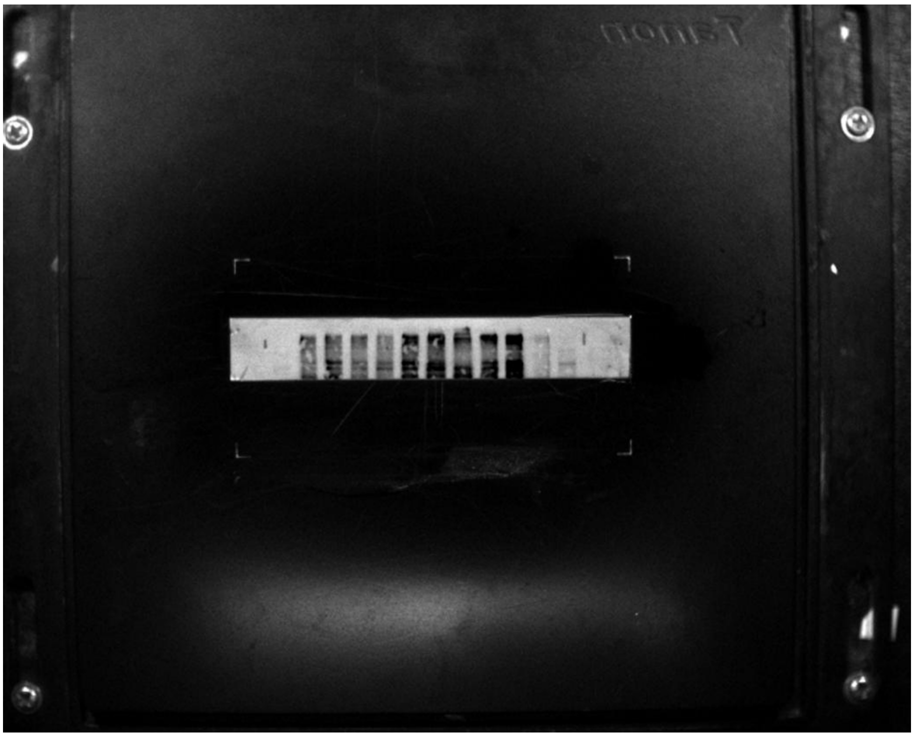

IP3R1 for Fig. 1G.

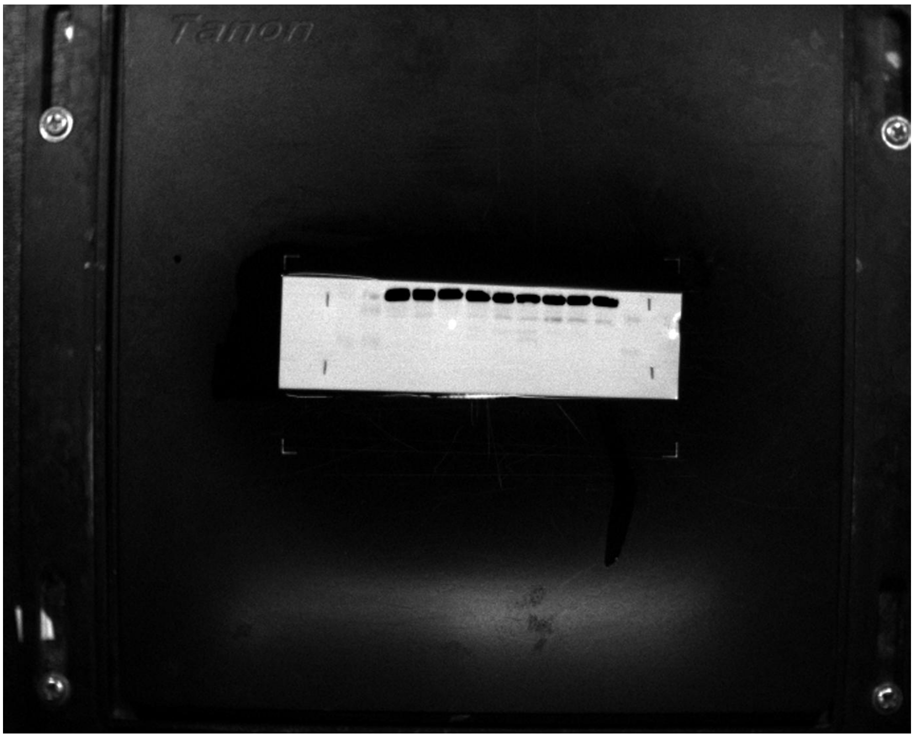

GAPDH for Fig. 1G.

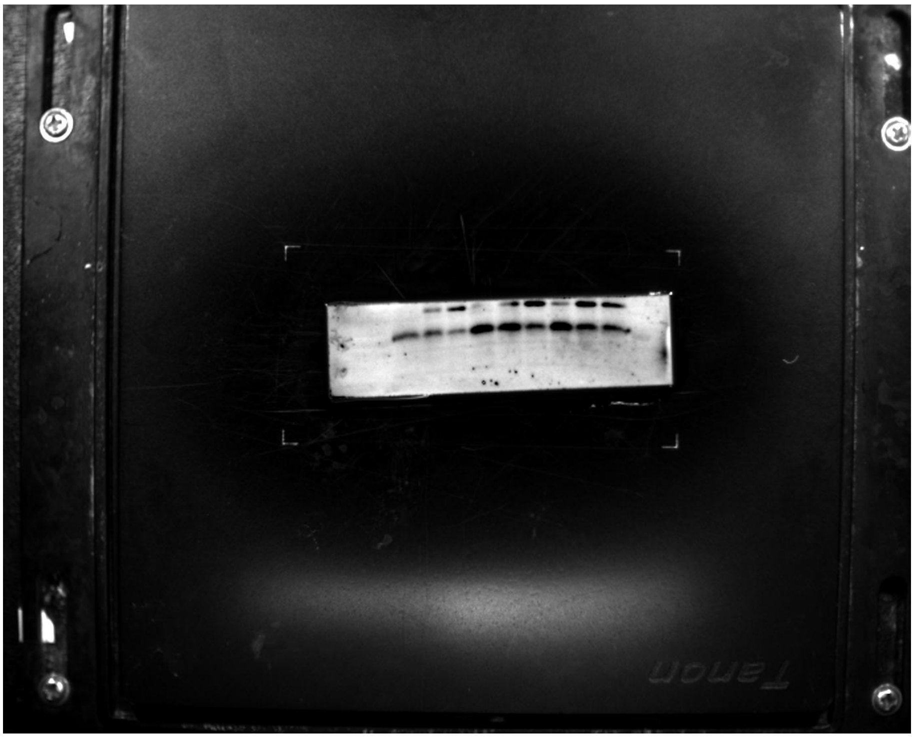

P16 for Fig. 1G.

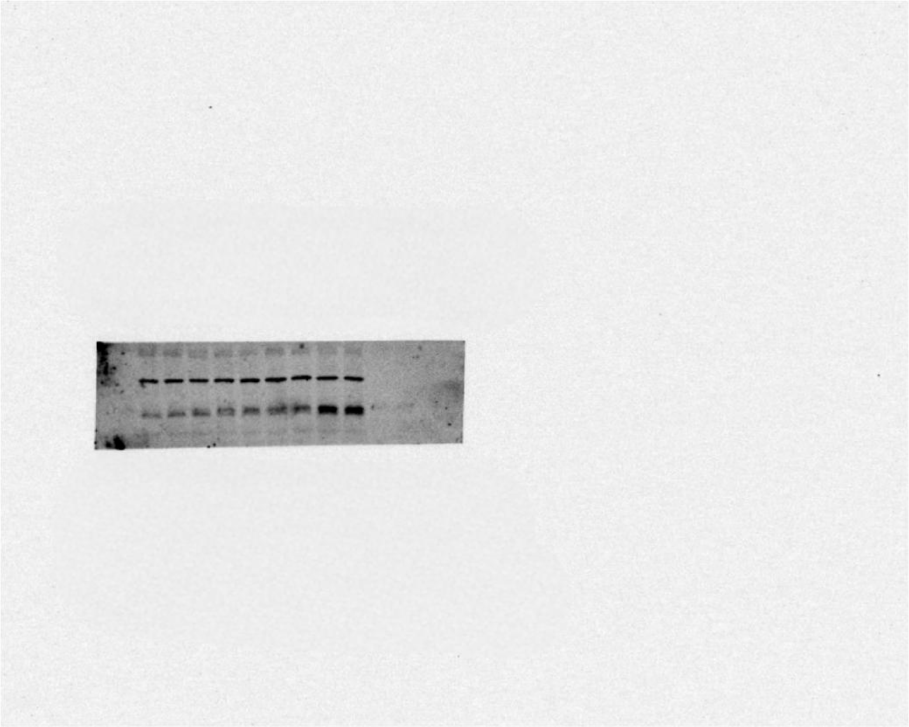

P21 and GAPDH for Fig. 1G.

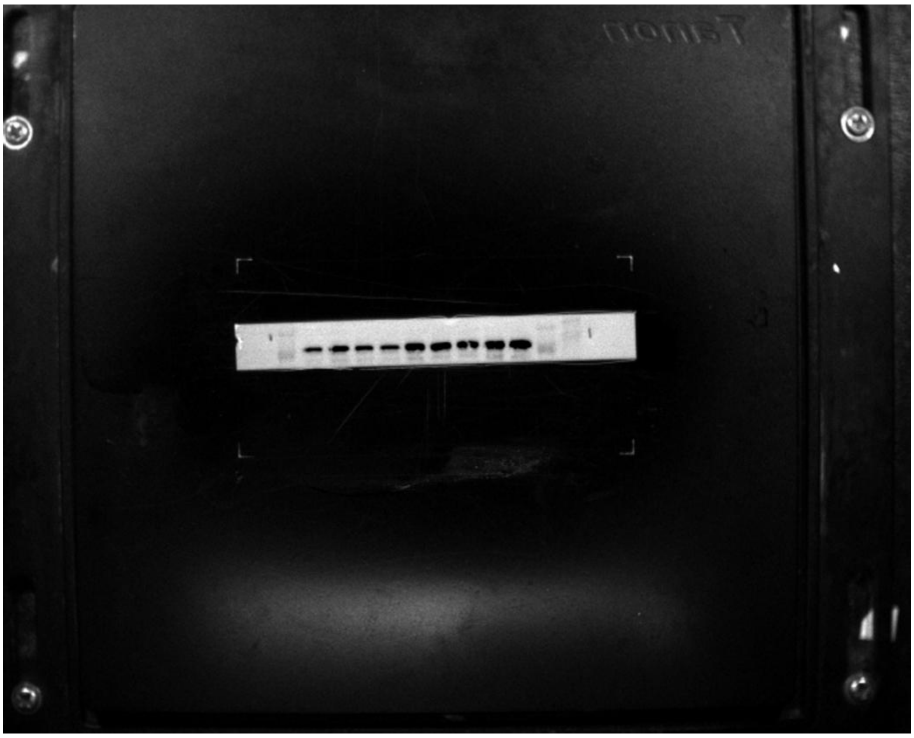

Grp75 for Fig. 2C.

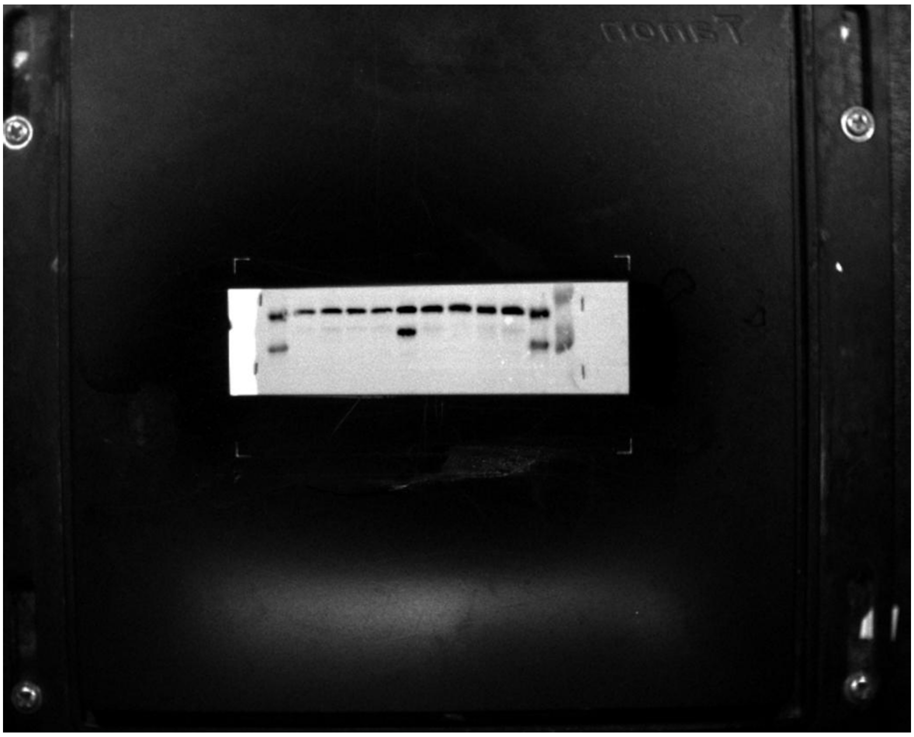

VDAC1 for Fig. 2C.

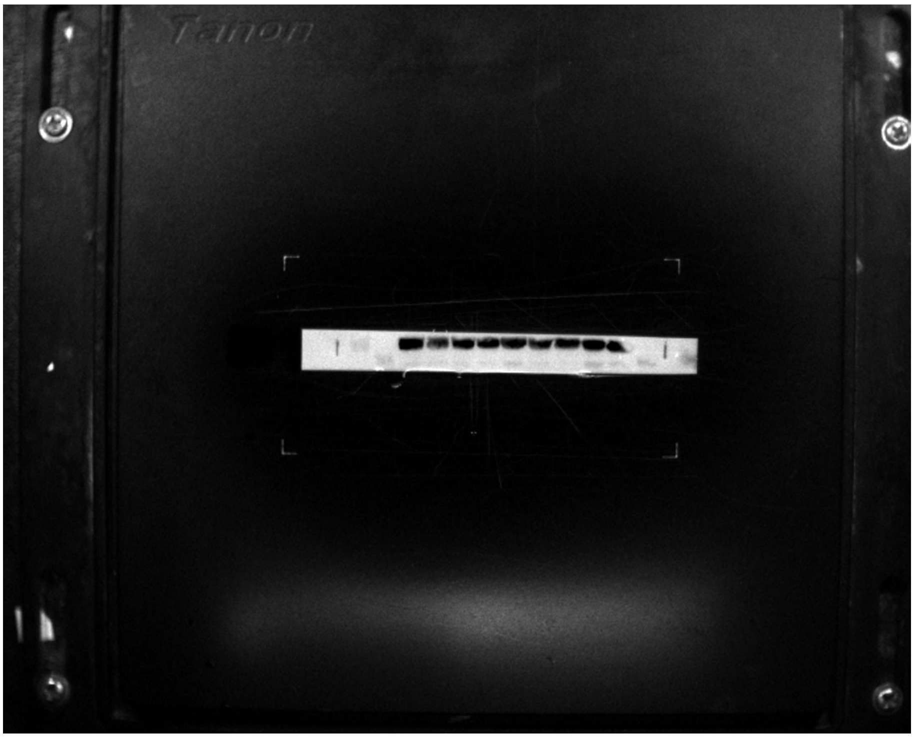

GAPDH for Fig. 2C.

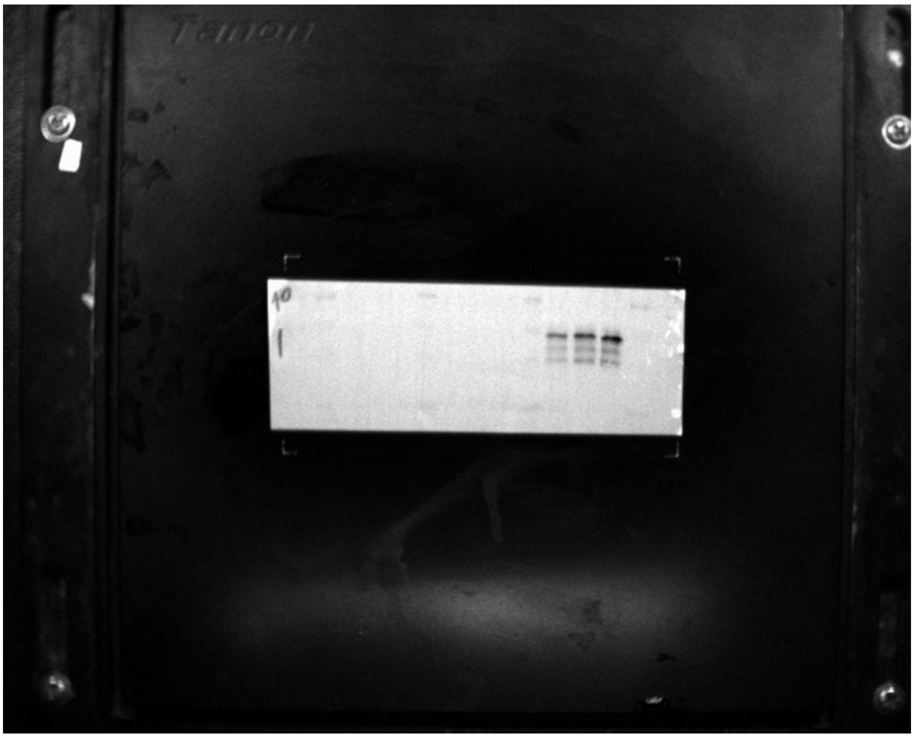

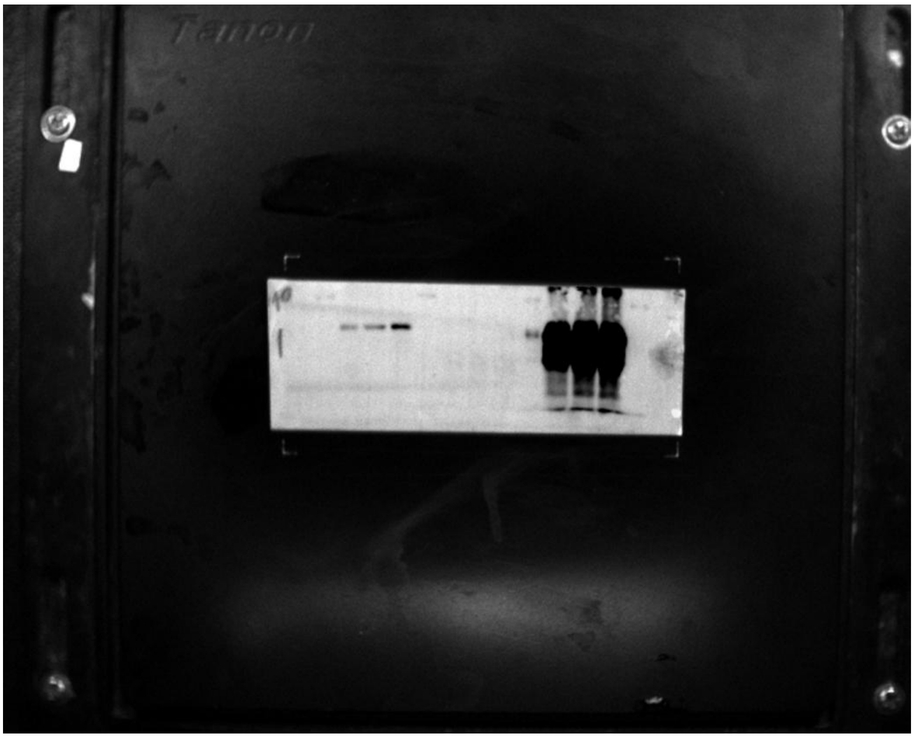

VDAC1 for Fig. 2E.

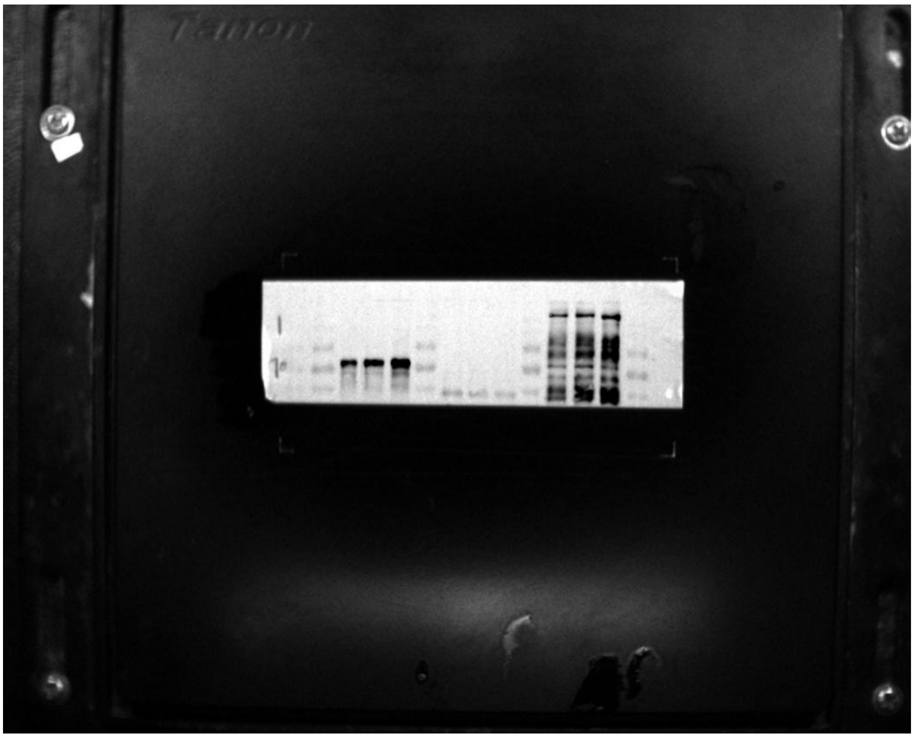

Grp75 for Fig. 2E.

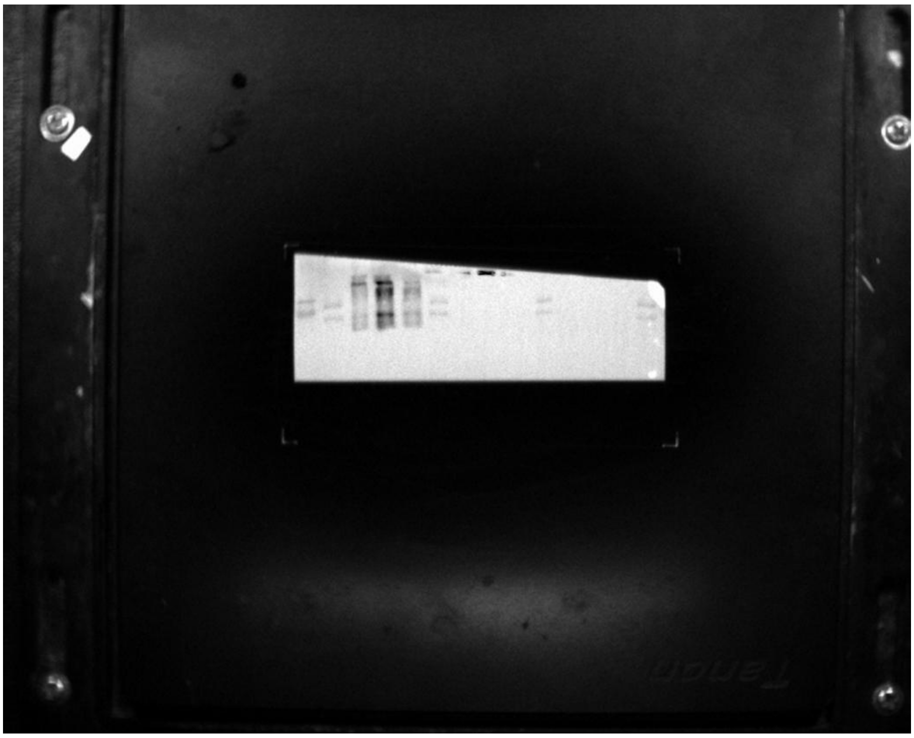

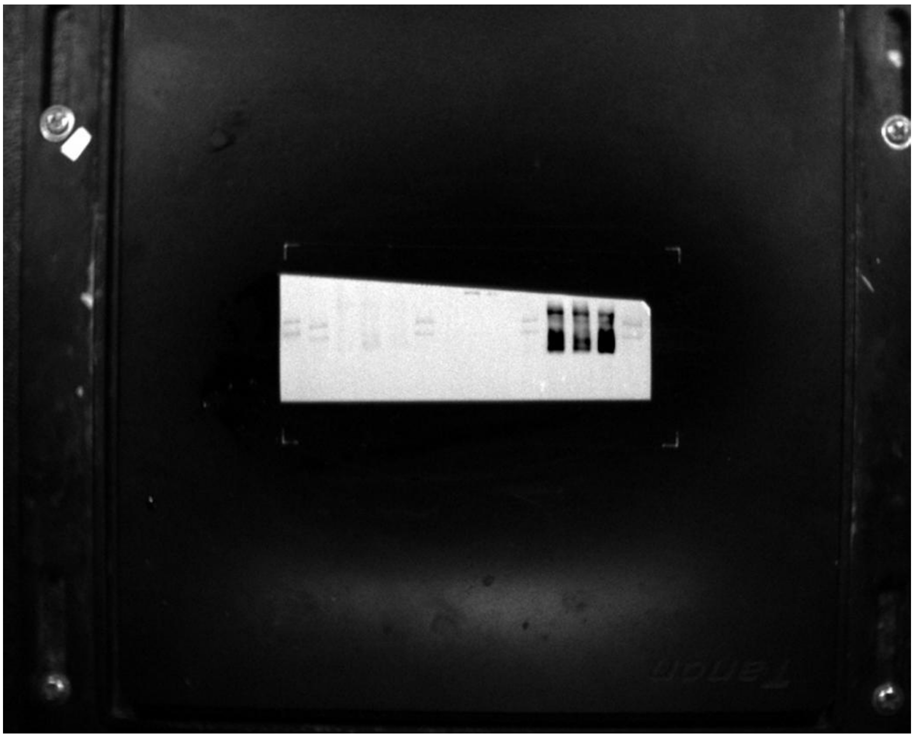

IP3R1 for Fig. 2E.

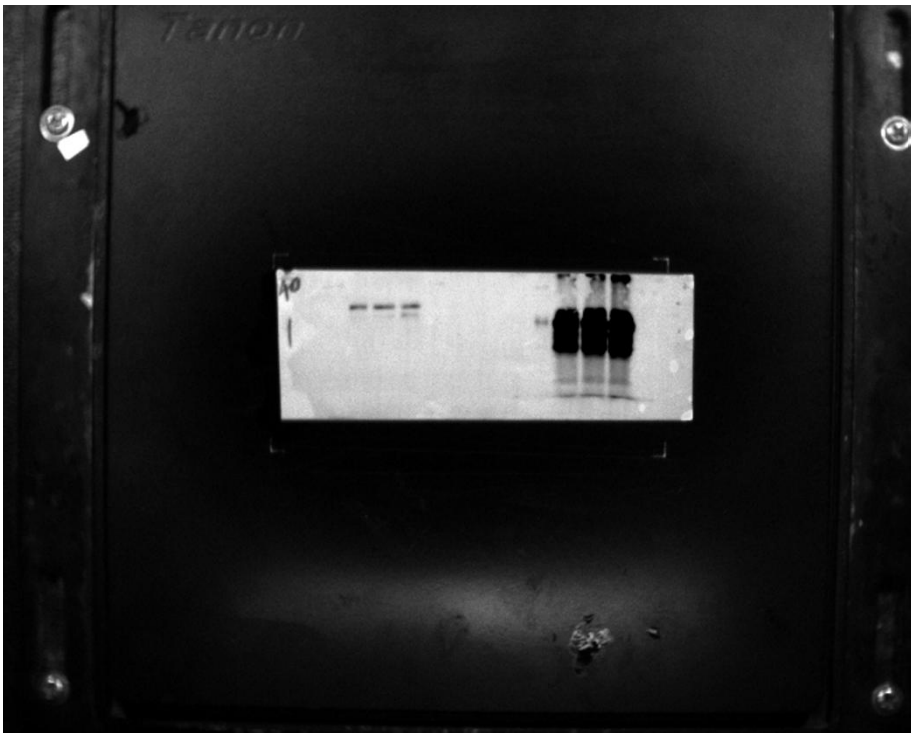

GAPDH for Fig. 2E.

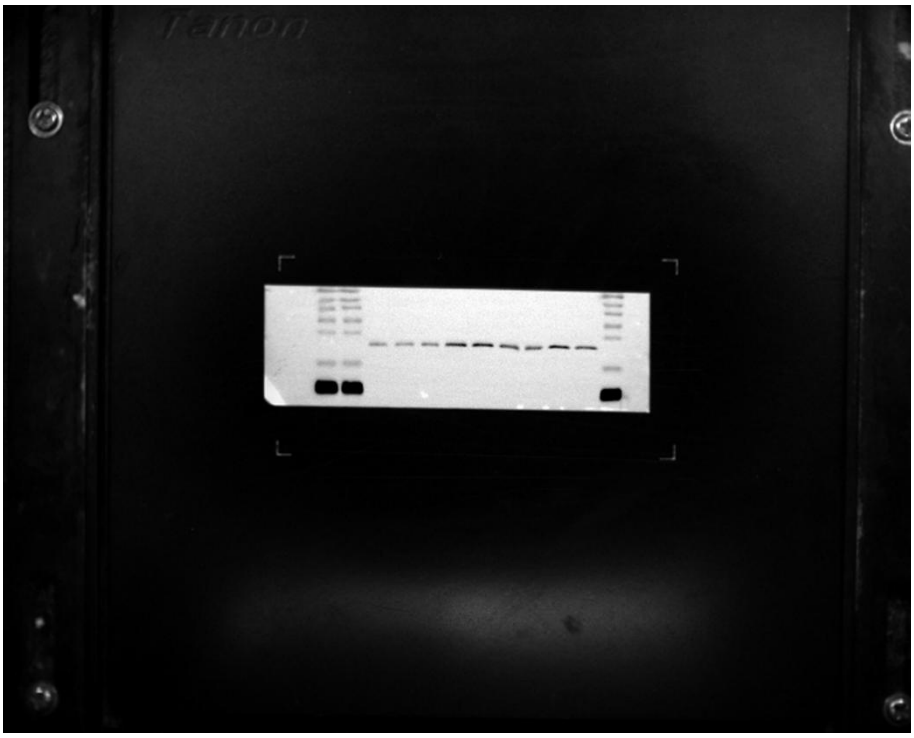

Bax for Fig. 2K.

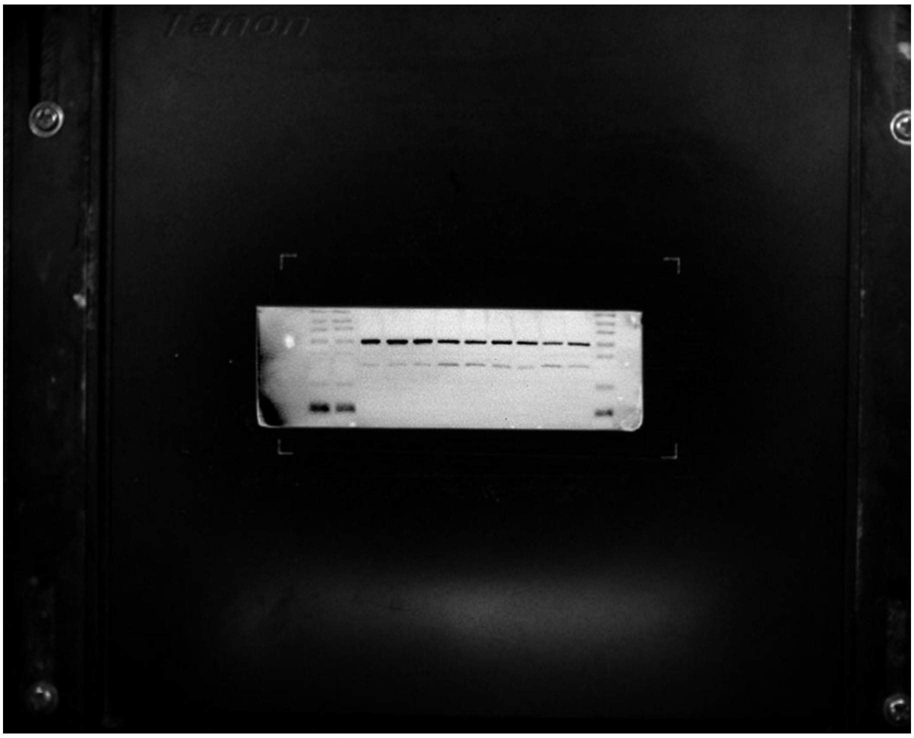

Bcl-2 for Fig. 2K.

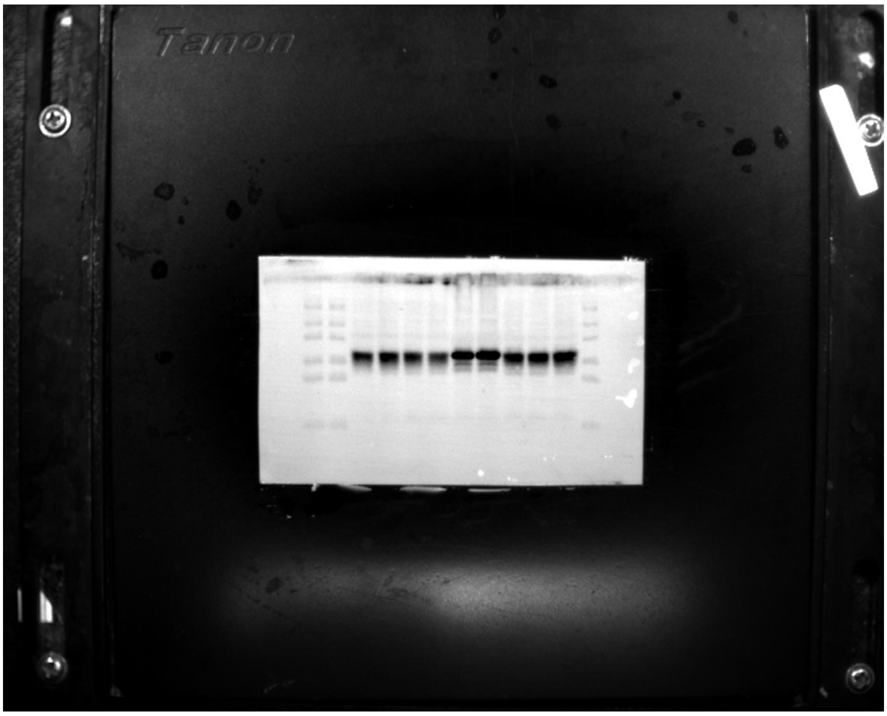

caspase3 for Fig. 2K.

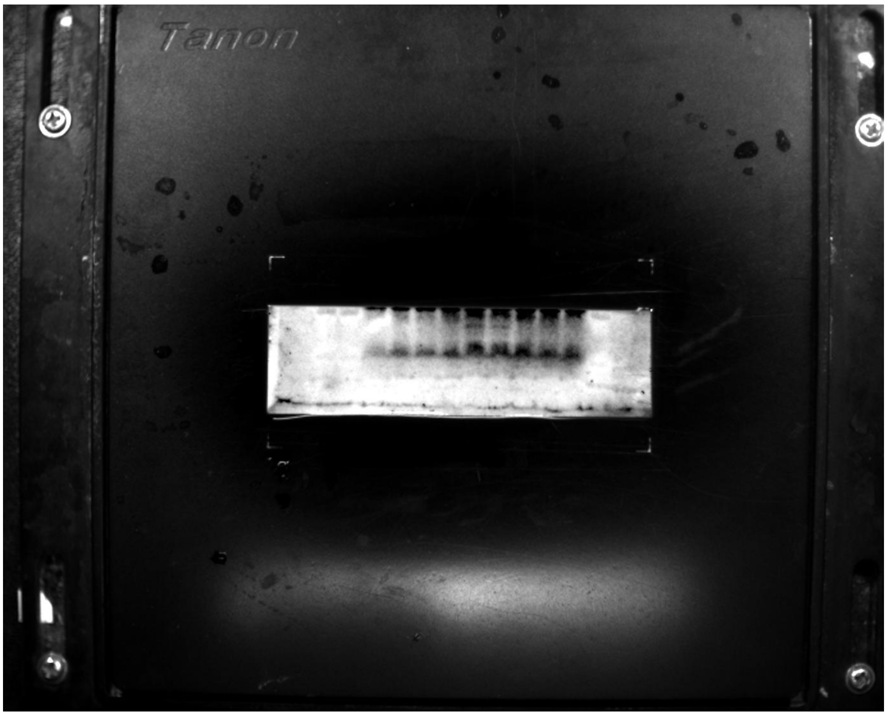

Cleaved caspase3 for Fig. 2K.

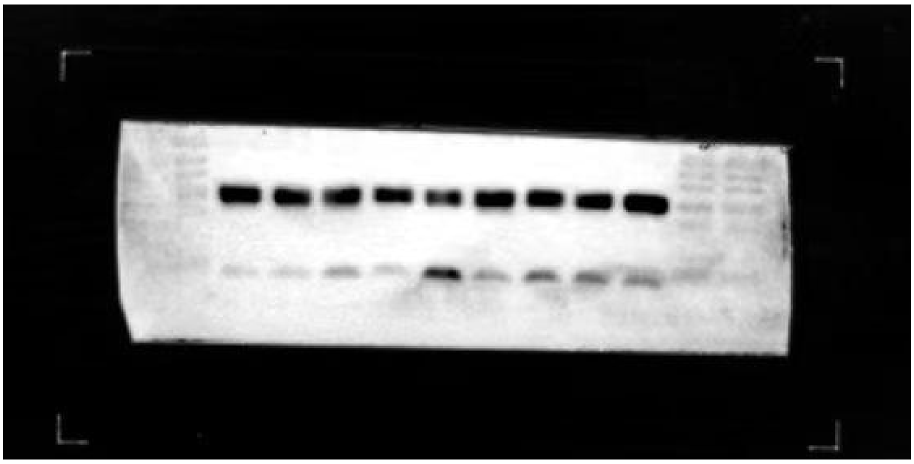

GAPDH for Fig. 2K.

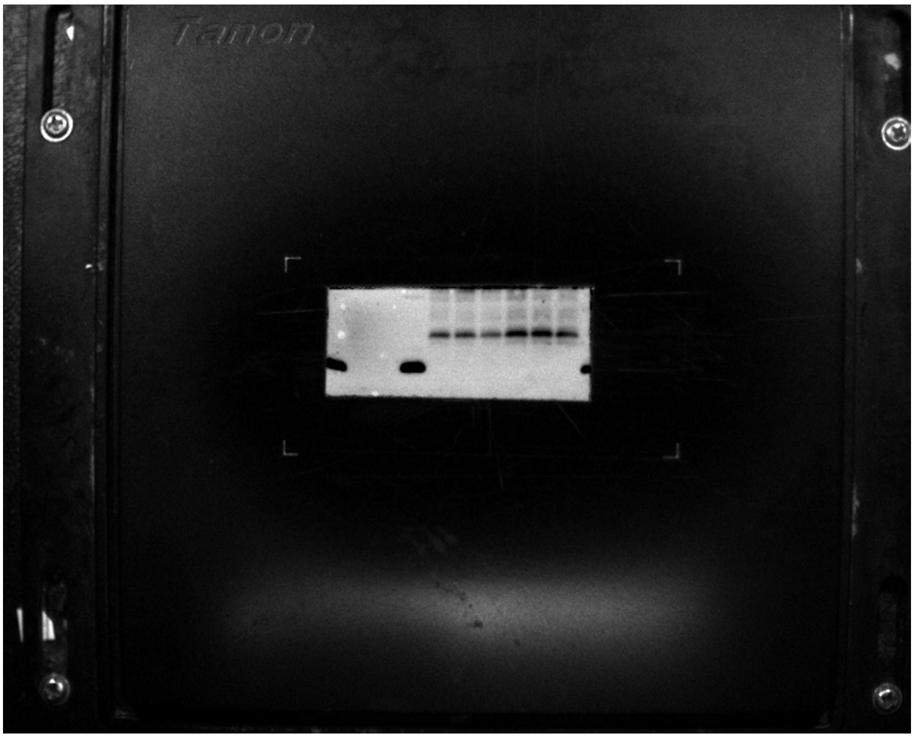

P16 for Fig. 3A.

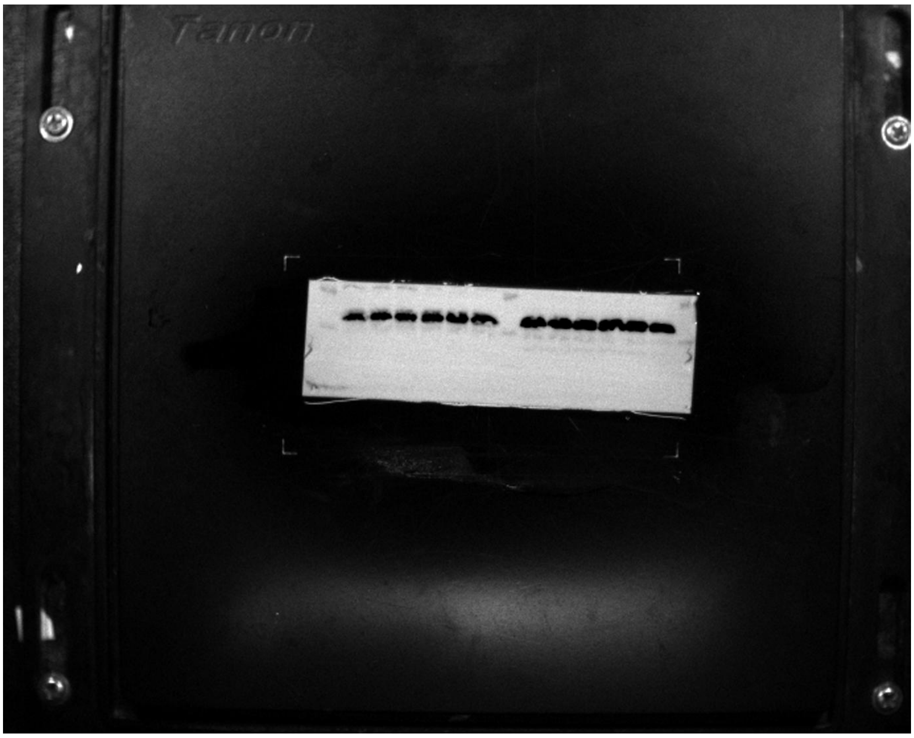

P21 for Fig. 3A.

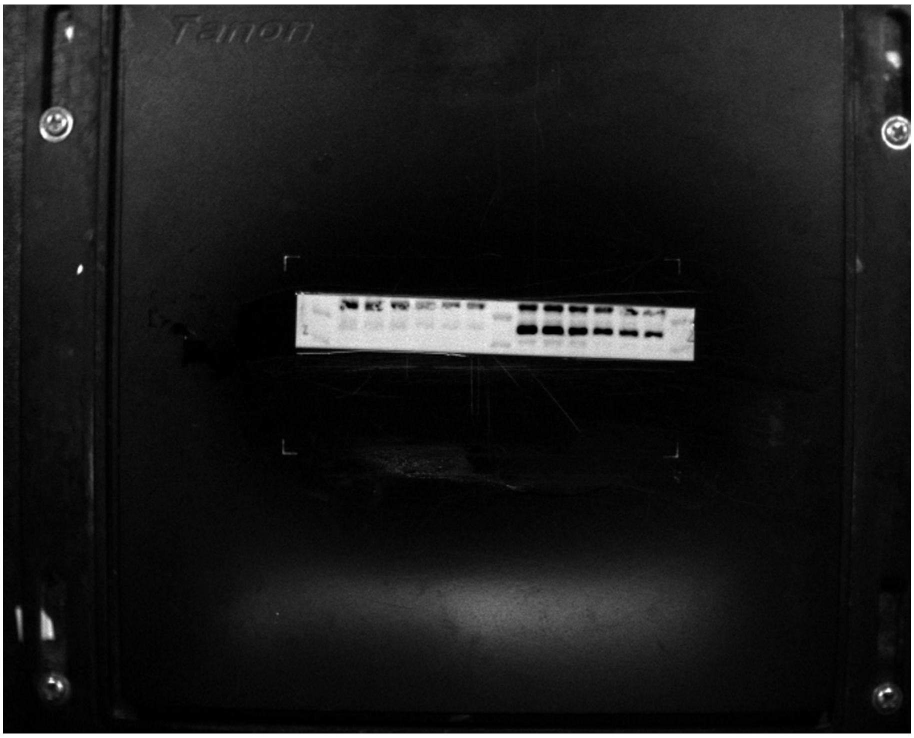

TRα for Fig. 3A.

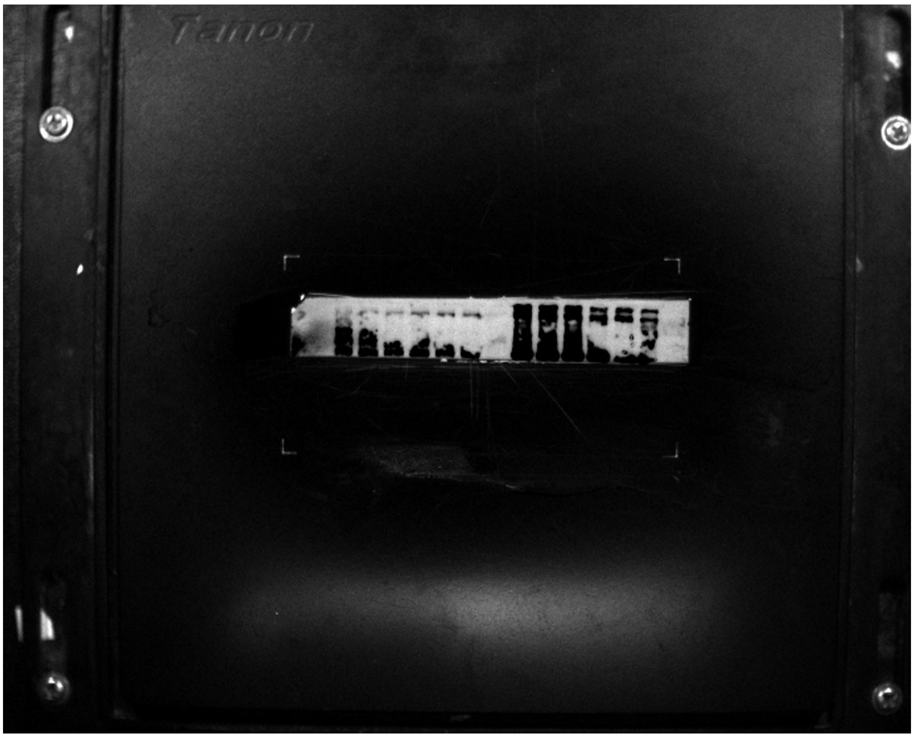

IP3R1 for Fig. 3A.

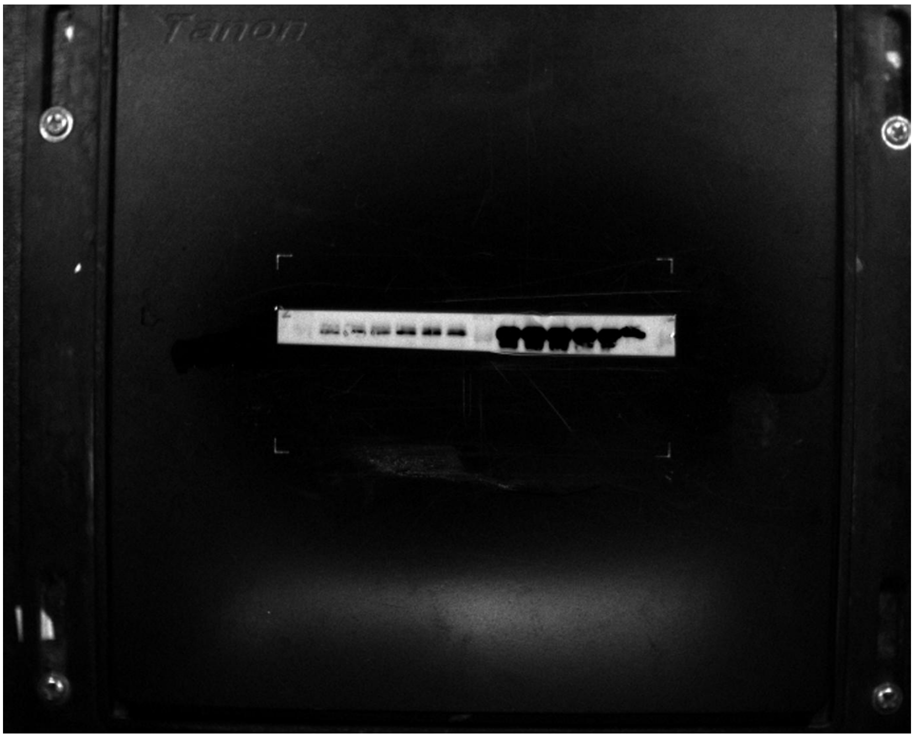

Grp75 for Fig. 3A.

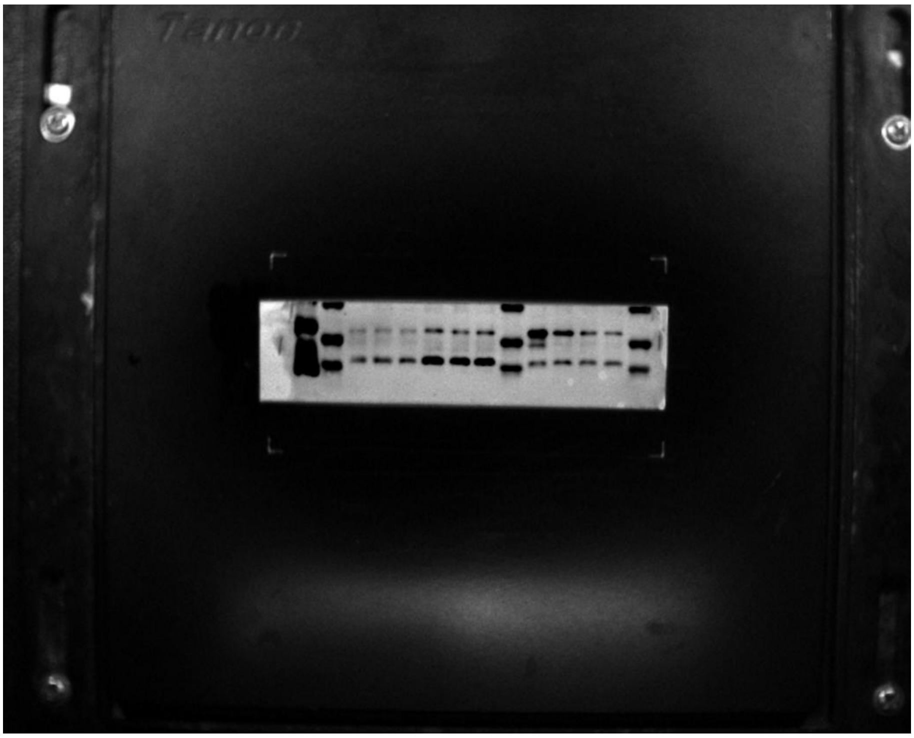

VDAC1 for Fig. 3A.

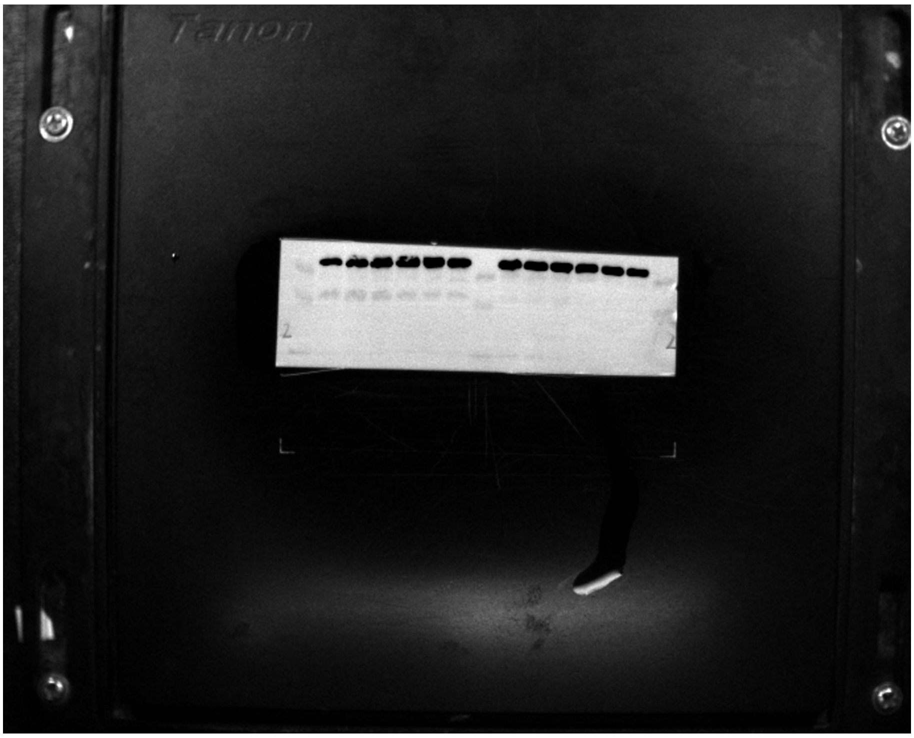

GAPDH for Fig. 3A.

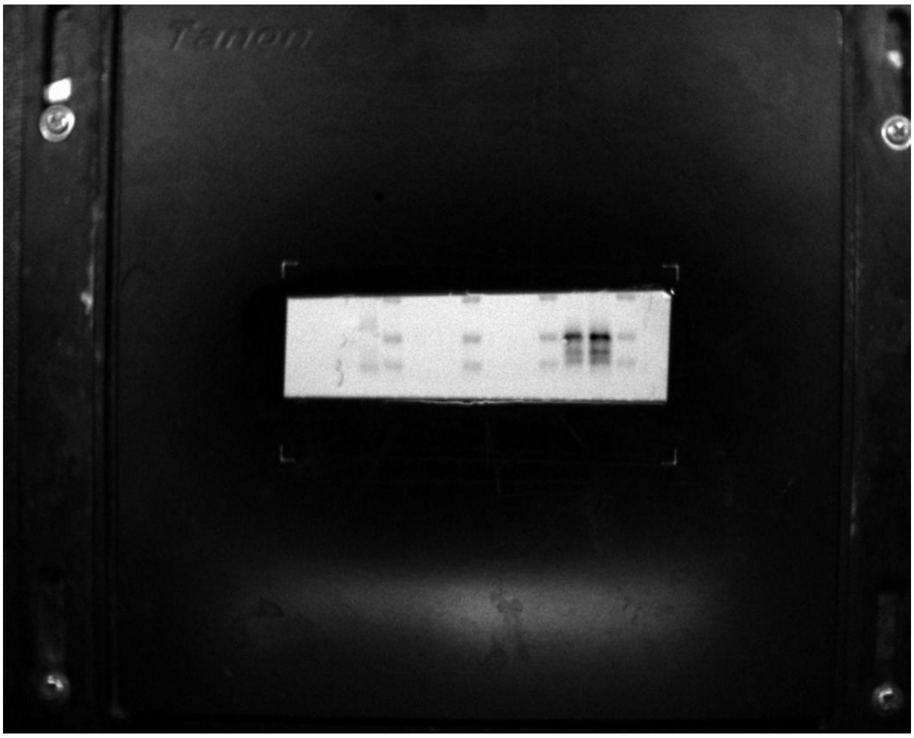

VDAC1 for Fig. 3C.

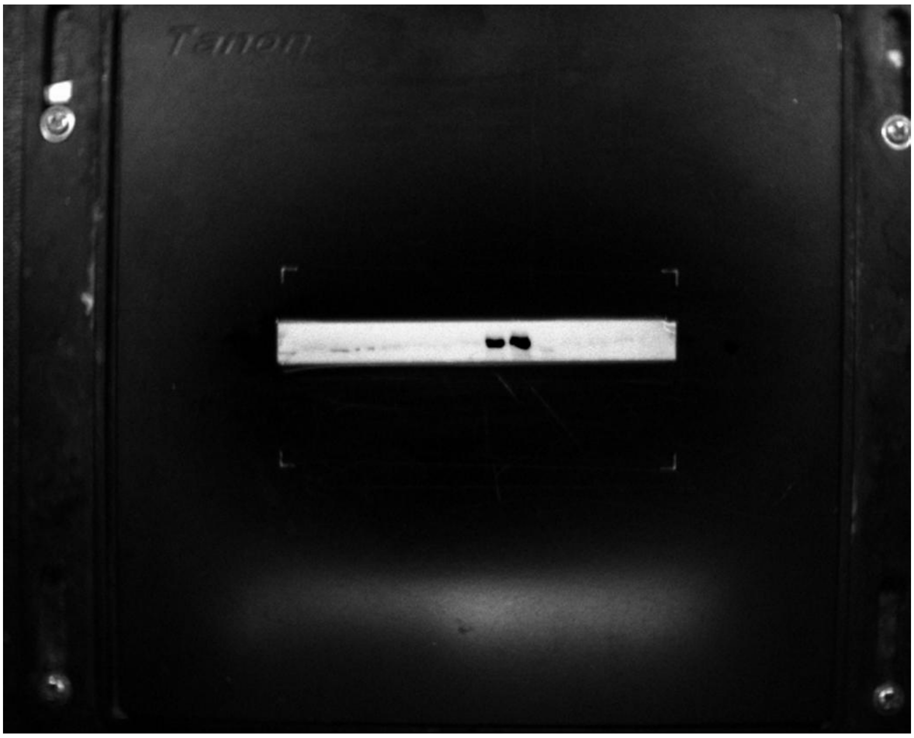

IP3R1 for Fig. 3C.

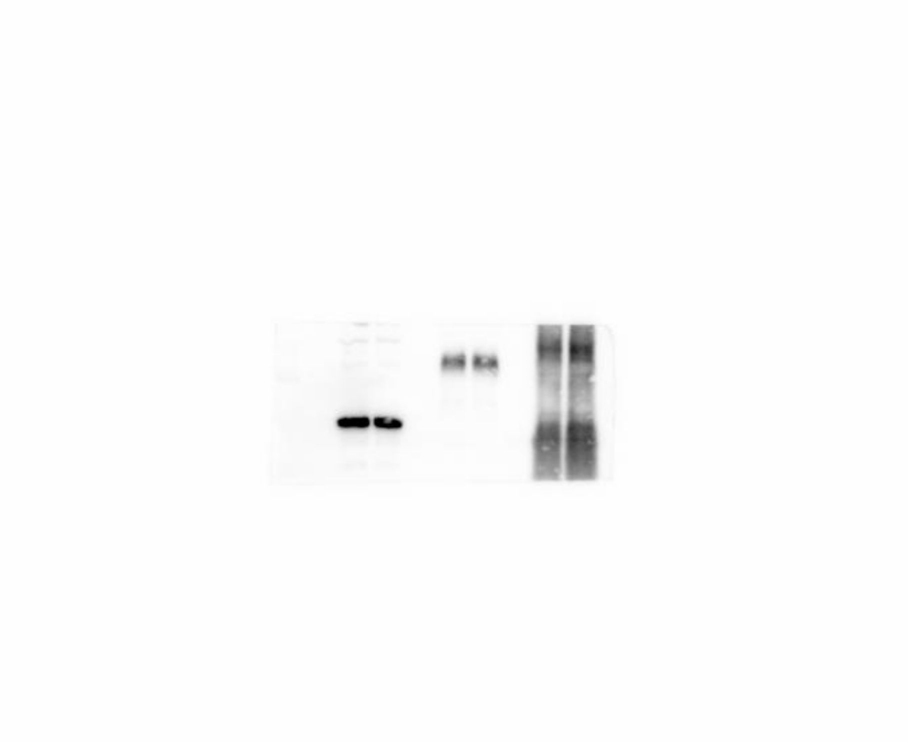

GAPDH for Fig. 3C.

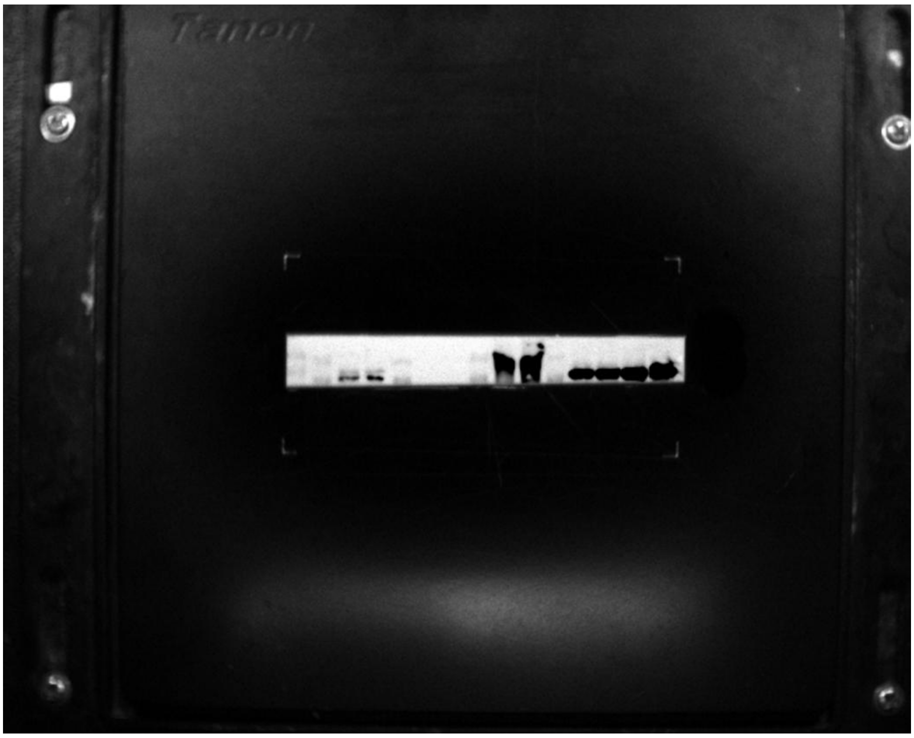

GRP75 for Fig. 3C.

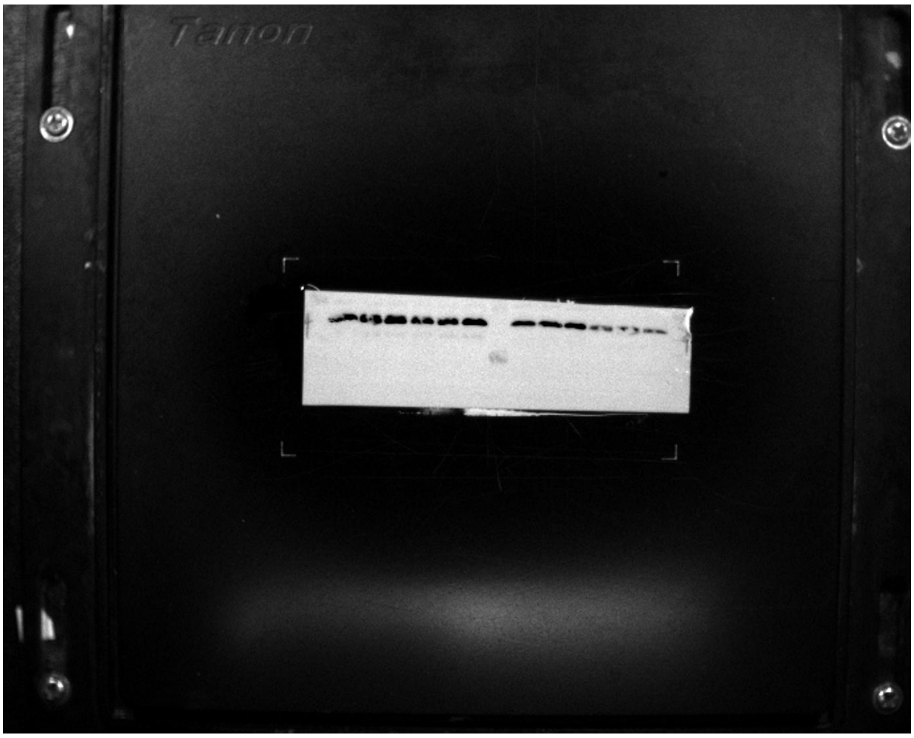

BAX for Fig. 3E.

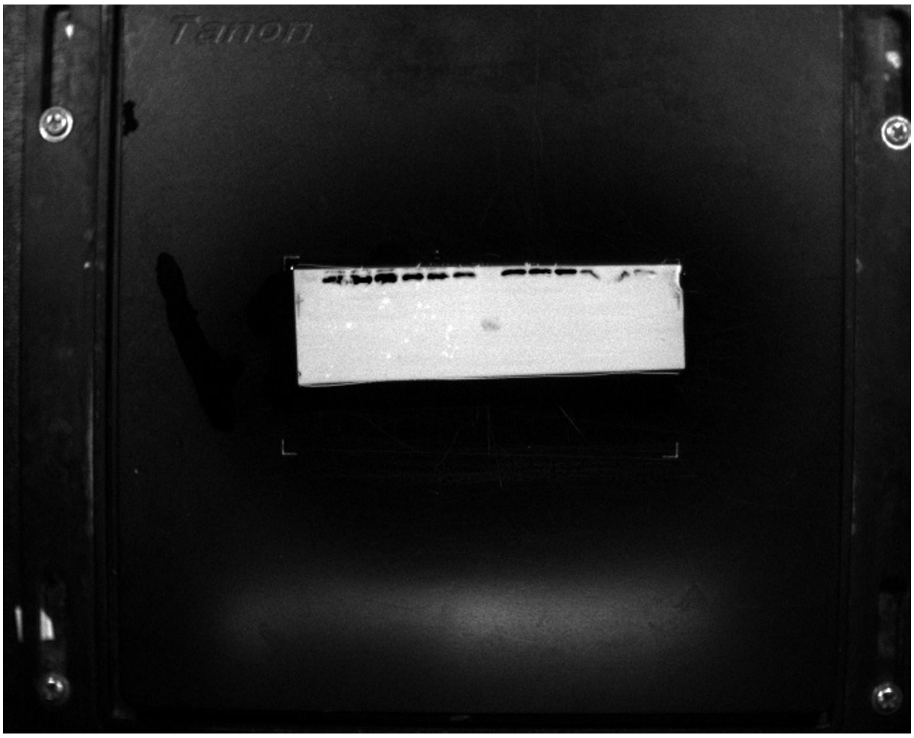

Bcl-2 for Fig. 3E.

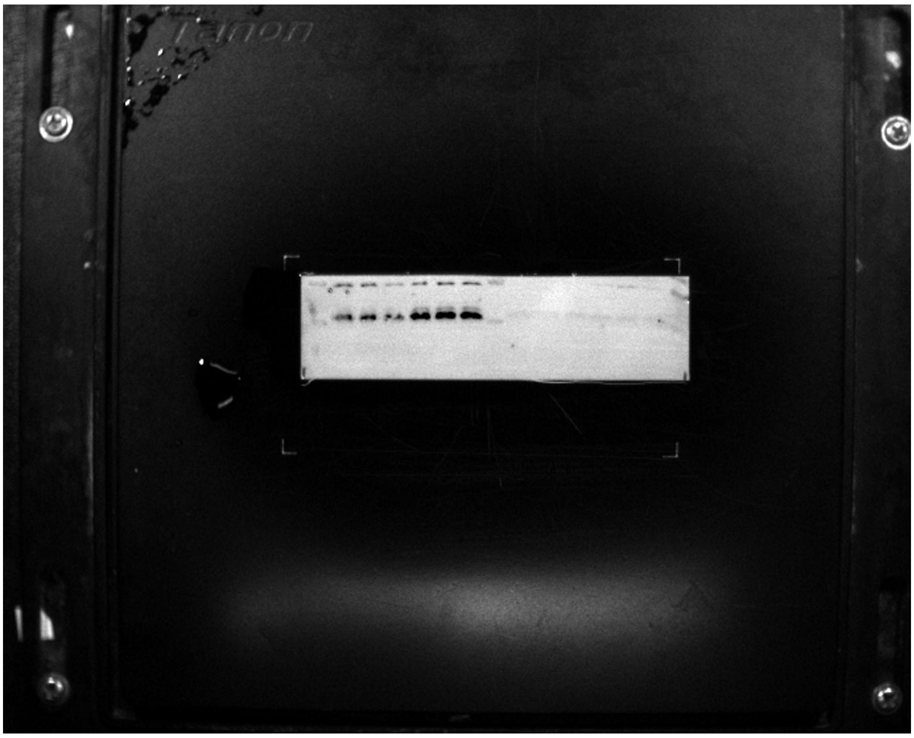

Caspase3 and Cleaved-caspase3 for Fig. 3E.

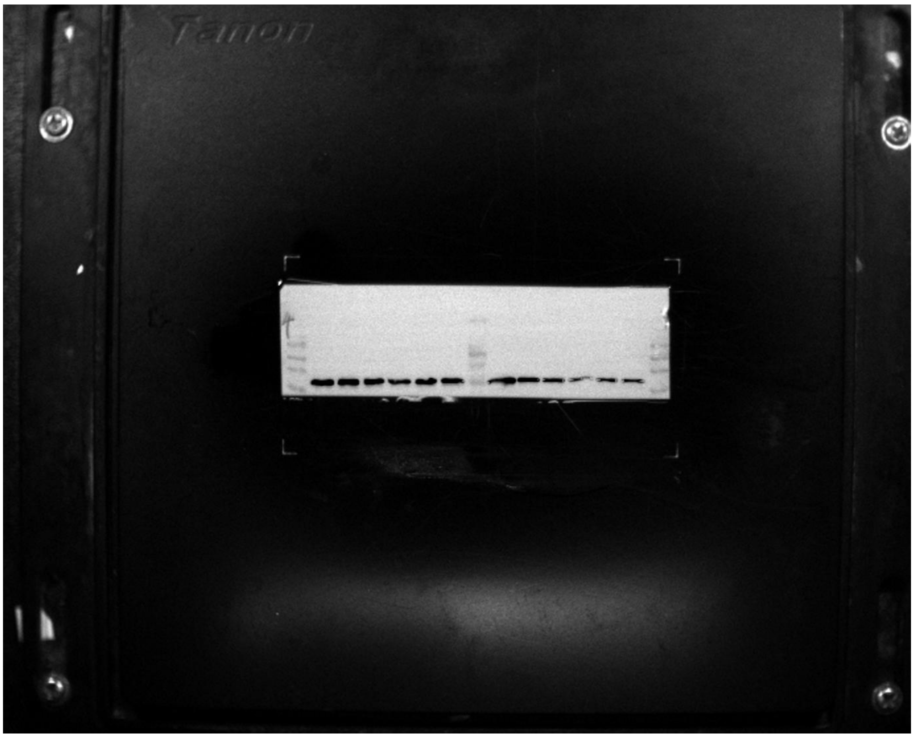

GAPDH for Fig. 3E.

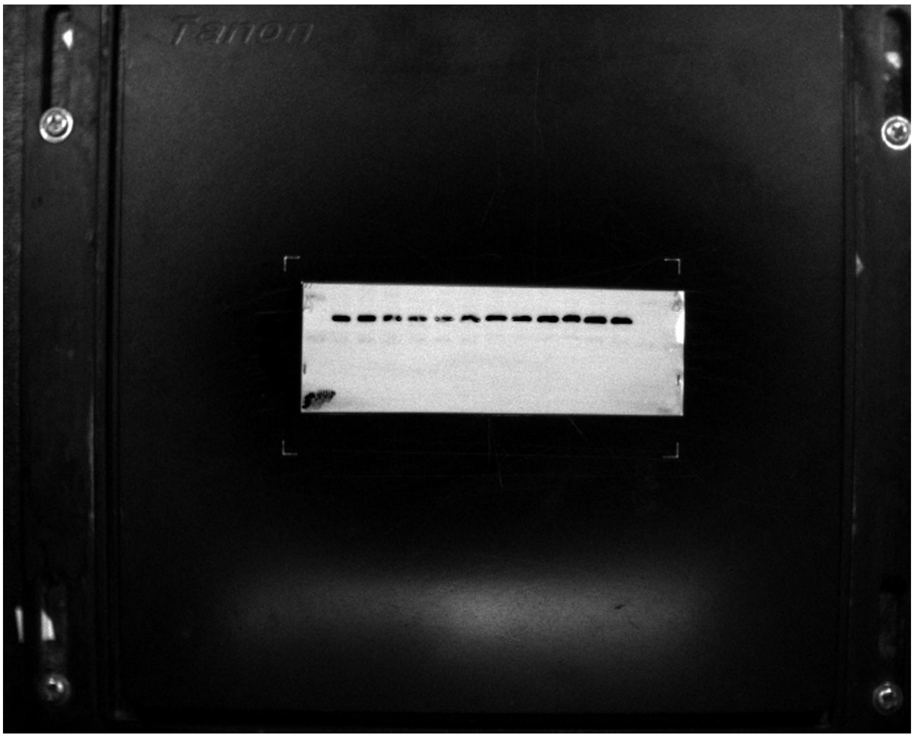

BAX for Fig. 3O.

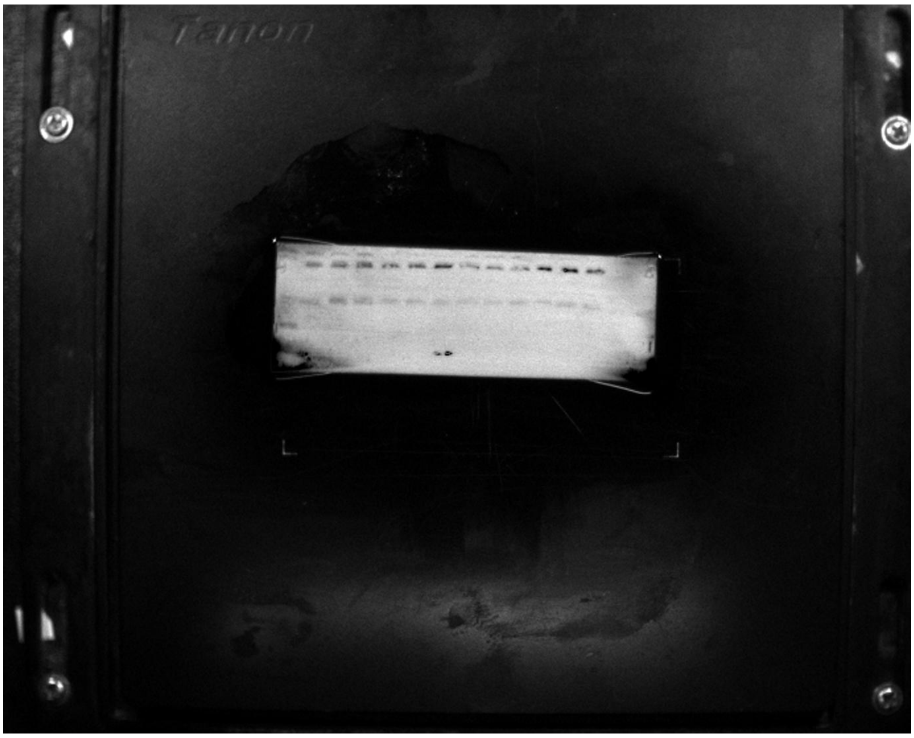

Bcl-2 for Fig. 3O.

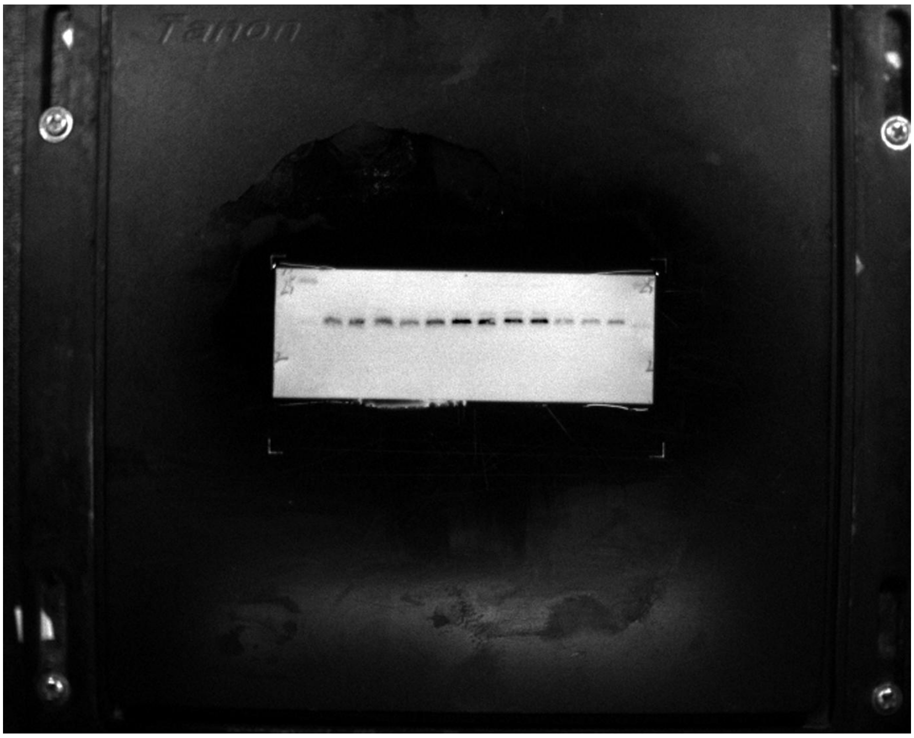

Cleaved-caspase3 for Fig. 3O.

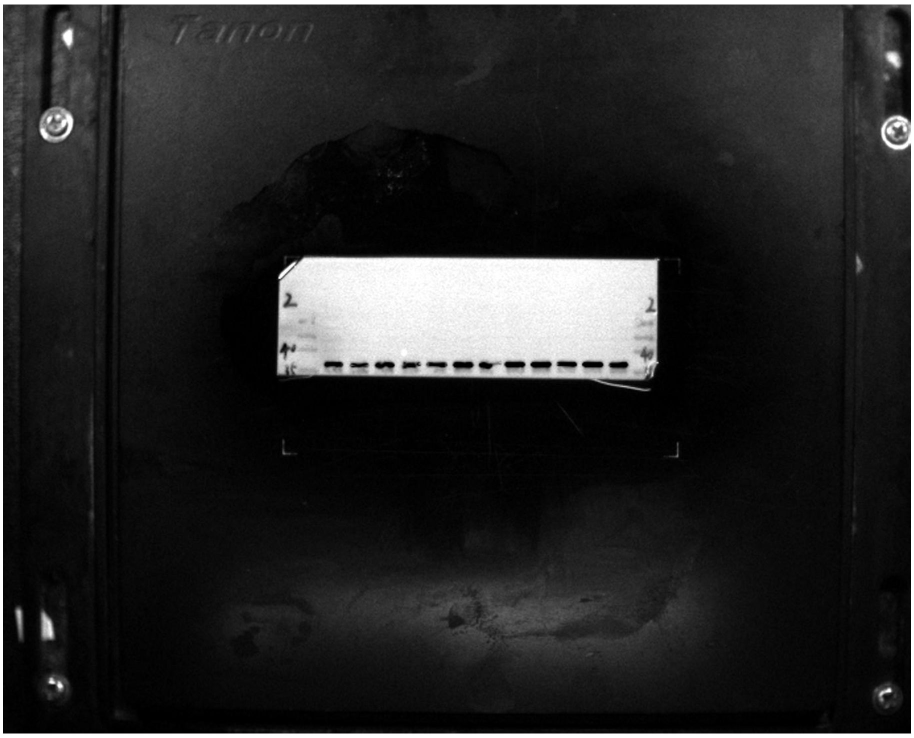

GAPDH for Fig. 3O.

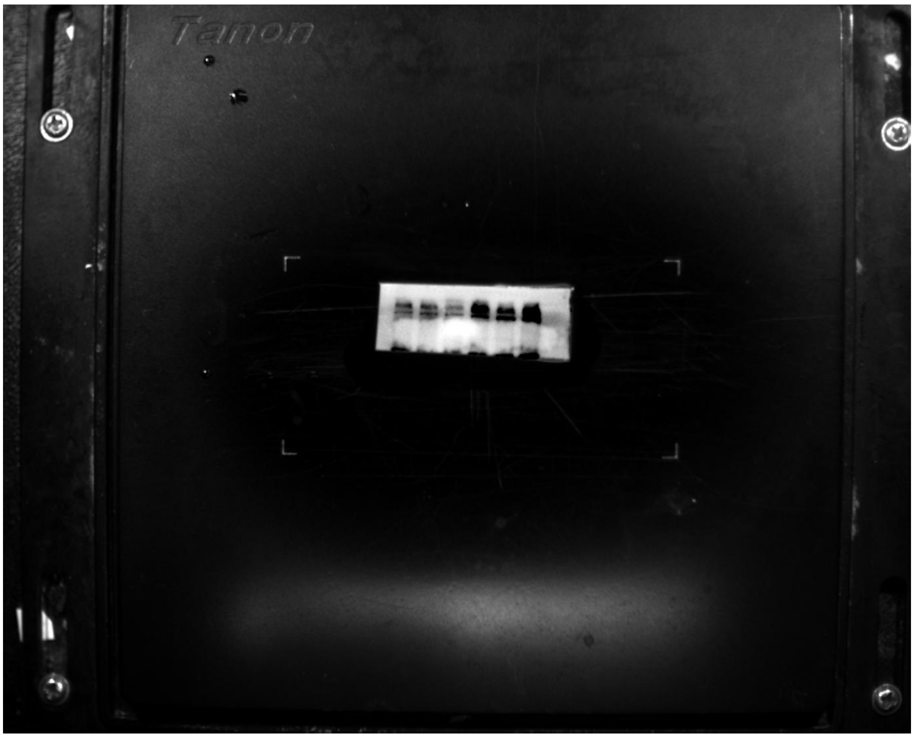

IP3R1 for Fig. 4C.

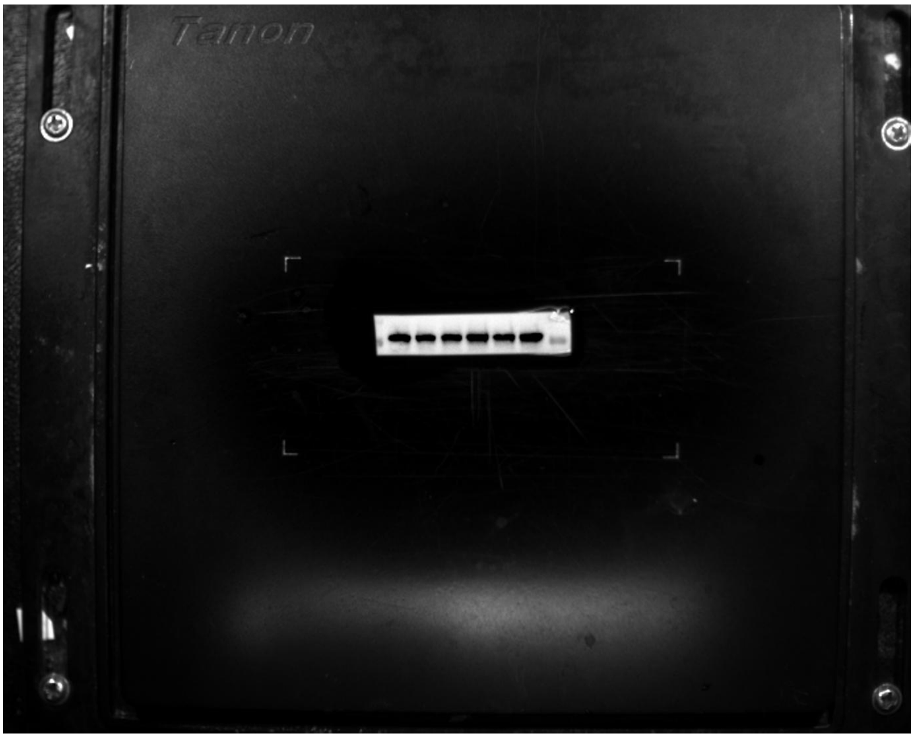

Grp75 for Fig. 4C.

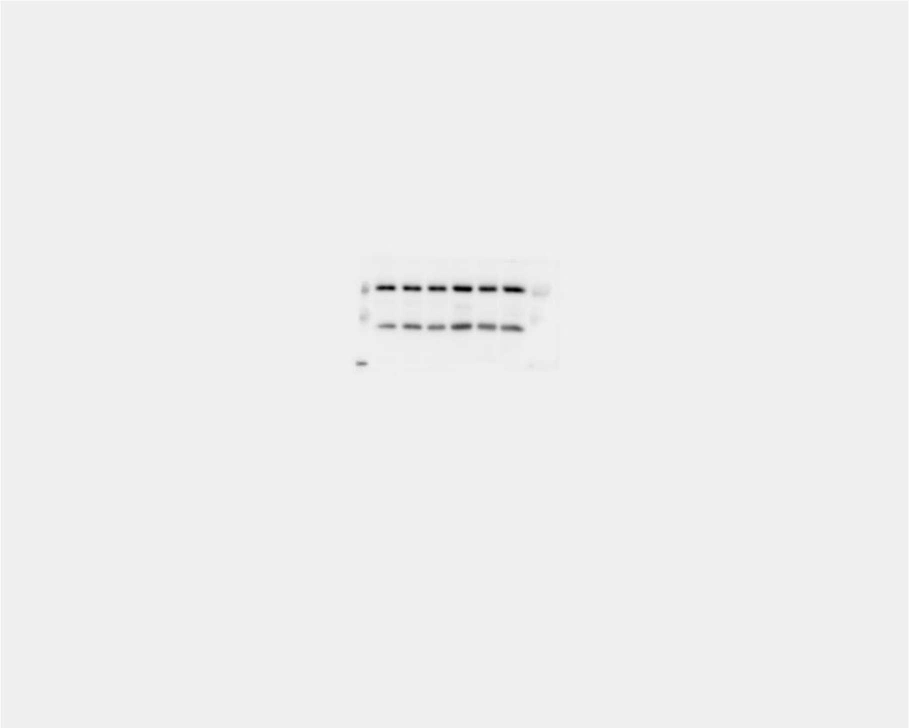

VDAC1 for Fig. 4C.

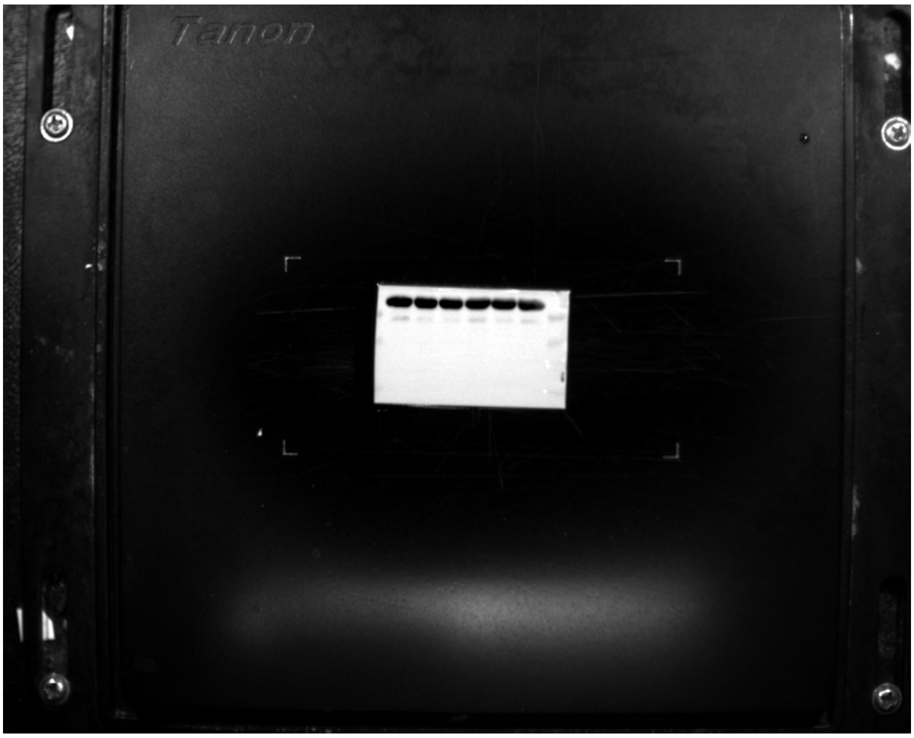

GAPDH for Fig. 4C.

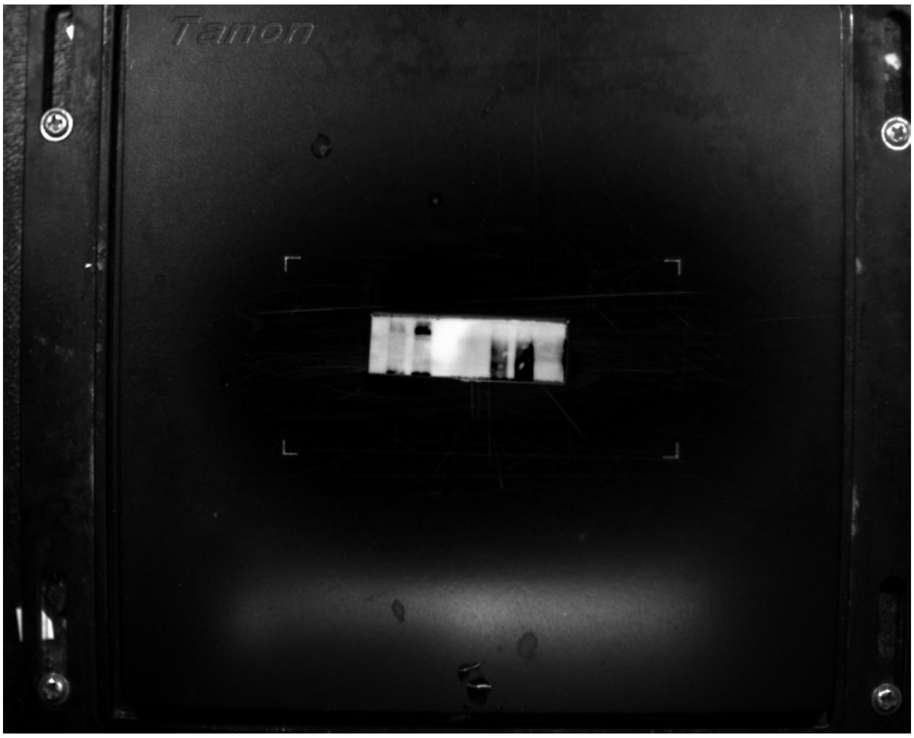

IP3R1 for Fig. 4G.

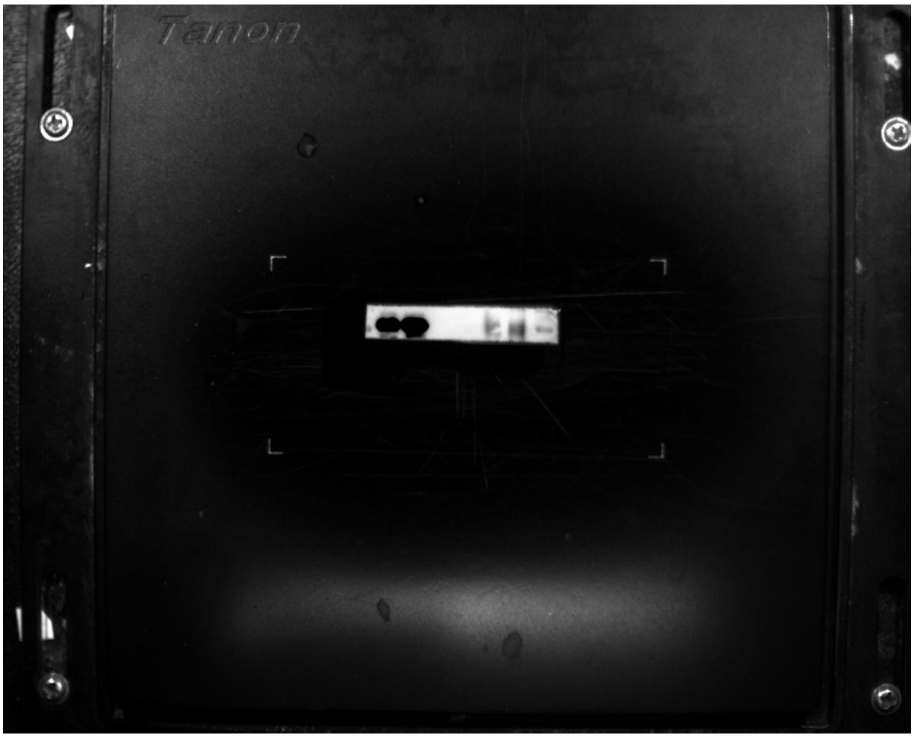

GRP75 for Fig. 4G.

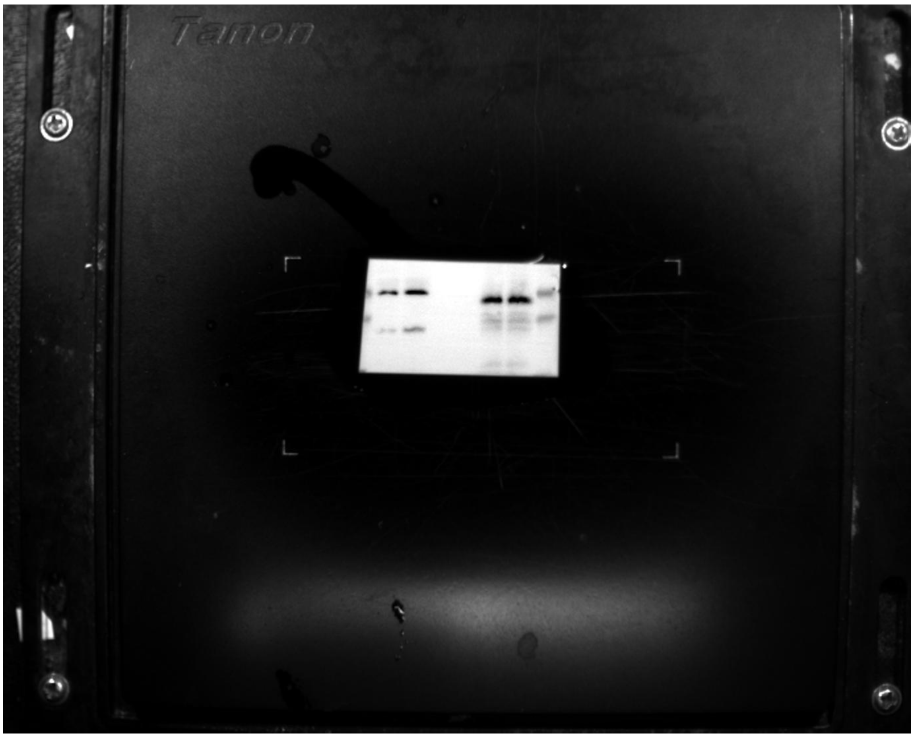

VDAC1 for Fig. 4G.

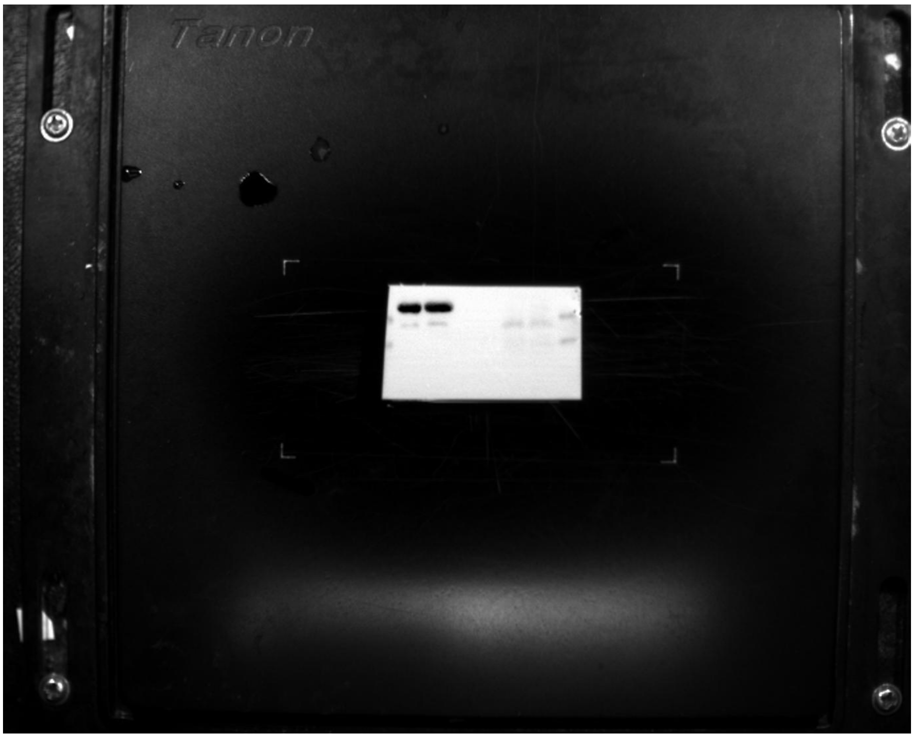

GAPDH for Fig. 4G.

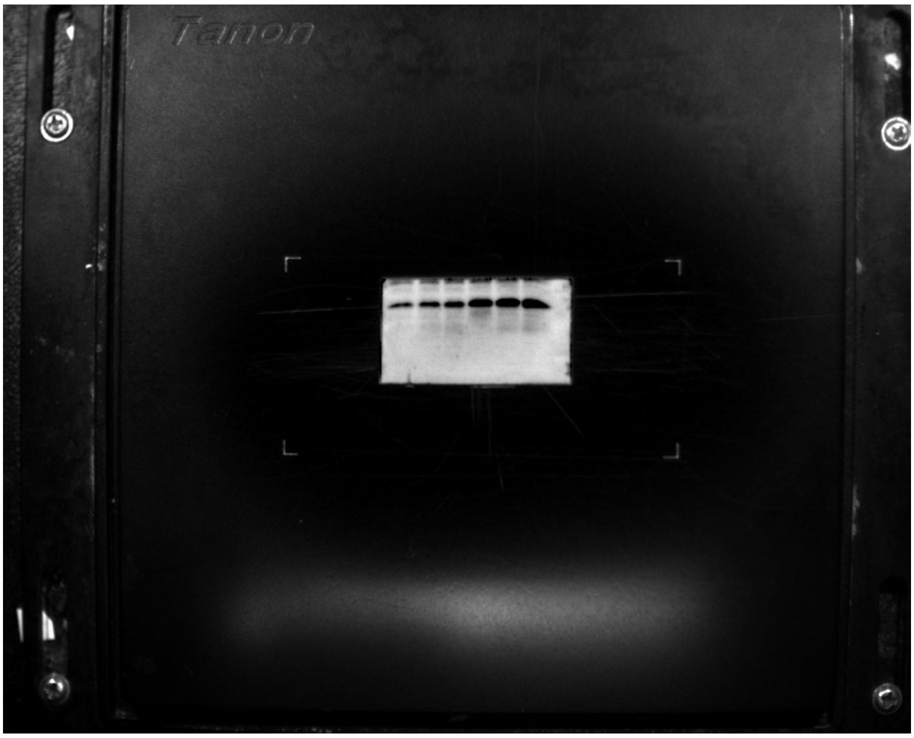

BAX for Fig. 4M.

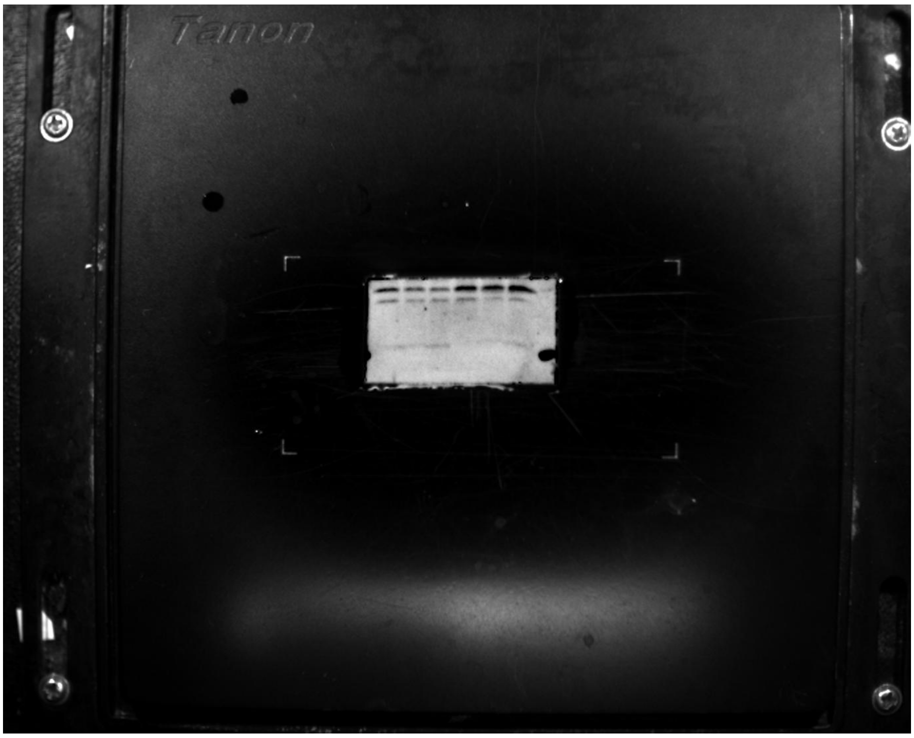

BCL-2 for Fig. 4M.

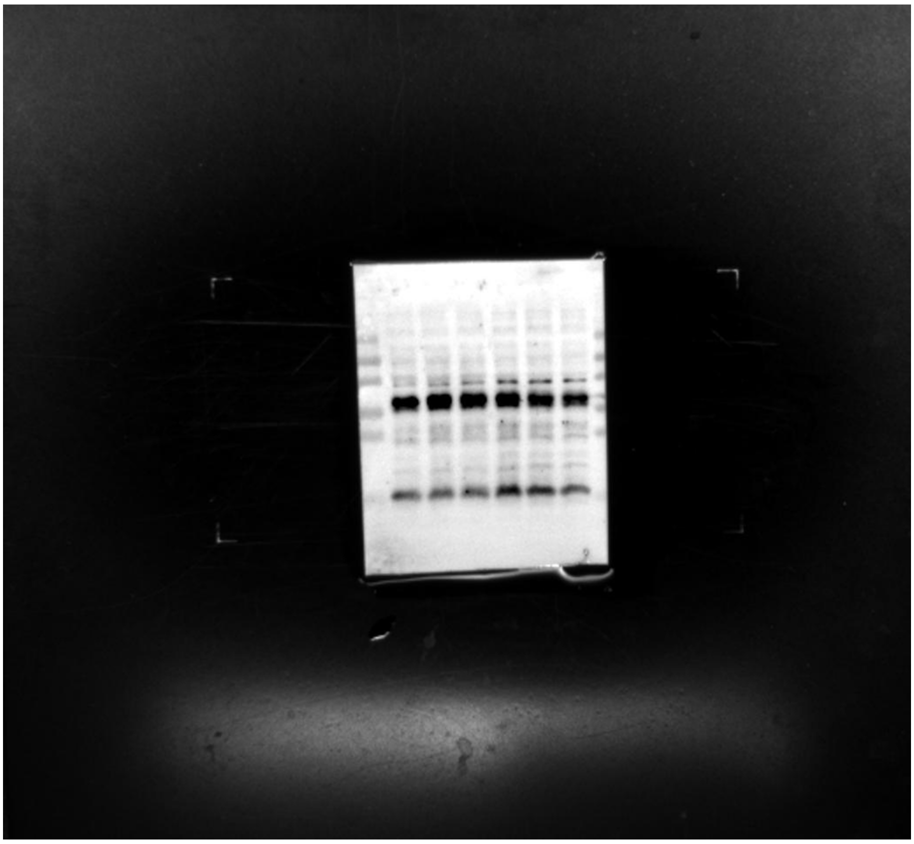

caspase3 and Cleaved-caspase3 for Fig. 4M.

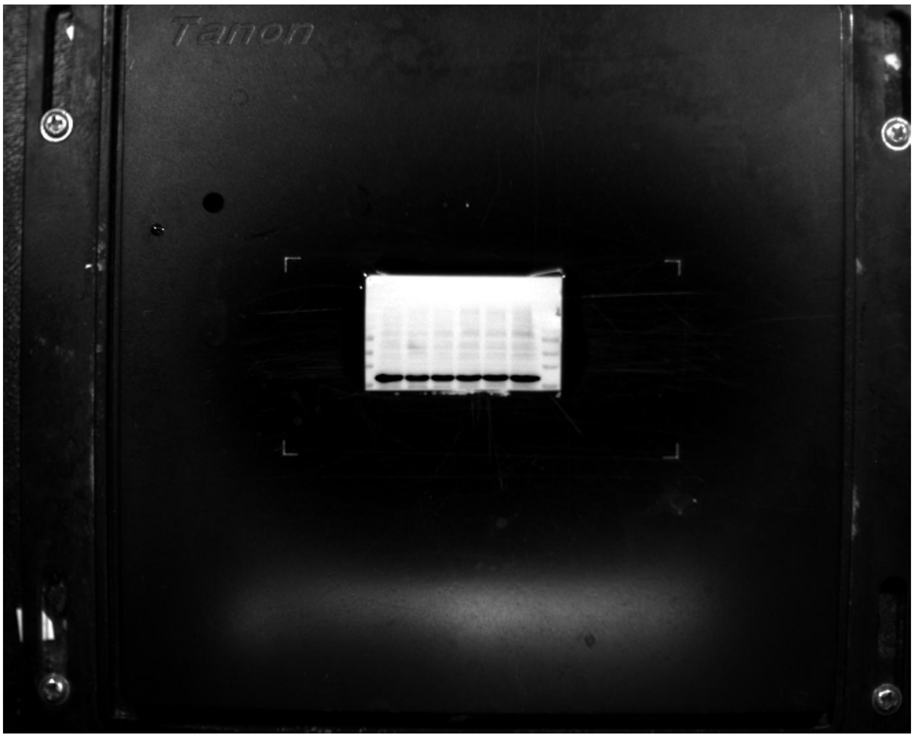

GAPDH for Fig. 4M.

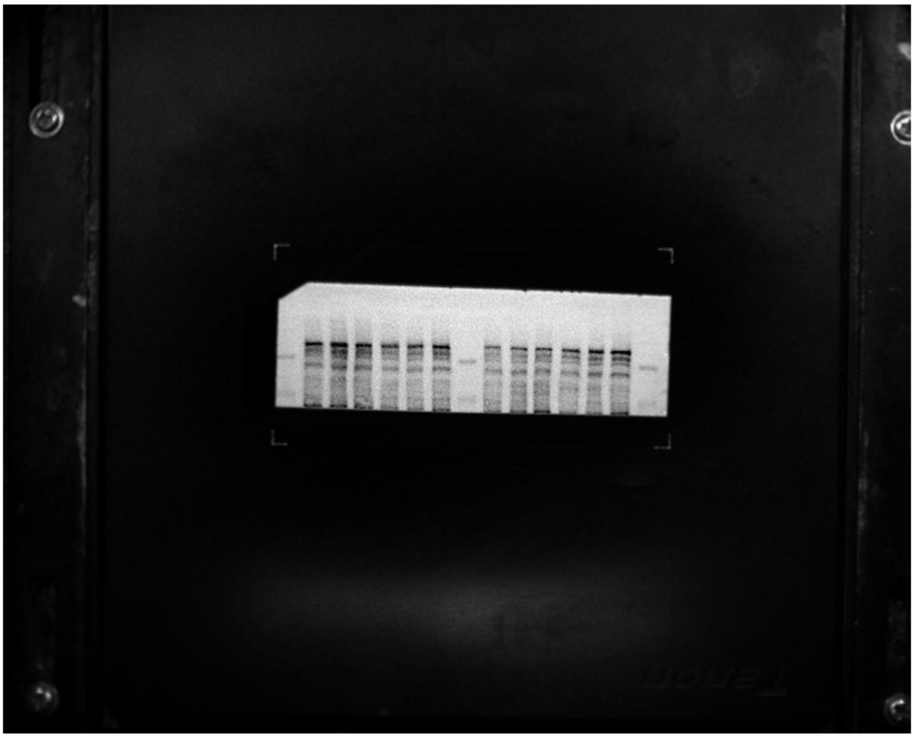

IP3R1 for Fig. 5A.

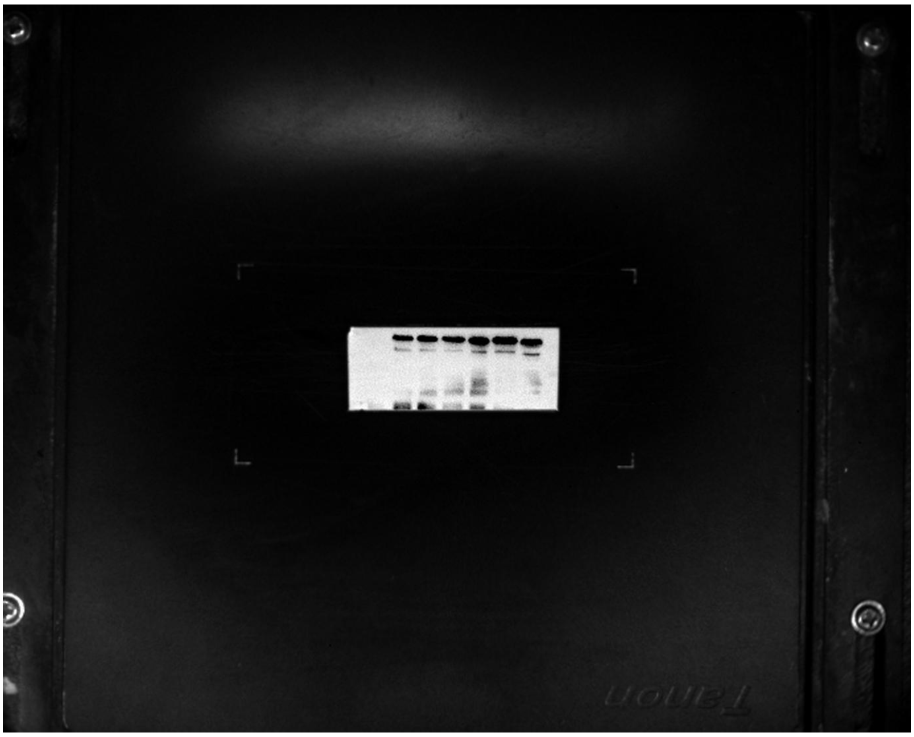

GRP75 for Fig. 5A.

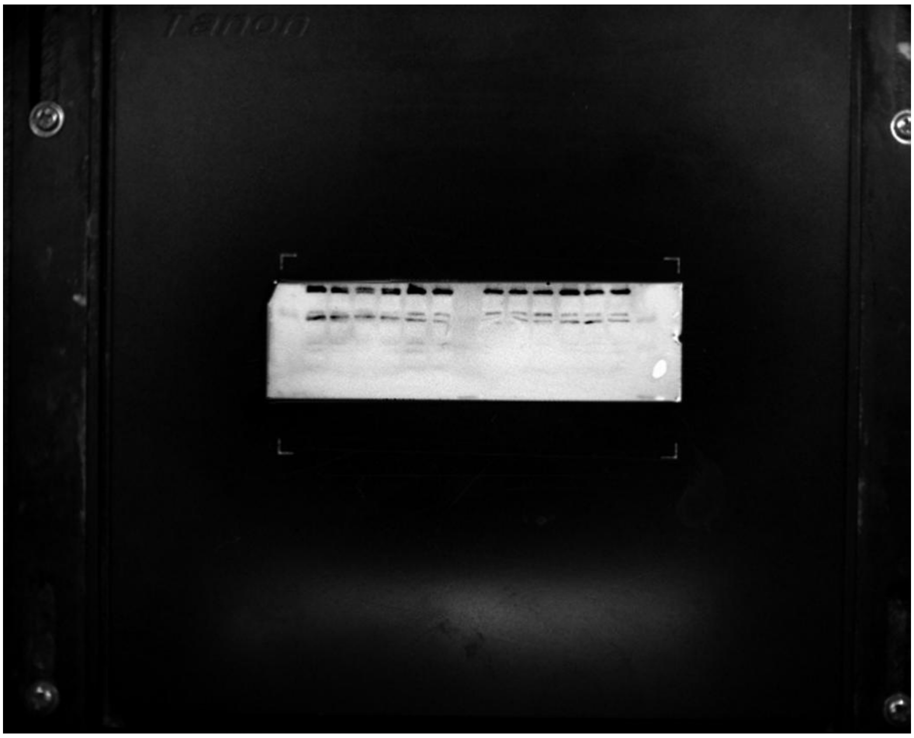

VDAC1 for Fig. 5A.

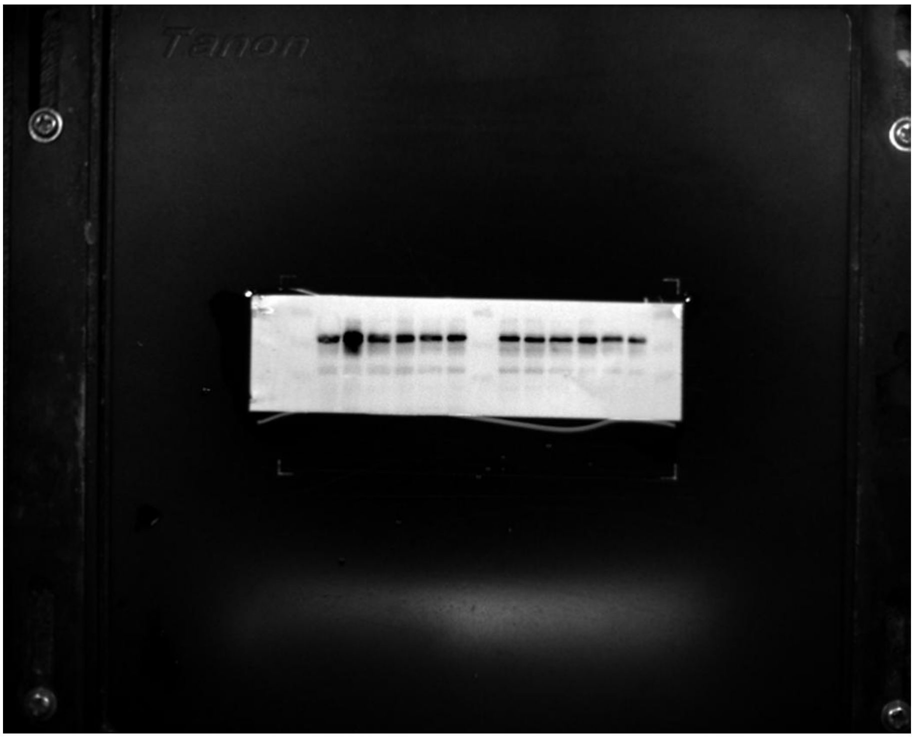

GAPDH for Fig. 5A.

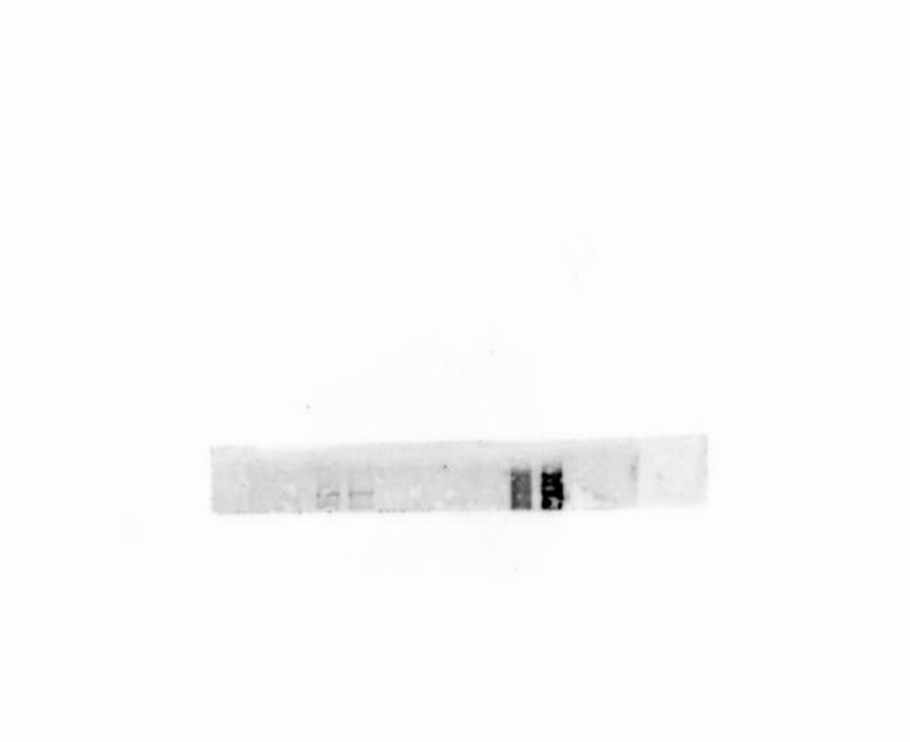

IP3R1 for Fig. 5C.

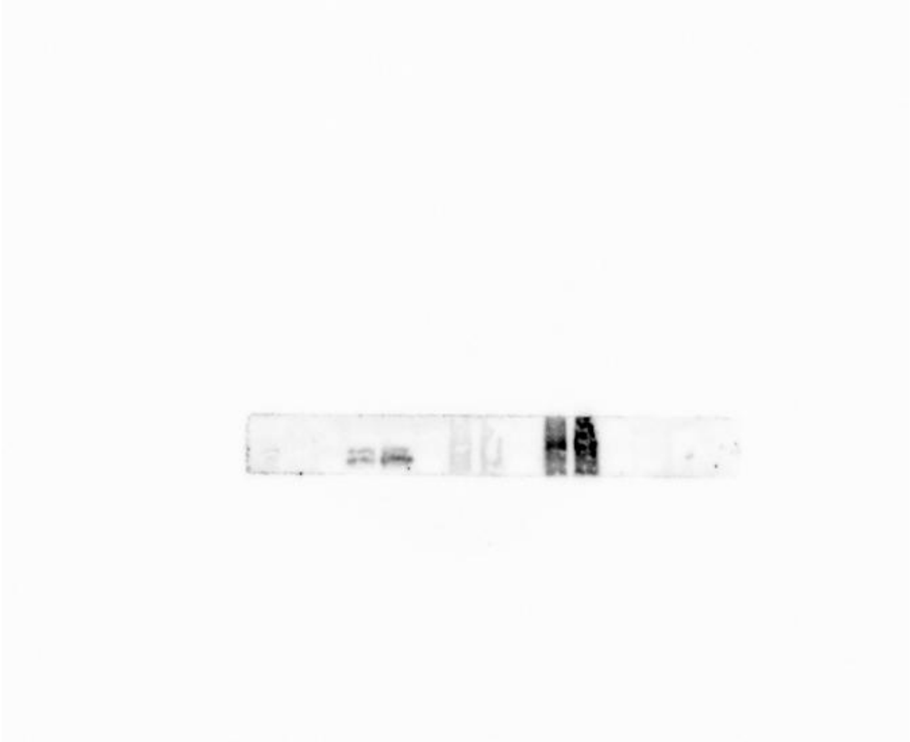

GRP75 for Fig. 5C.

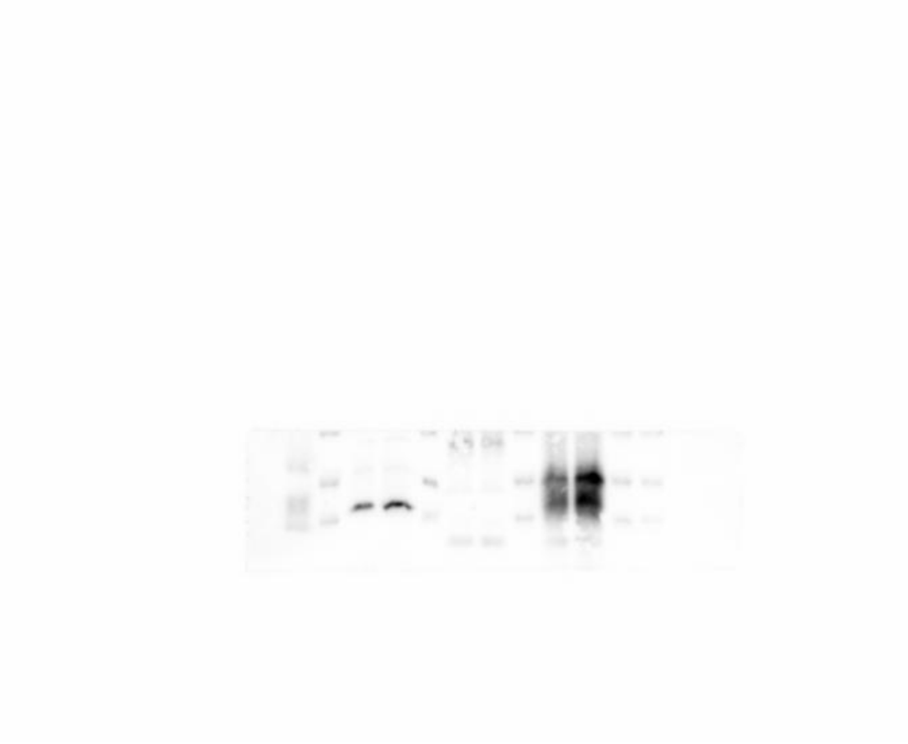

VDAC1 for Fig. 5C.

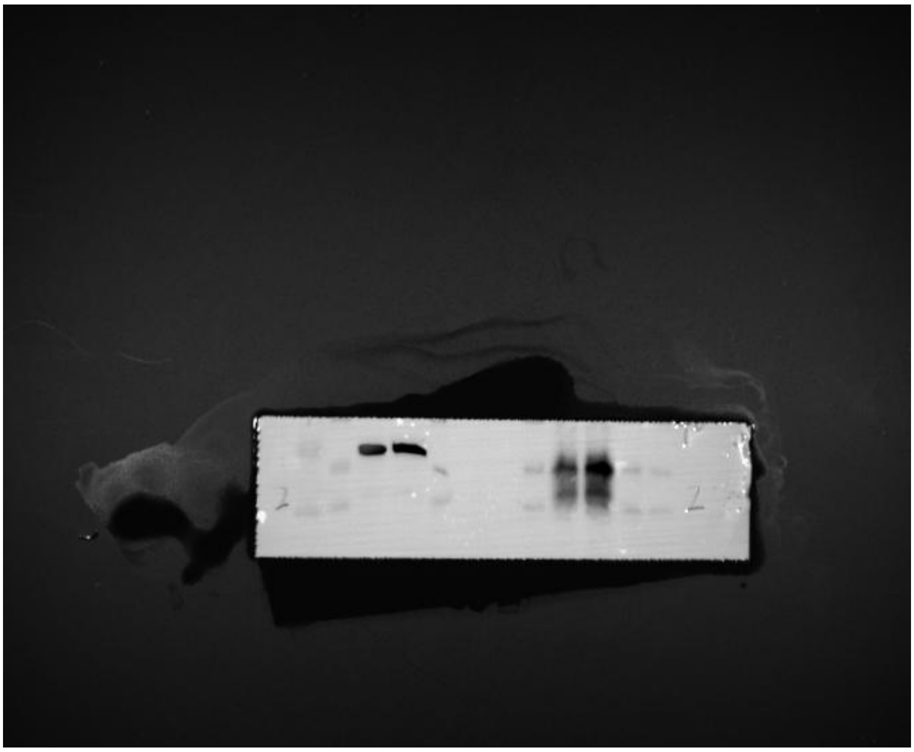

GAPDH for Fig. 5C.

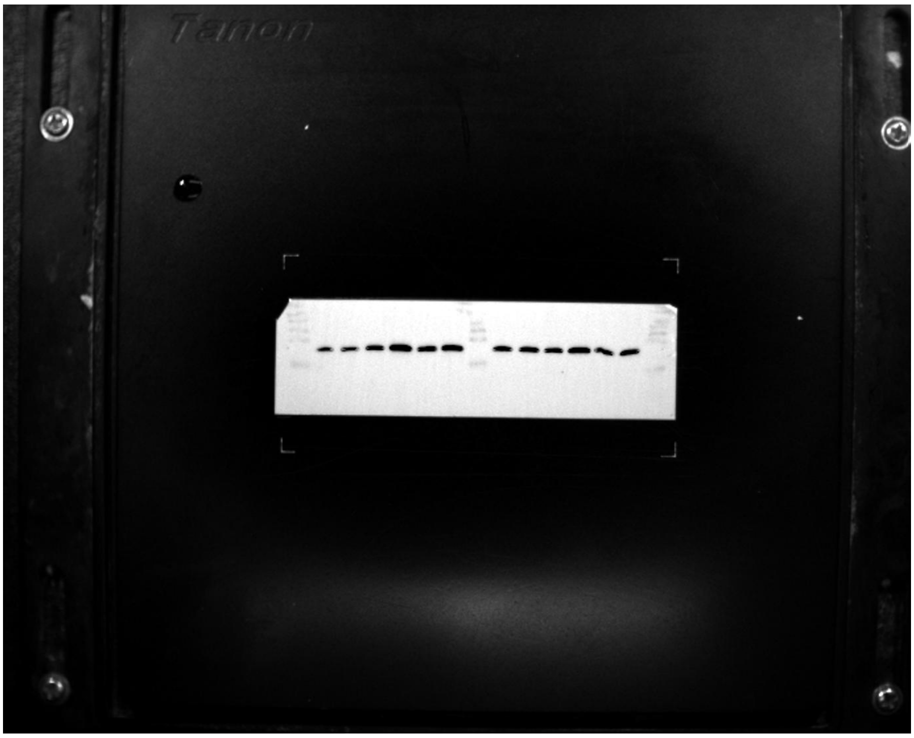

BAX for Fig. 5Q.

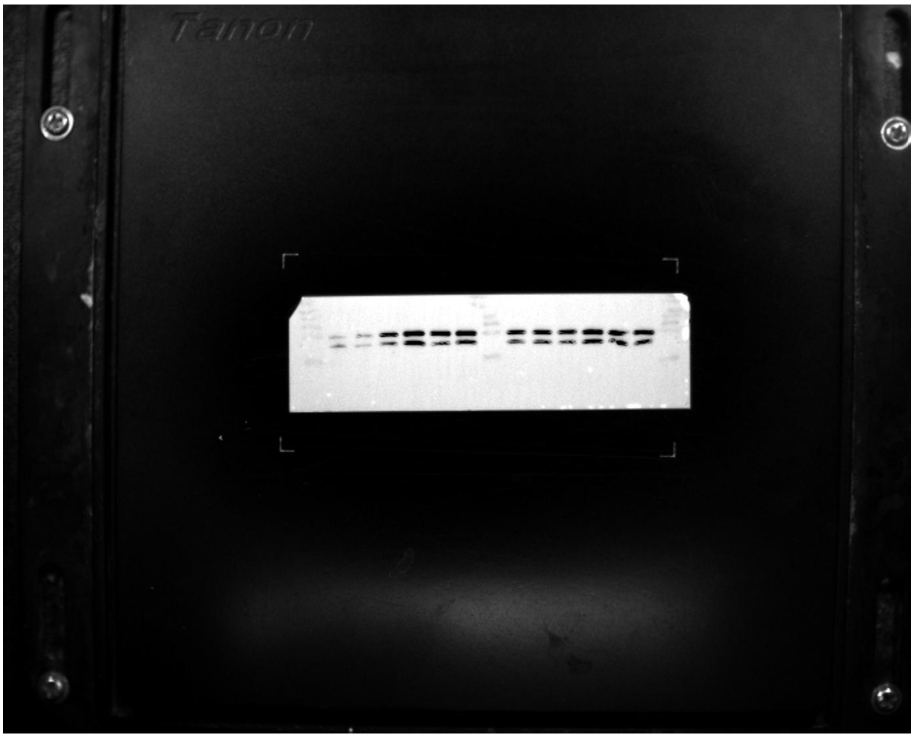

BCL-2 for Fig. 5Q.

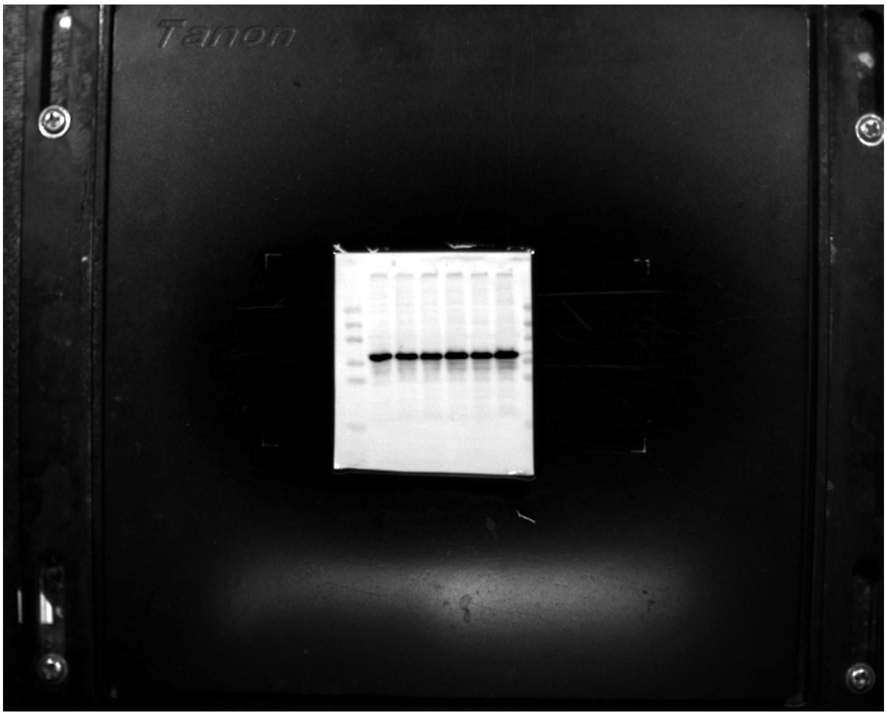

caspase3 for Fig. 5Q.

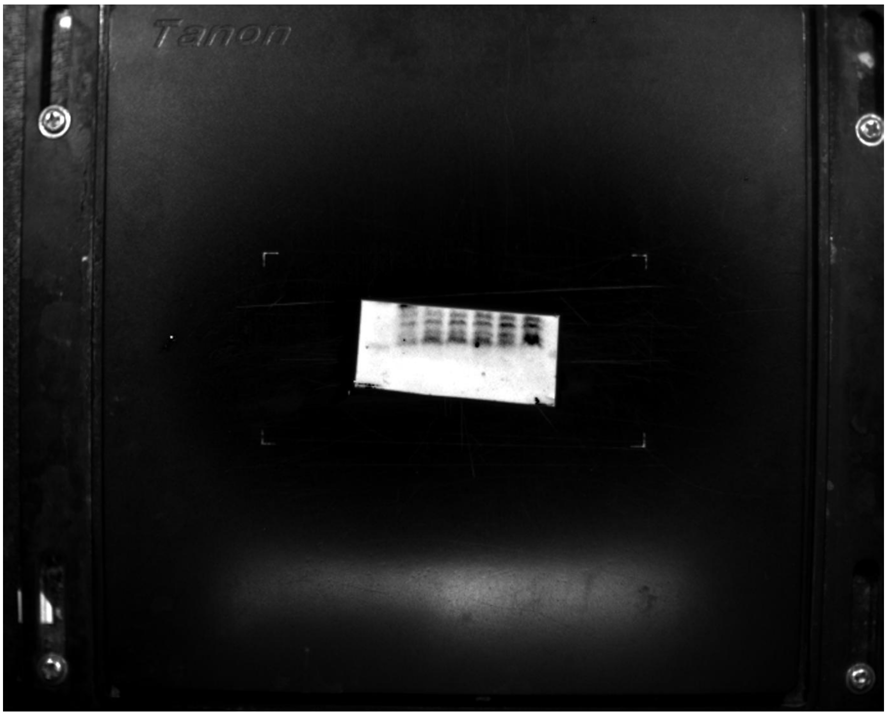

Cleaved-caspase3 for Fig. 5Q.

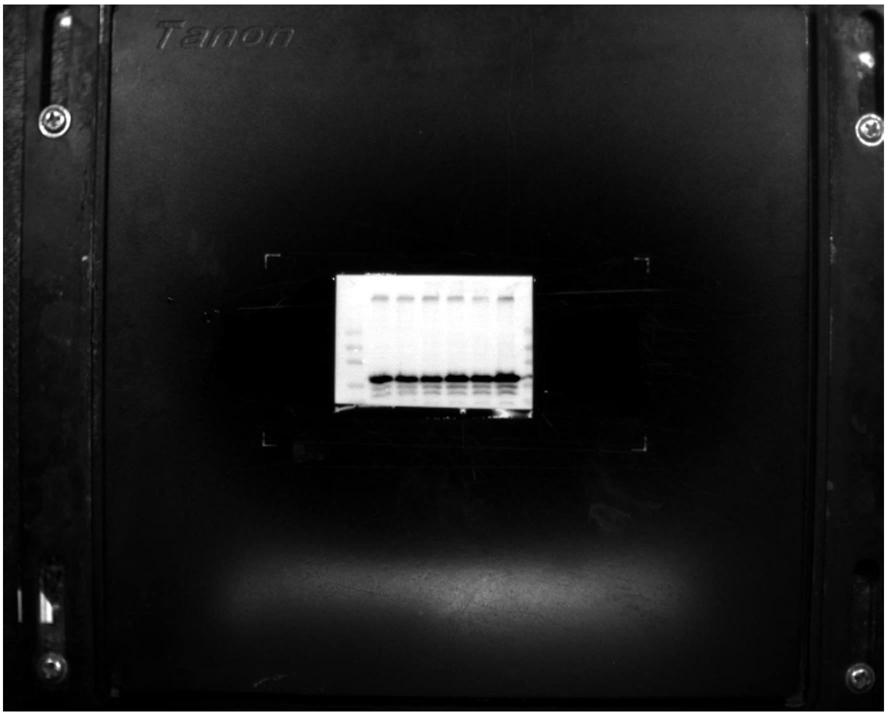

GAPDH for Fig. 5Q.

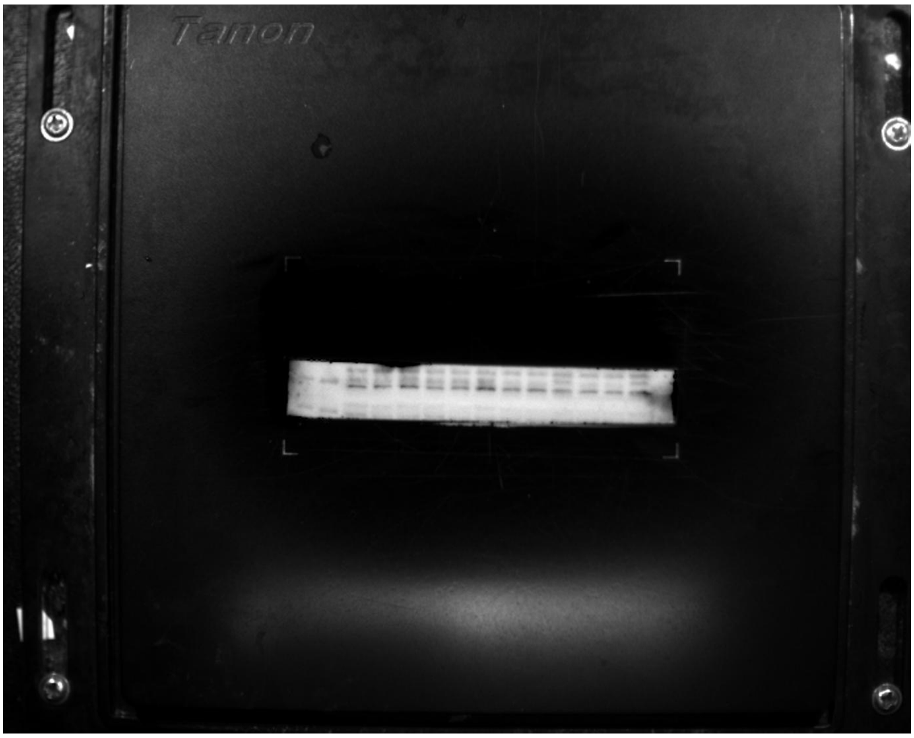

TRα for Fig. 6C.

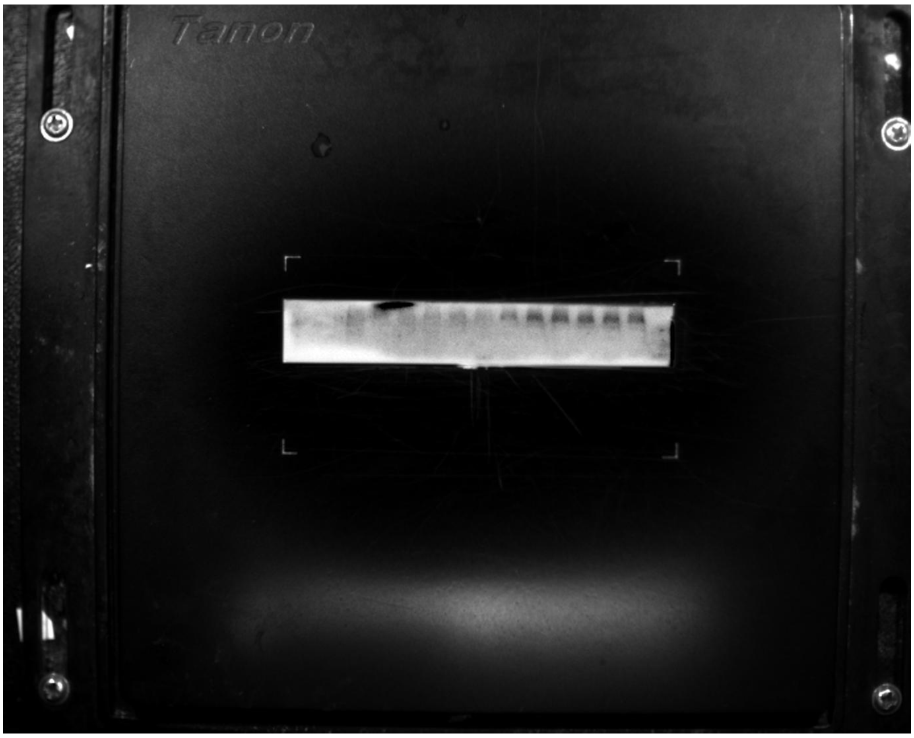

IP3R1 for Fig. 6C.

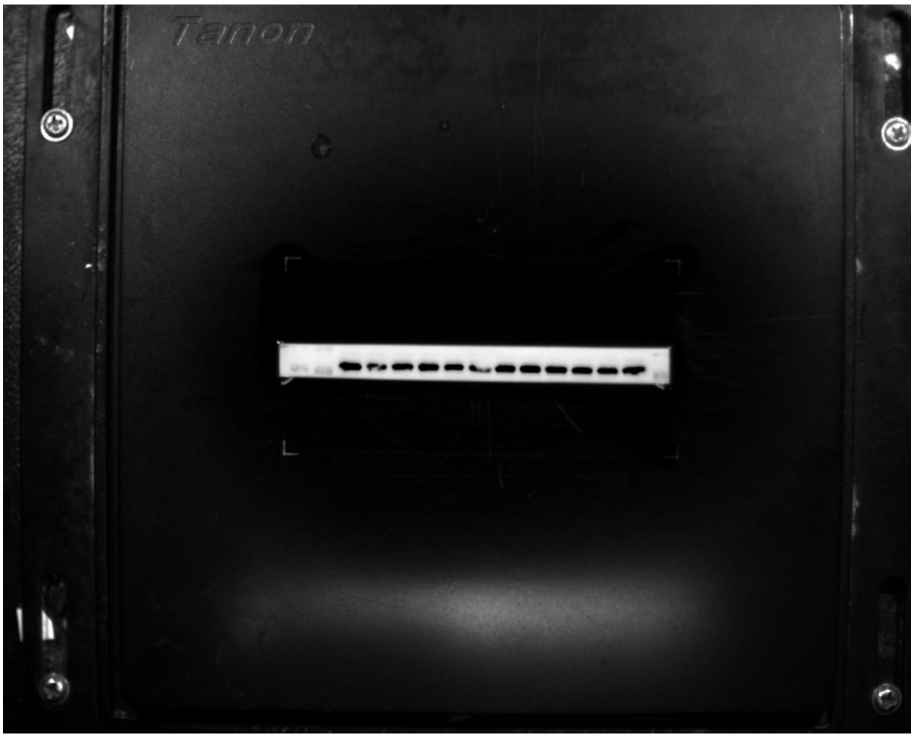

Grp75 for Fig. 6C.

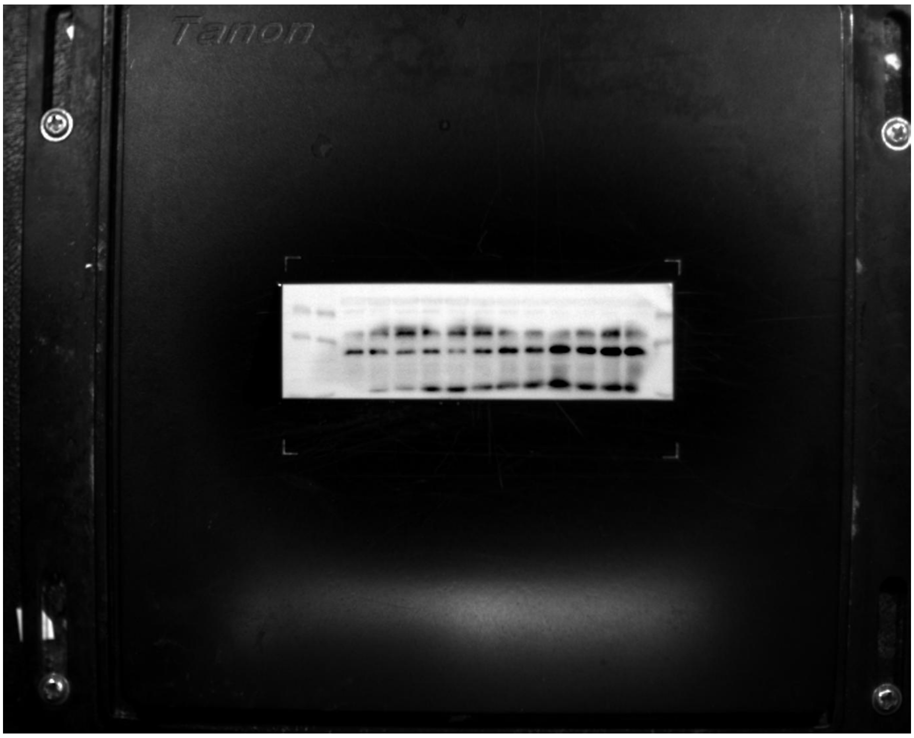

VDAC1 for Fig. 6C.

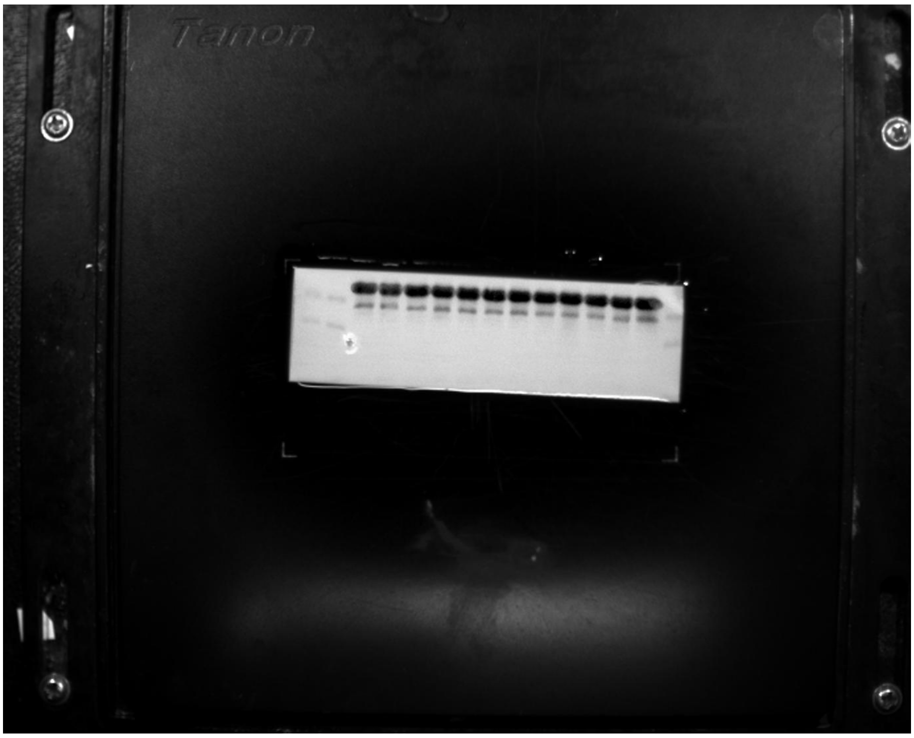

GAPDH for Fig. 6C.

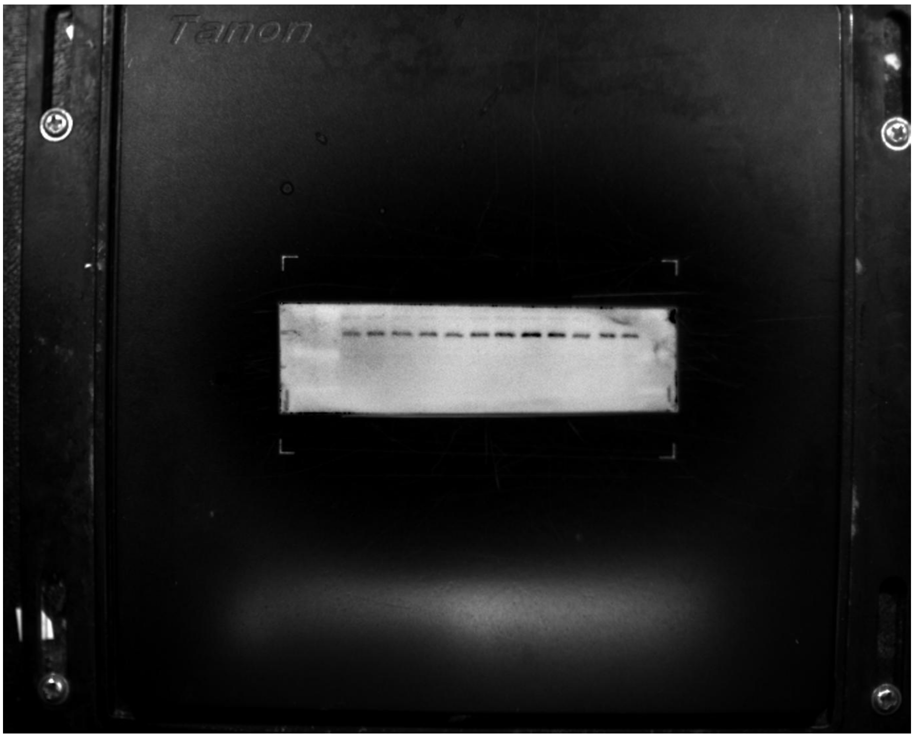

BAX for Fig. 6K.

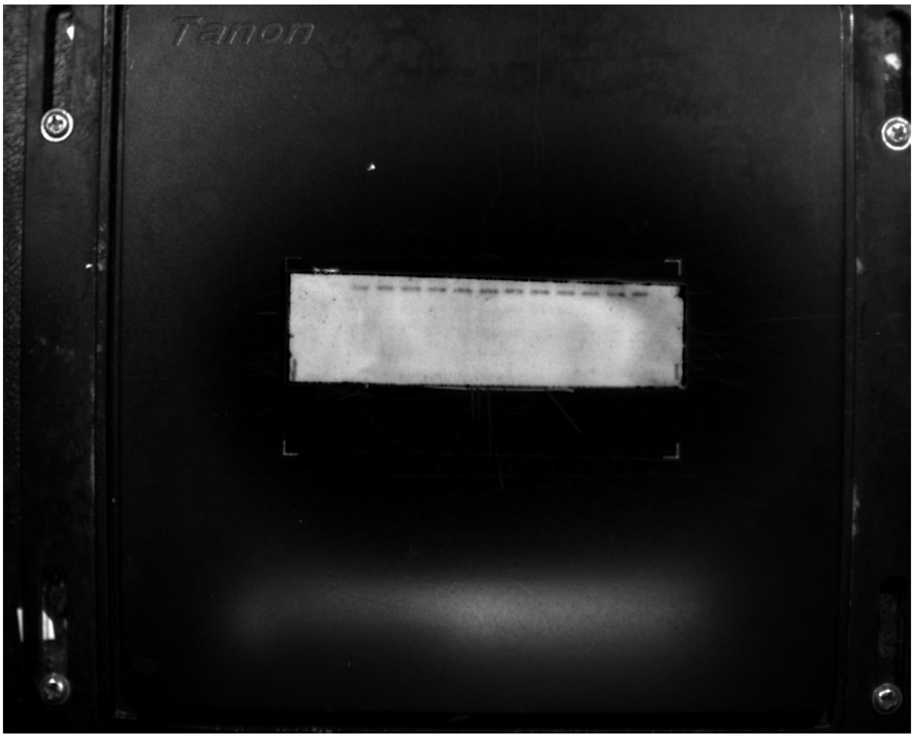

Bcl-2 for Fig. 6K.

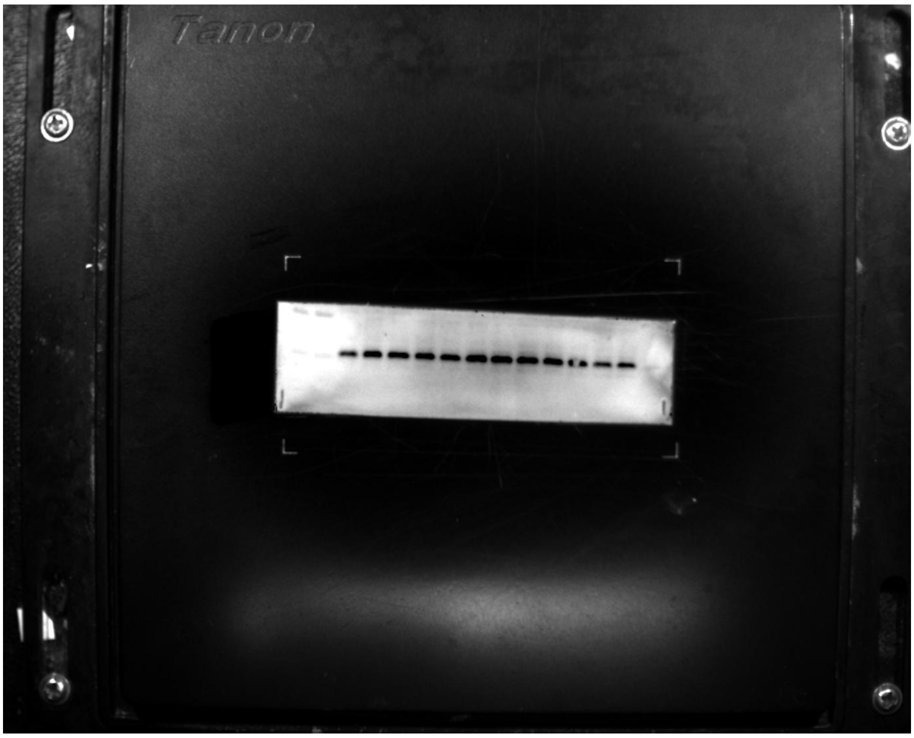

Cleaved-caspase3 for Fig. 6K.

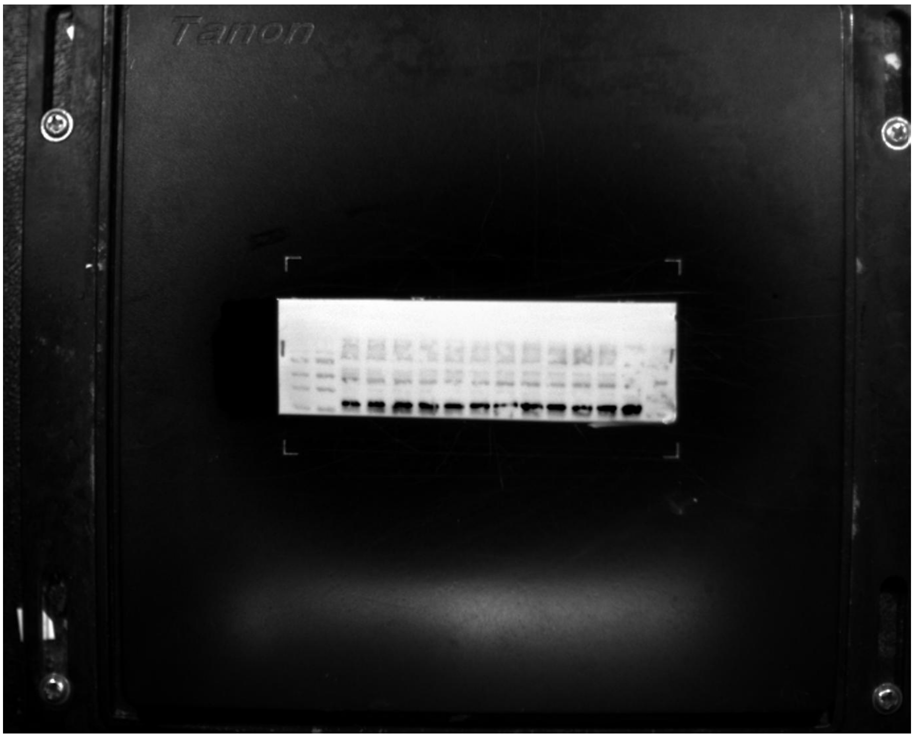

GAPDH for Fig. 6K.

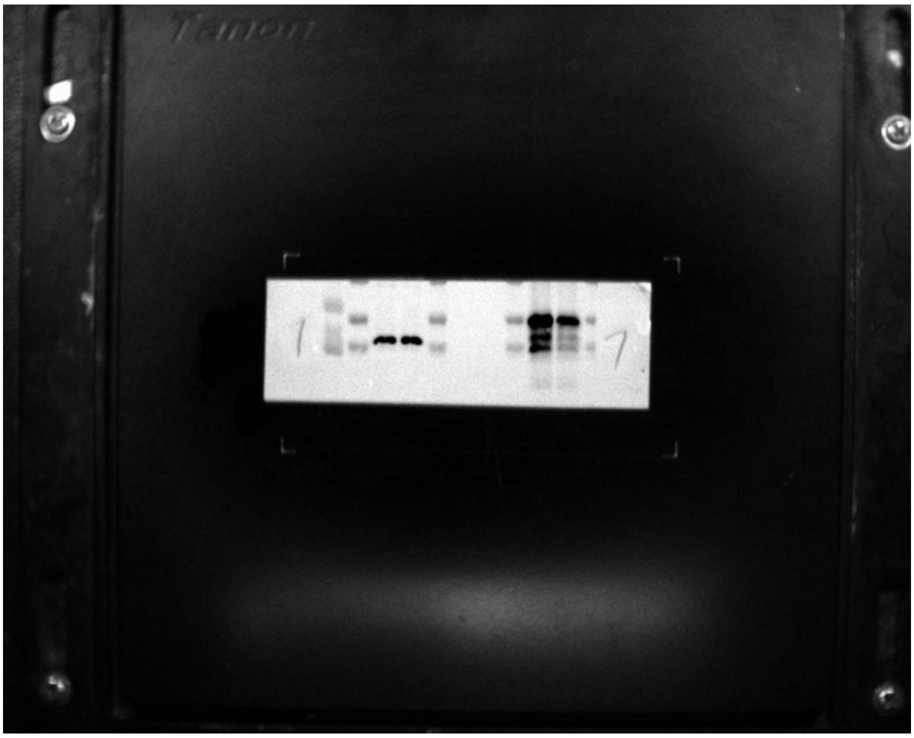

VDAC1 for Fig. 7A.

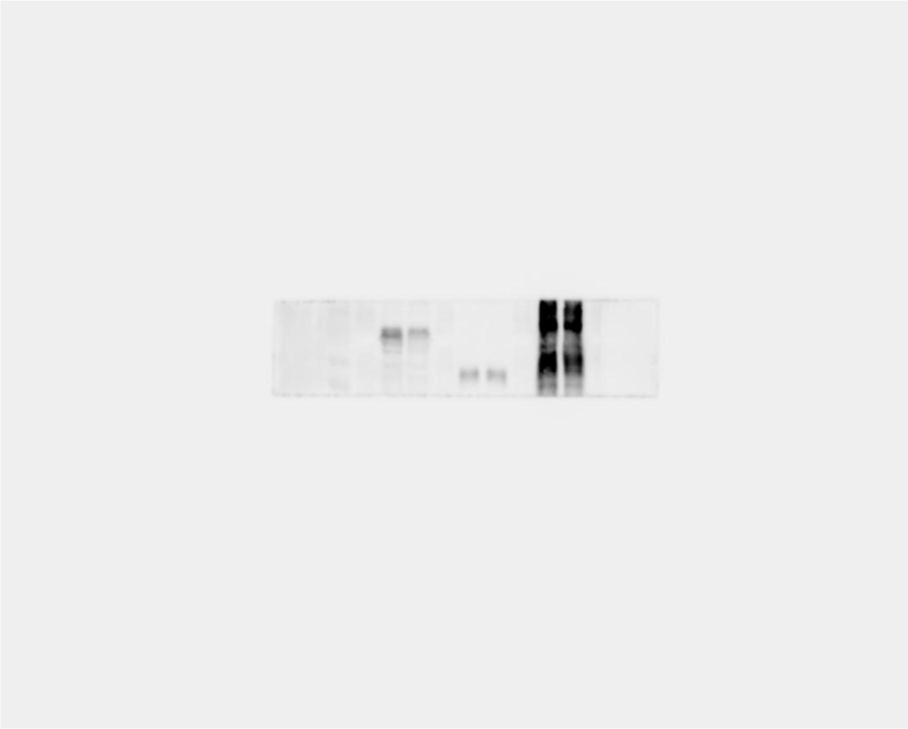

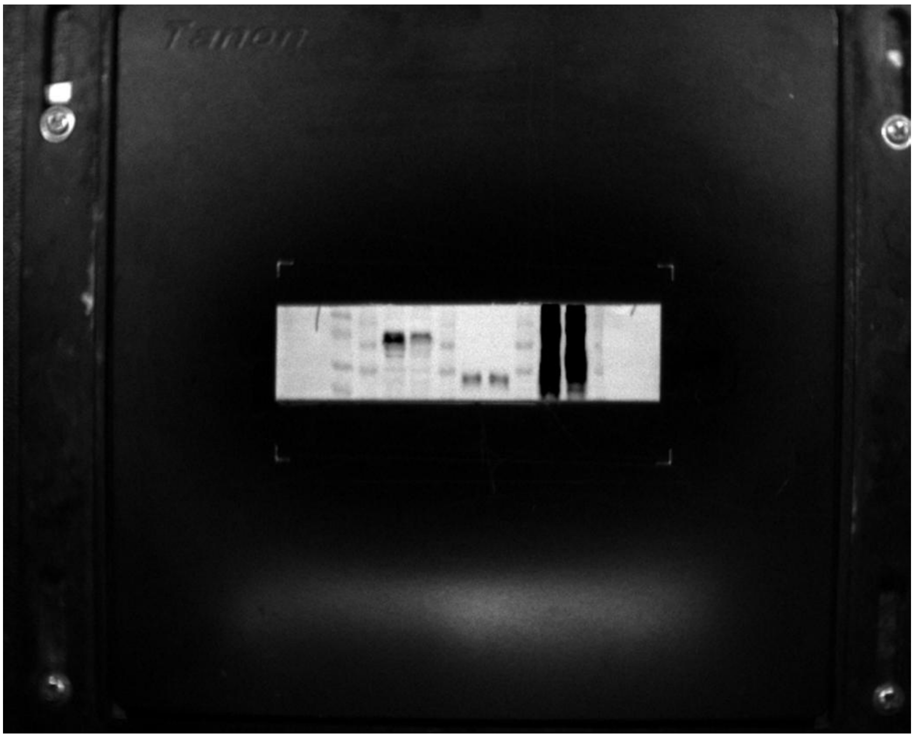

GRP75 for Fig. 7A.

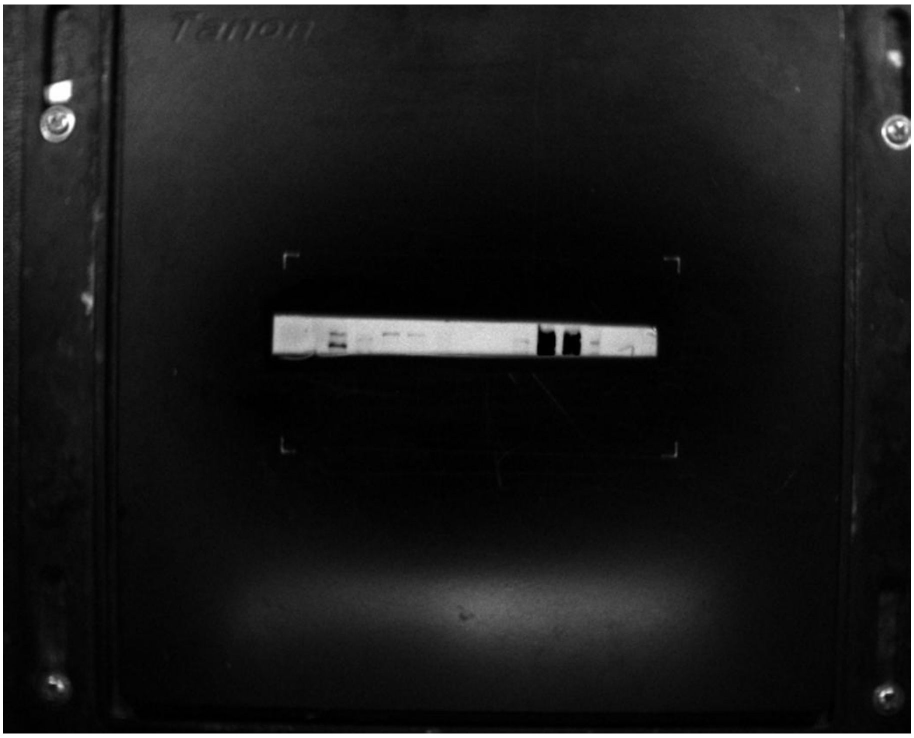

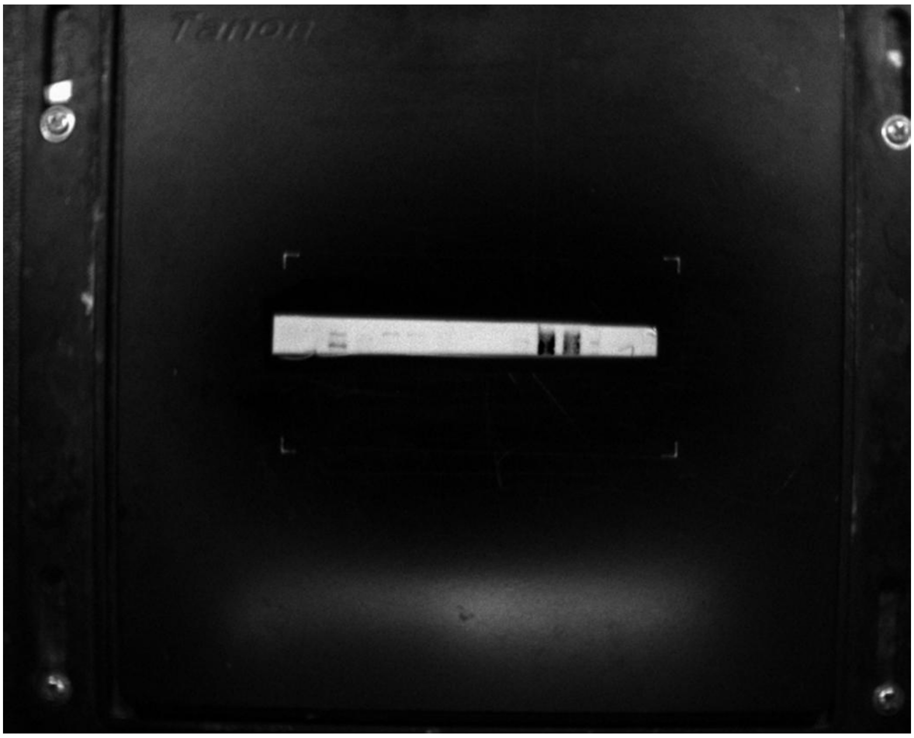

IP3R1 for Fig. 7A.

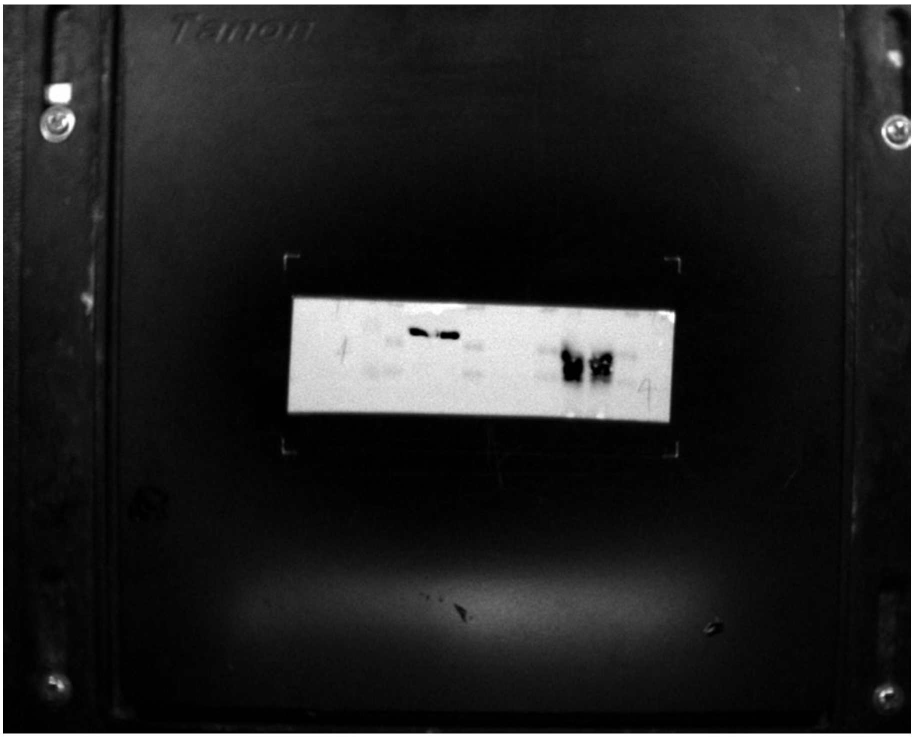

GAPDH for Fig. 7A.

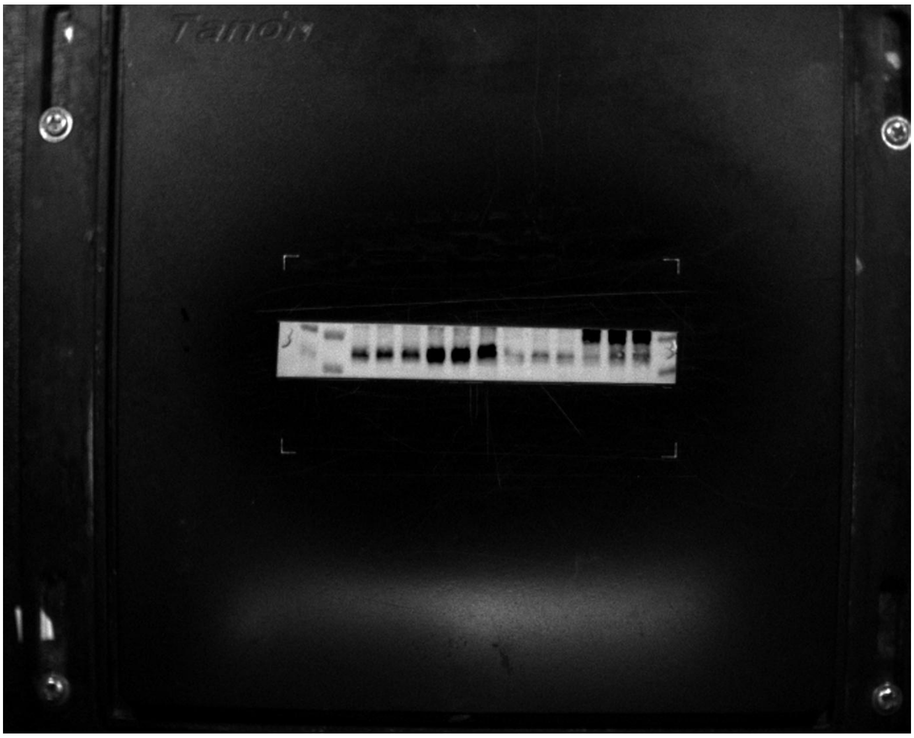

TRα for Fig. 7D.

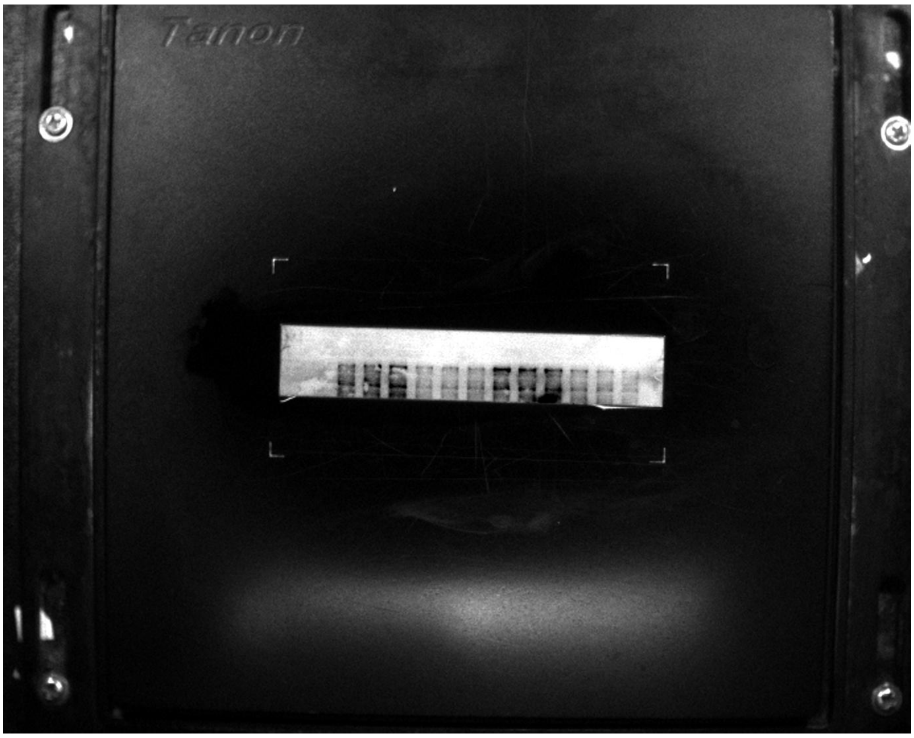

IP3R1 for Fig. 7D.

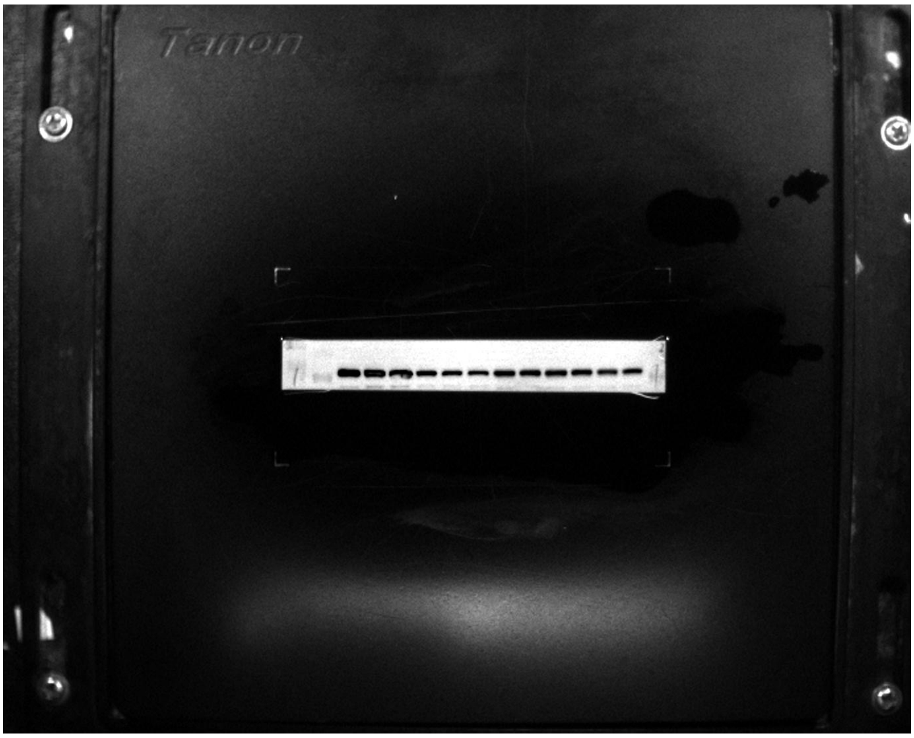

Grp75 for Fig. 7D.

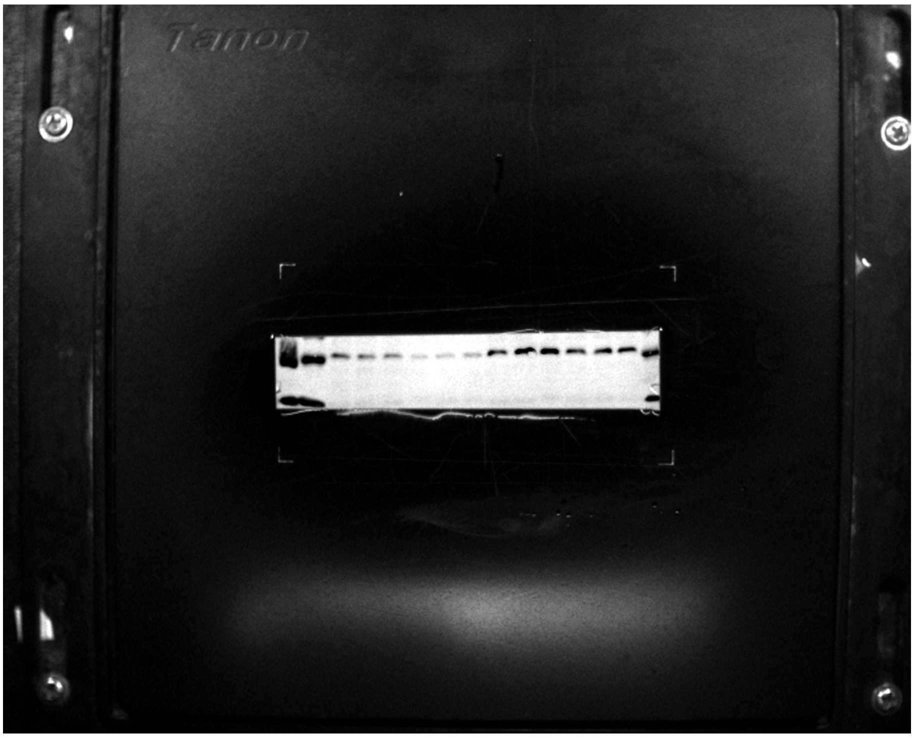

VDAC1for Fig. 7D.

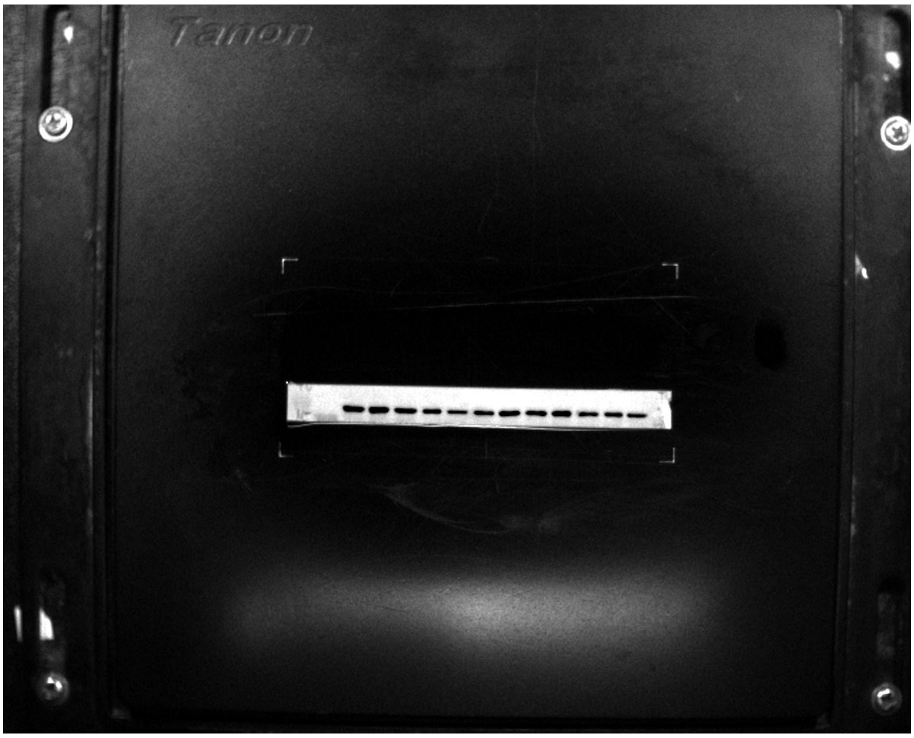

GAPDH for Fig. 7D.

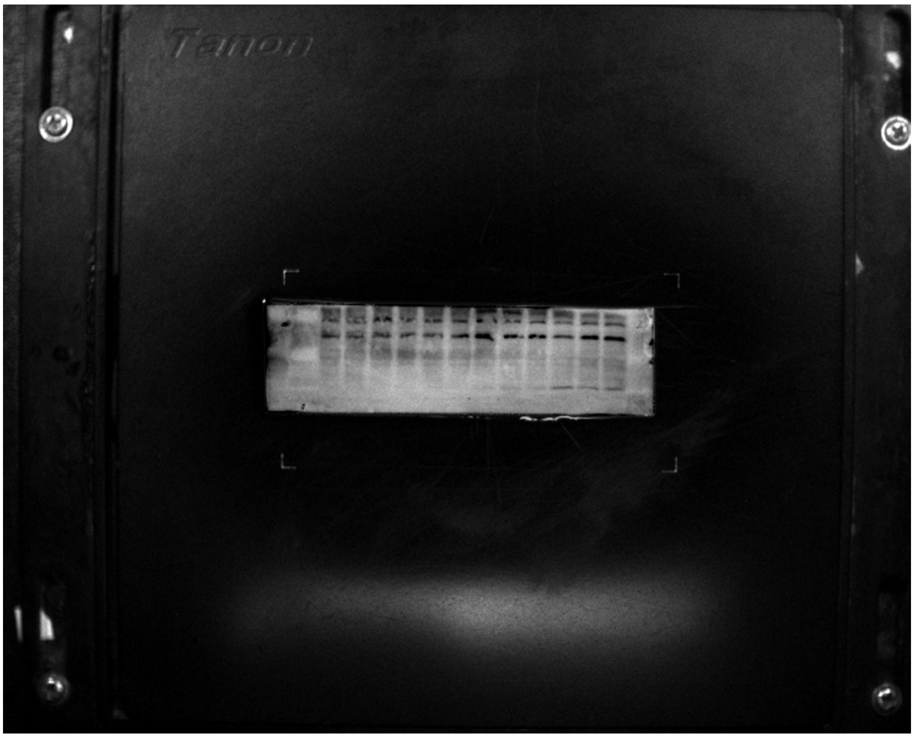

BAX for Fig. 7N.

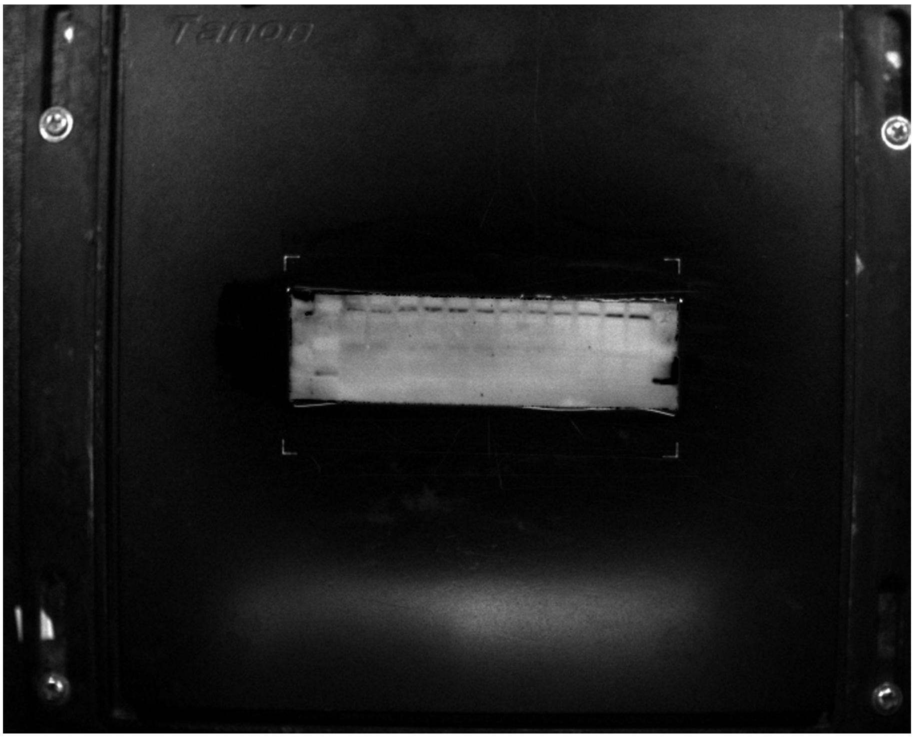

Bcl-2 for Fig. 7N.

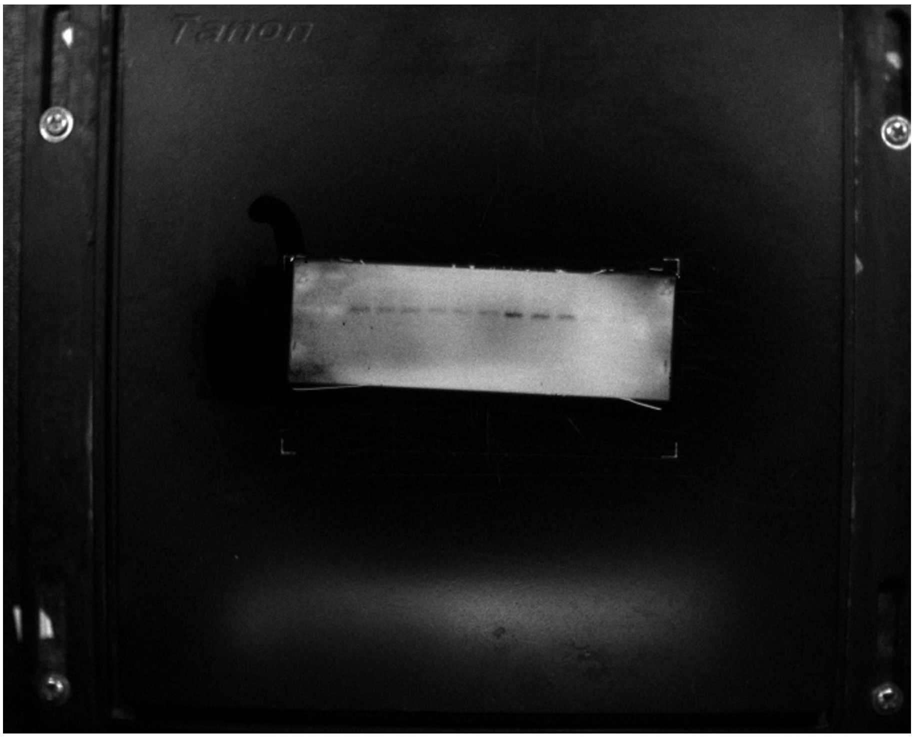

Cleaved-caspase3 for Fig. 7N.

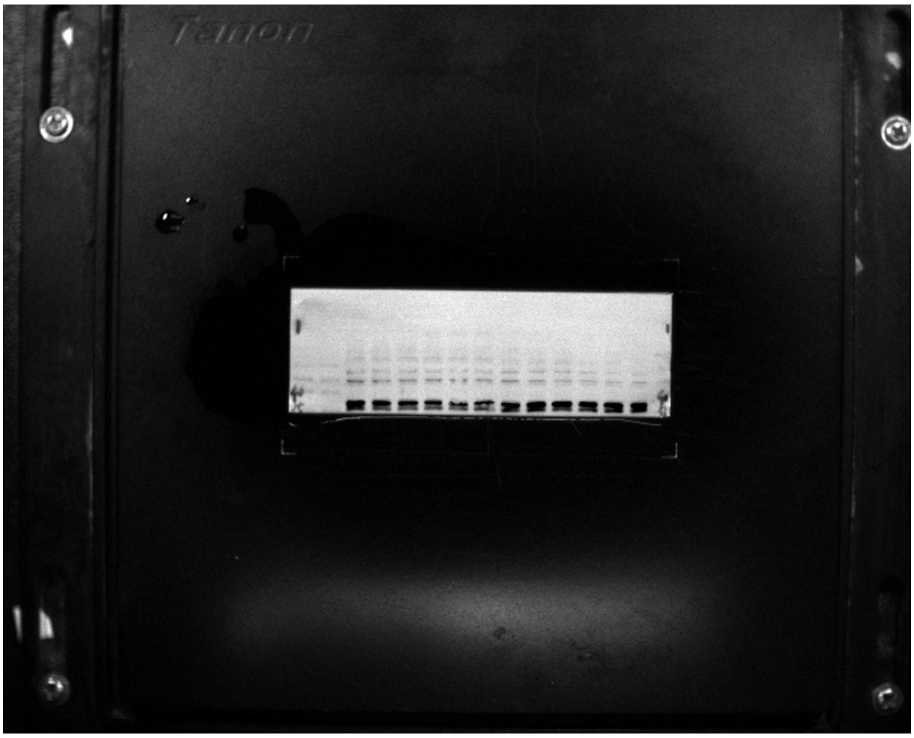

GAPDH for Fig. 7N.

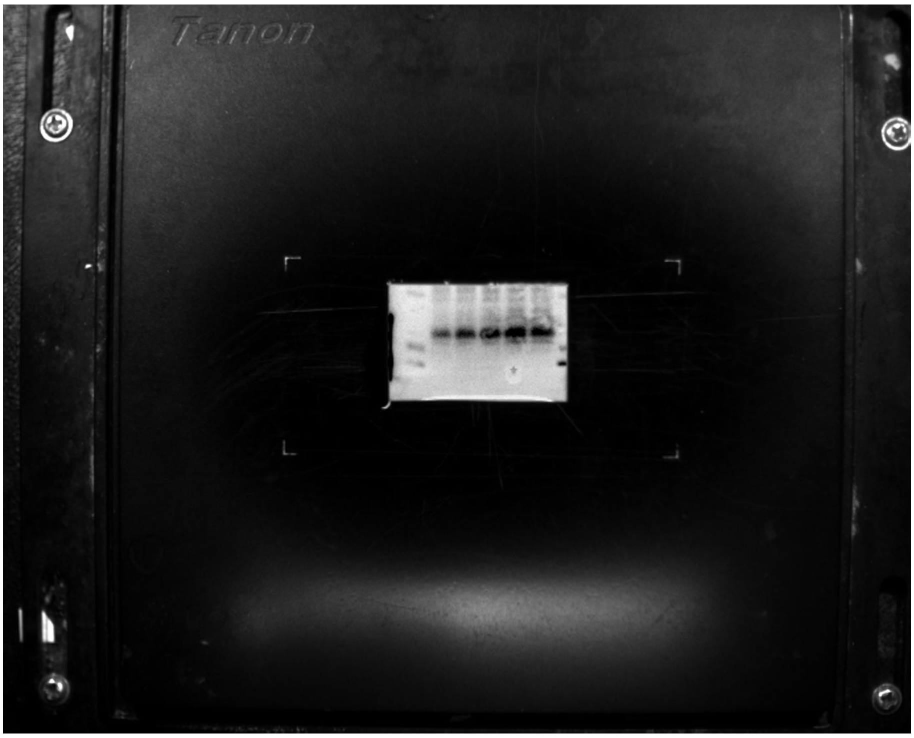

P16 for Supplementary Fig. 2D.

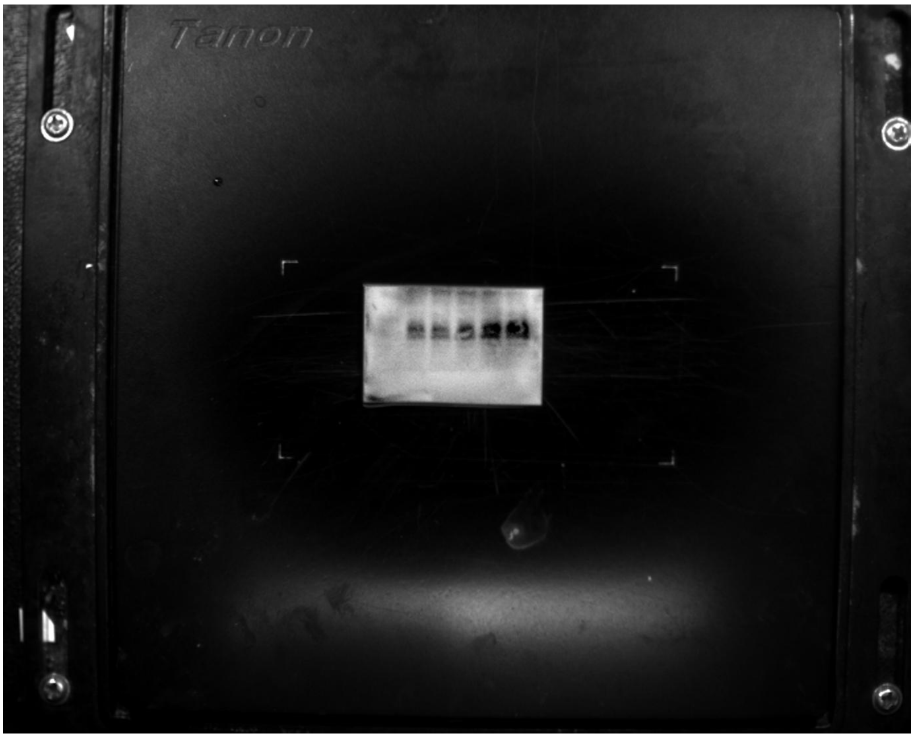

P21 for Supplementary Fig. 2D.

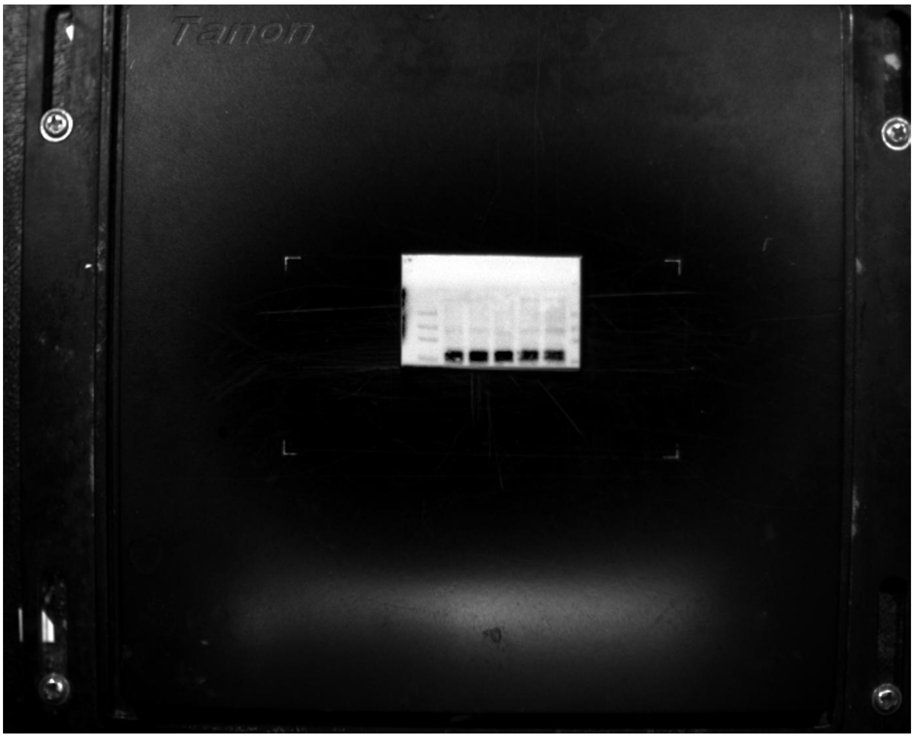

GAPDH for Supplementary Fig. 2D.

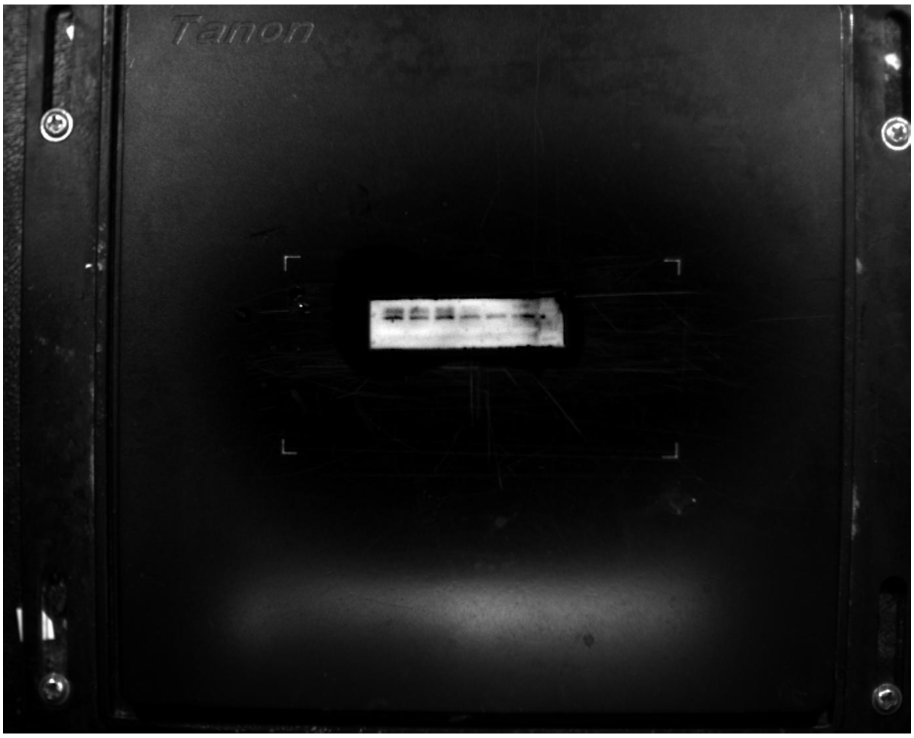

TRα for Supplementary Fig. 3C.

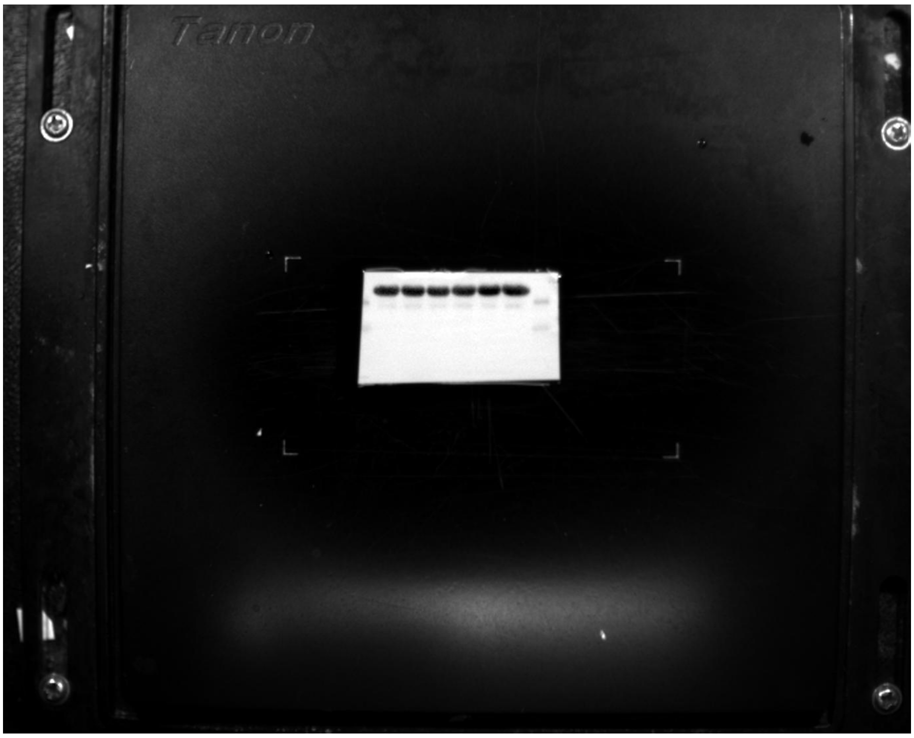

GAPDH for Supplementary Fig. 3C.

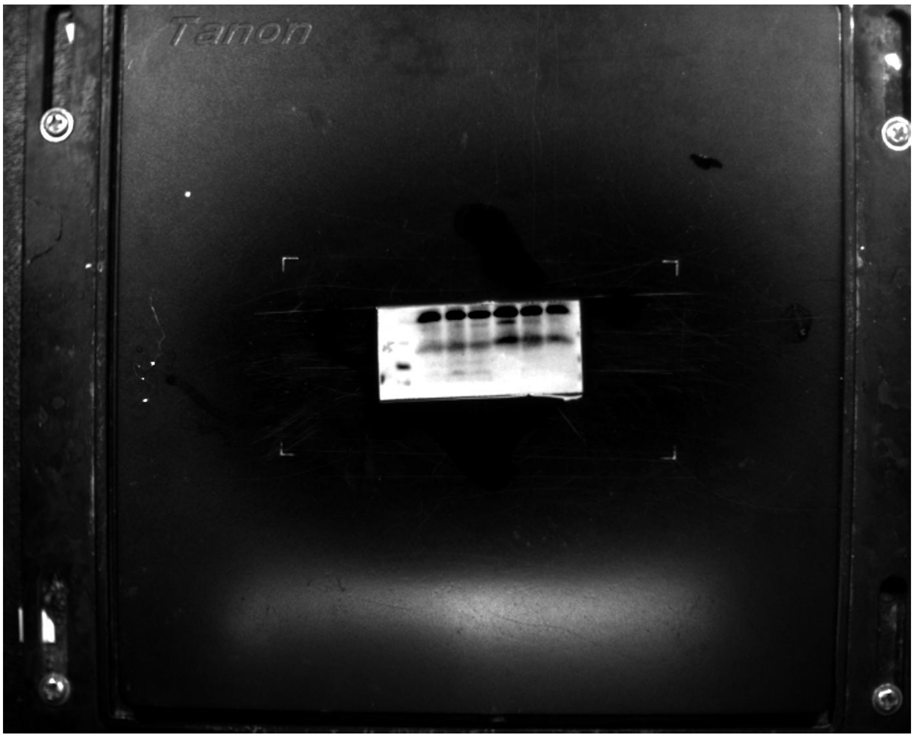

P16 for Supplementary Fig. 3G.

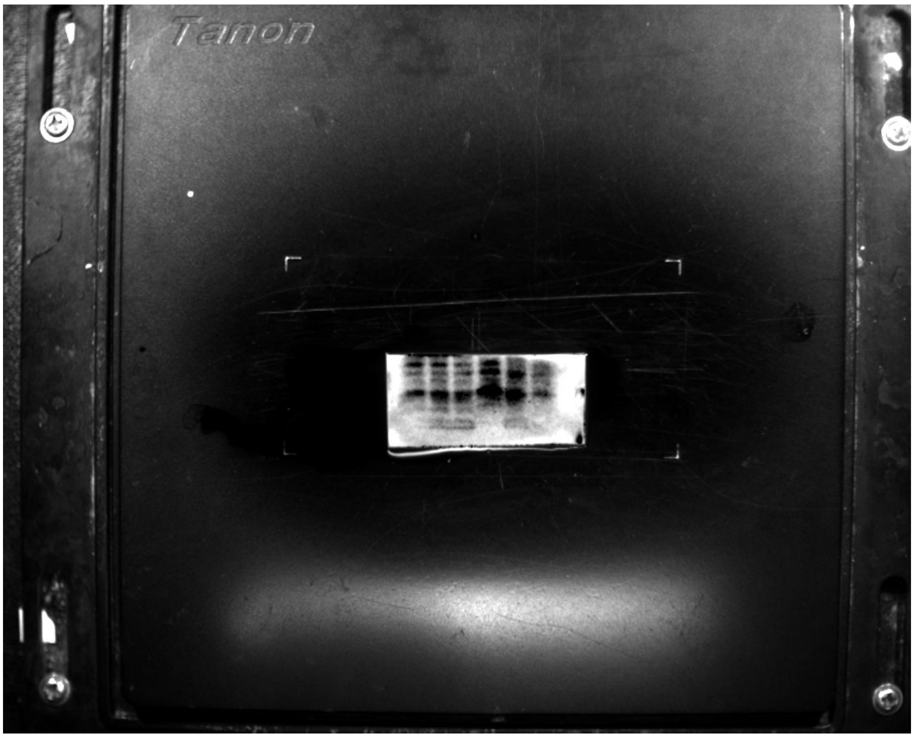

P21 for Supplementary Fig. 3G.

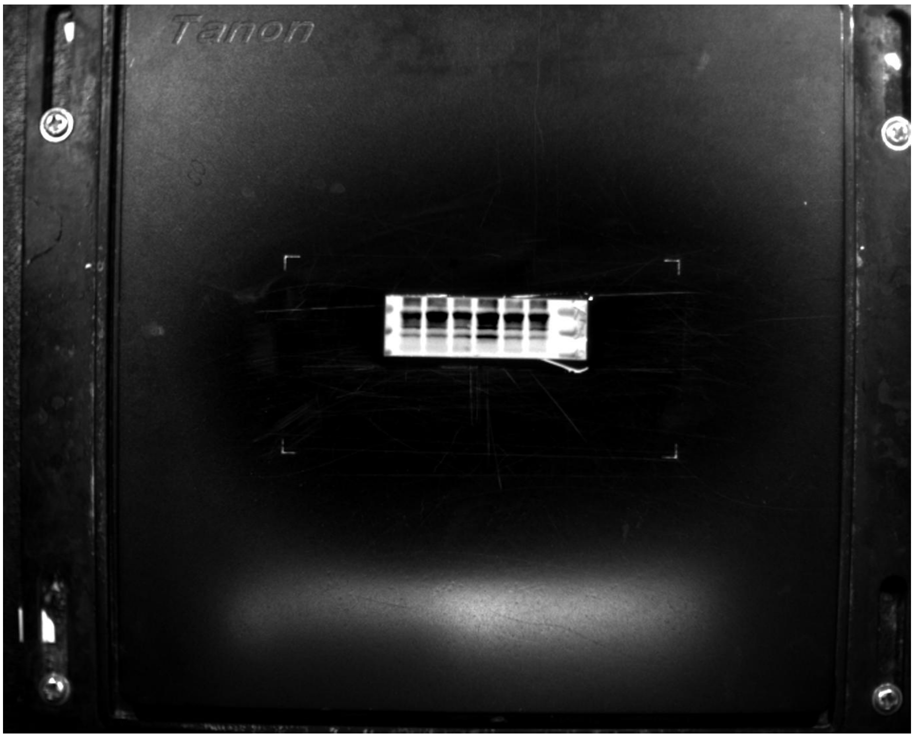

P53 for Supplementary Fig. 3G.

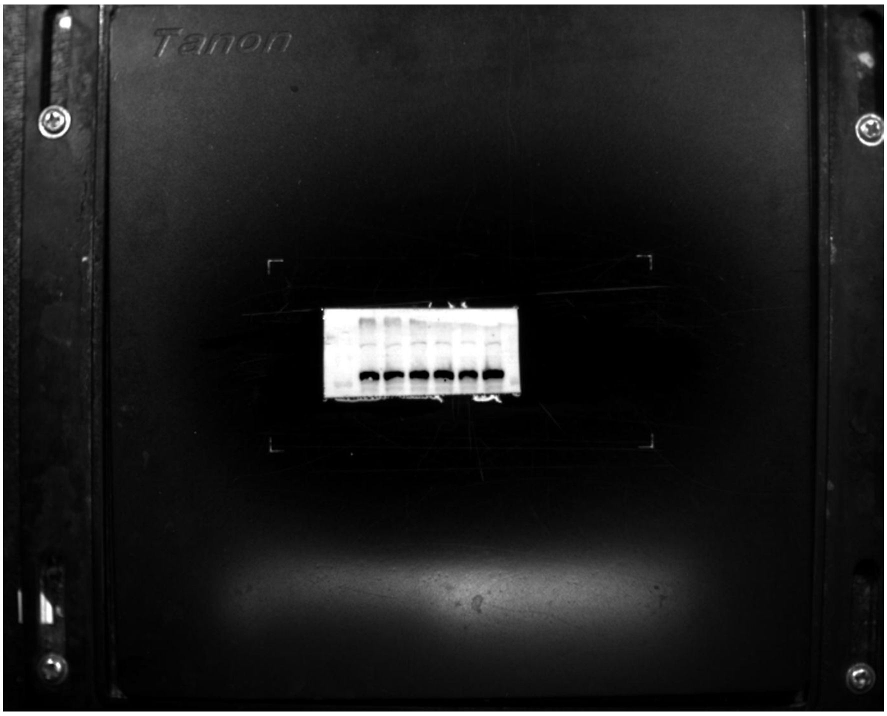

PI3K for Supplementary Fig. 3G.

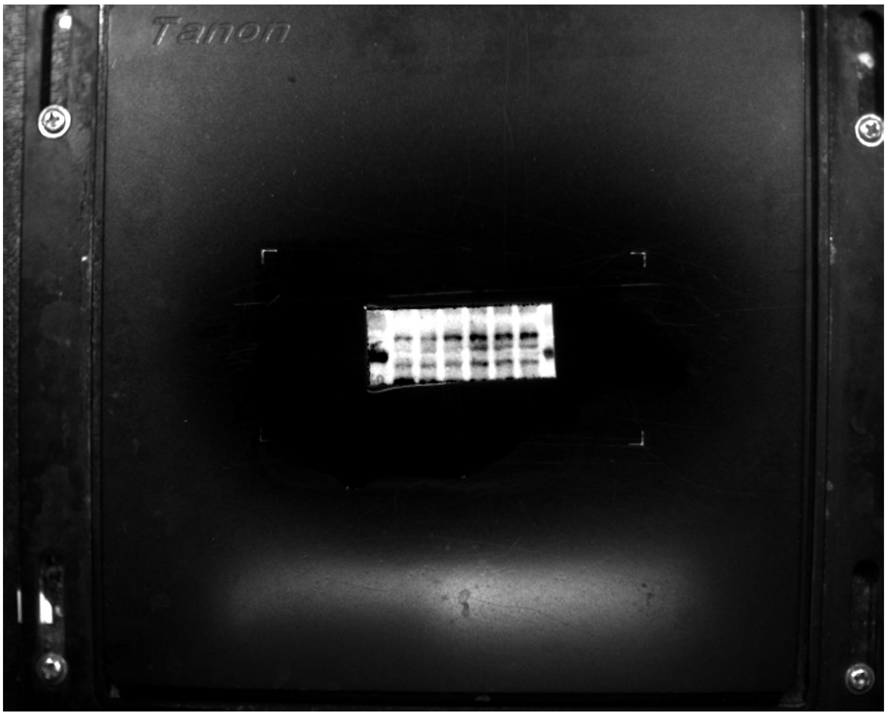

P-Akt for Supplementary Fig. 3G.

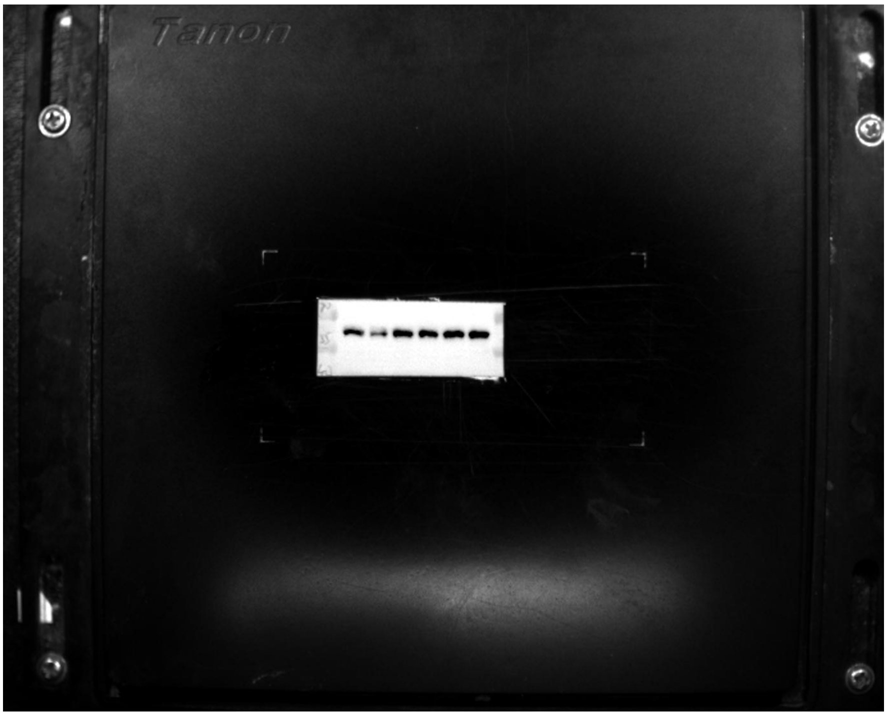

Akt for Supplementary Fig. 3G.

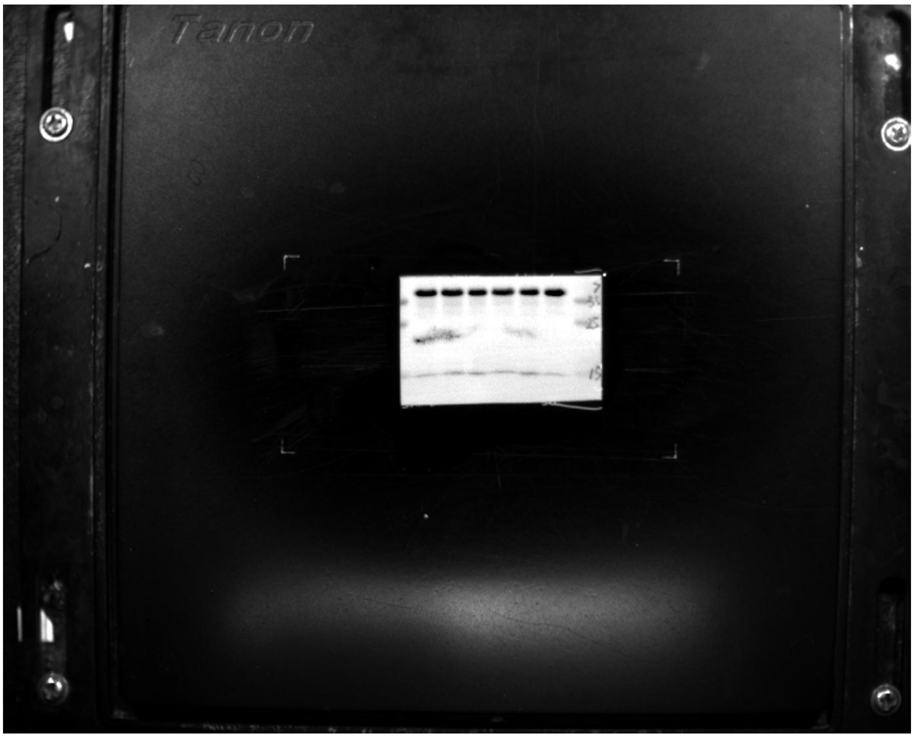
GAPDH for Supplementary Fig. 3G.

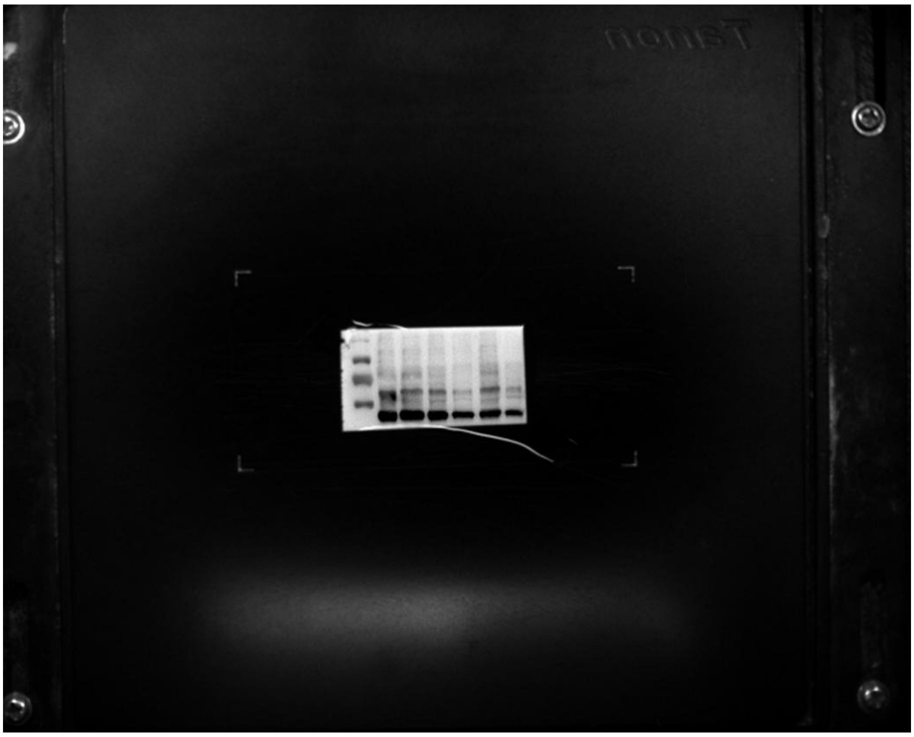
TRα for Supplementary Fig. 4B.

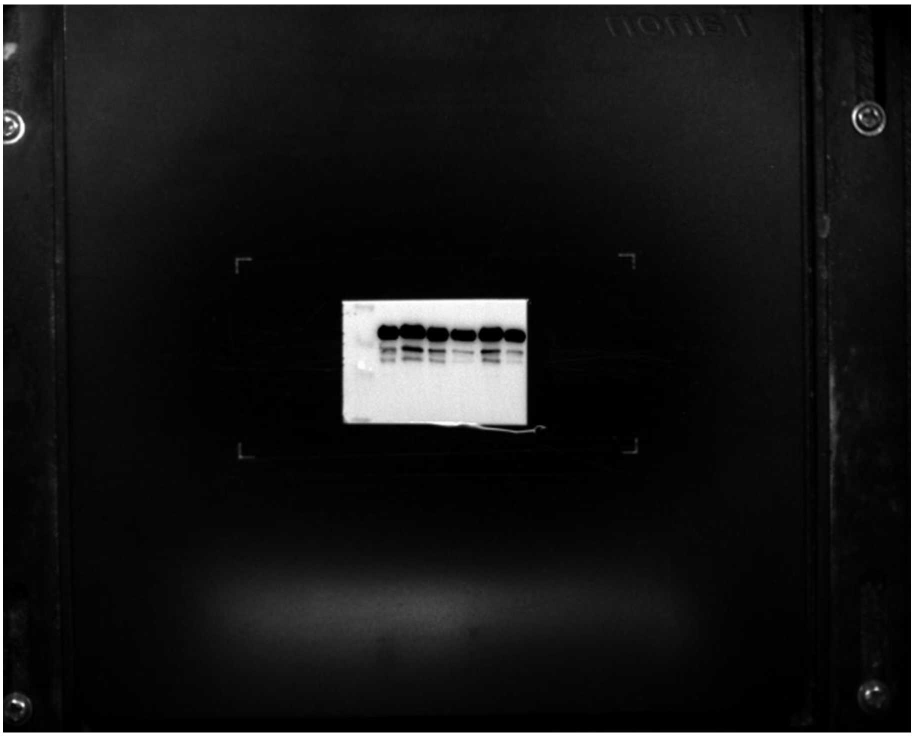

GAPDH for Supplementary Fig. 4B.

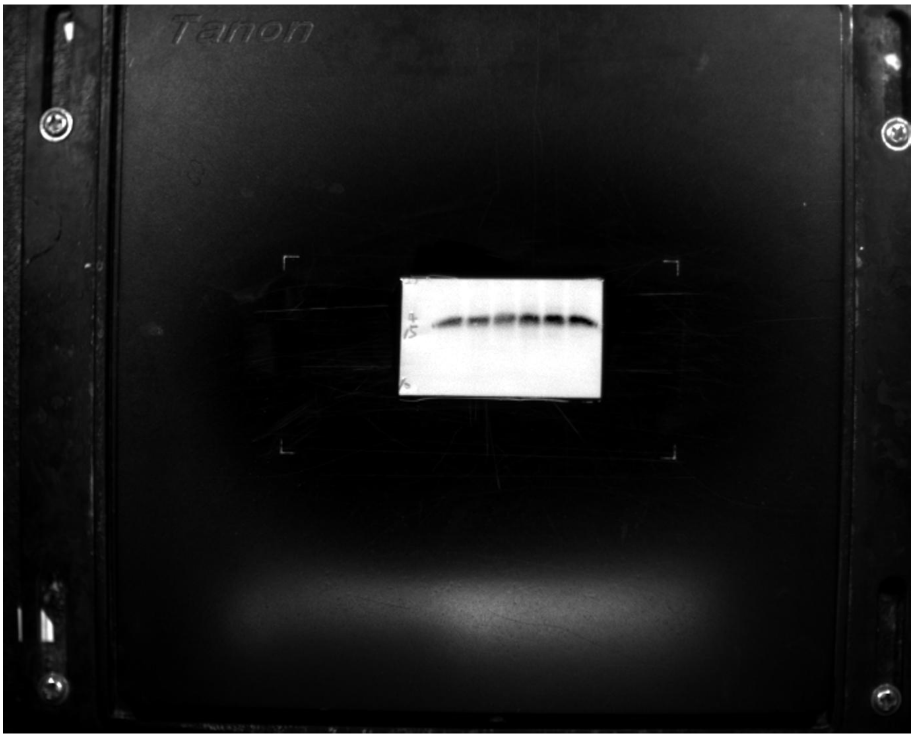

p16 for Supplementary Fig. 4D.

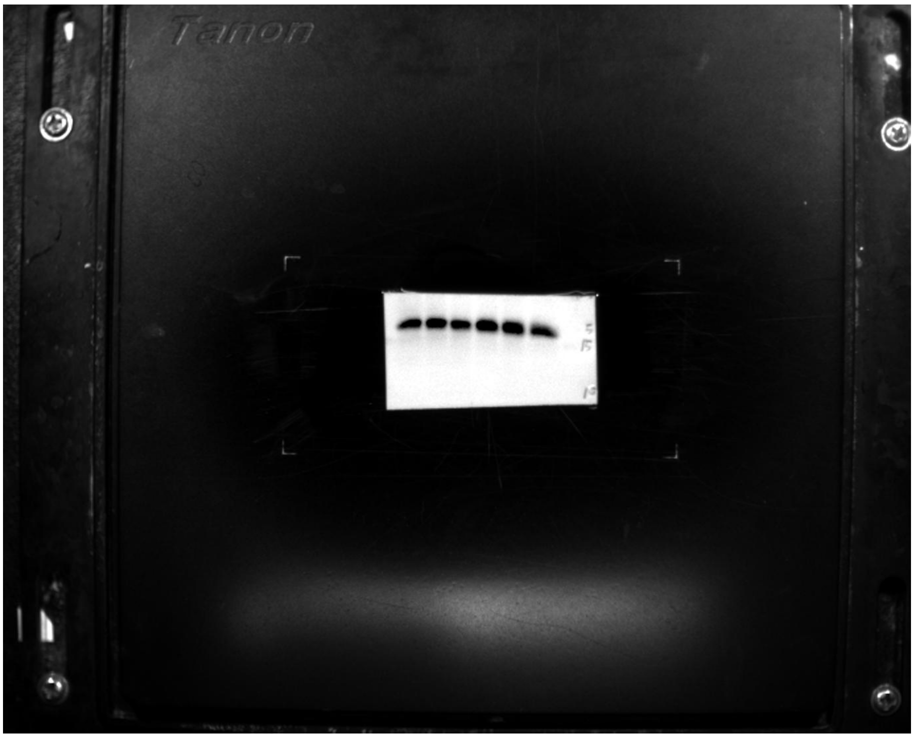

P21 for Supplementary Fig. 4D.

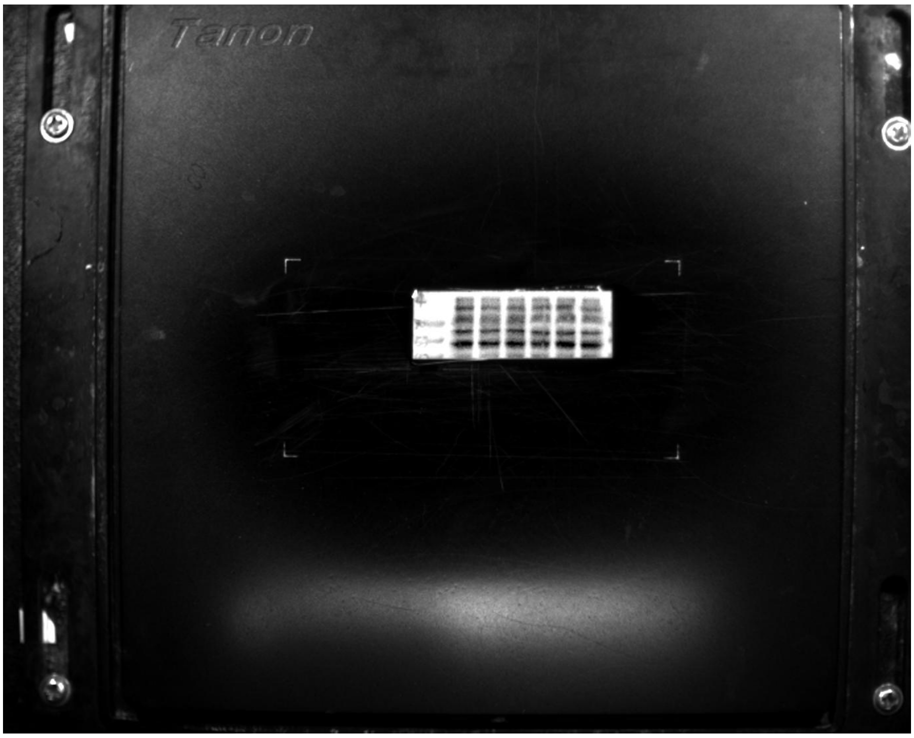

P53 for Supplementary Fig. 4D.

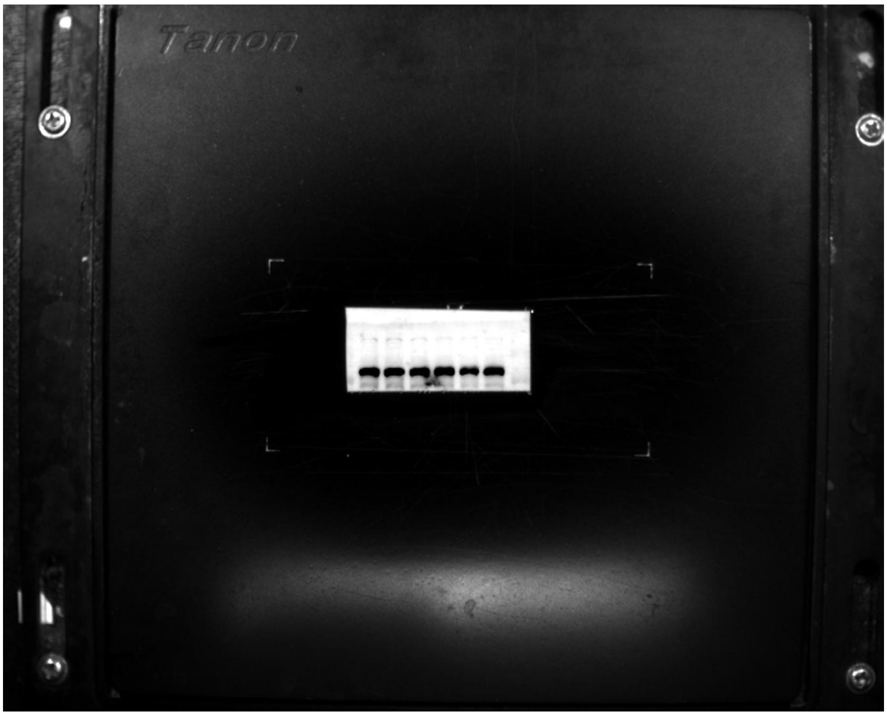

PI3K for Supplementary Fig. 4D.

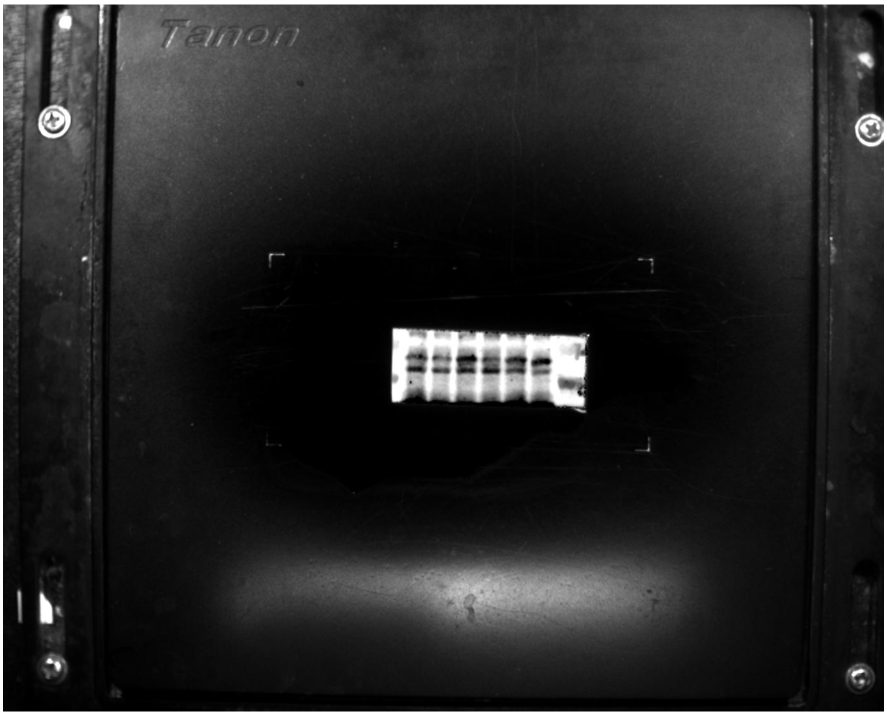

P-Akt for Supplementary Fig. 4D.

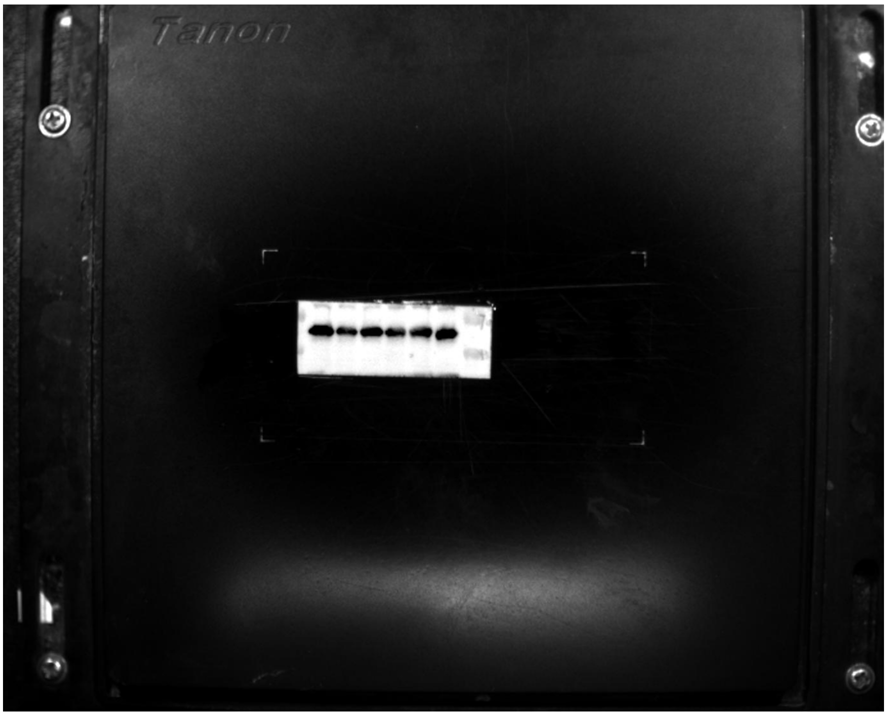

Akt for Supplementary Fig. 4D.

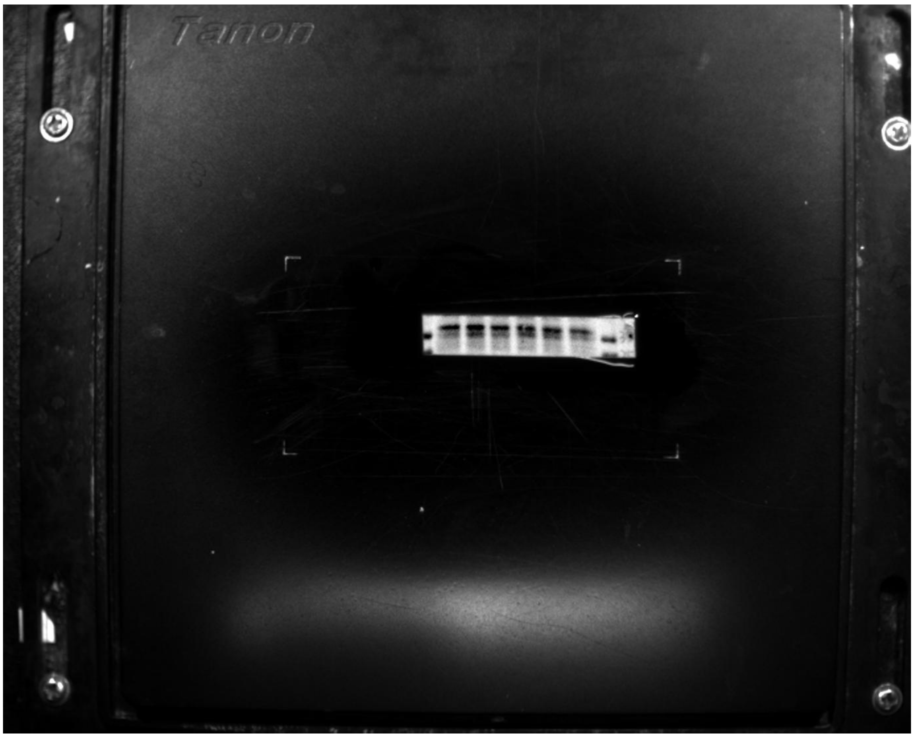

GAPDH for Supplementary Fig. 4D.

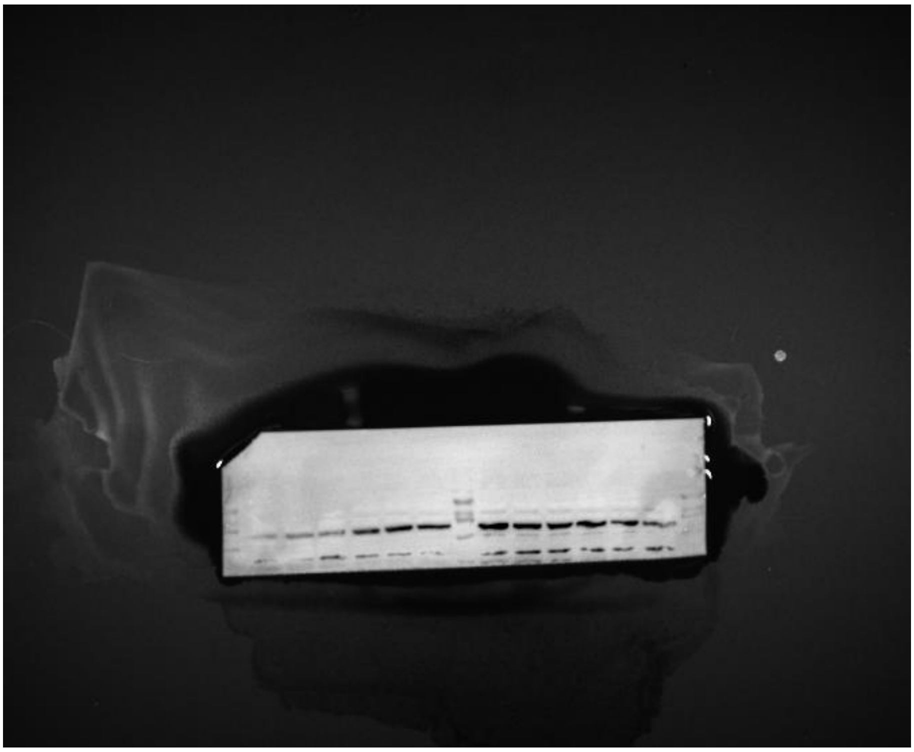

TRα for Supplementary Fig. 5B.

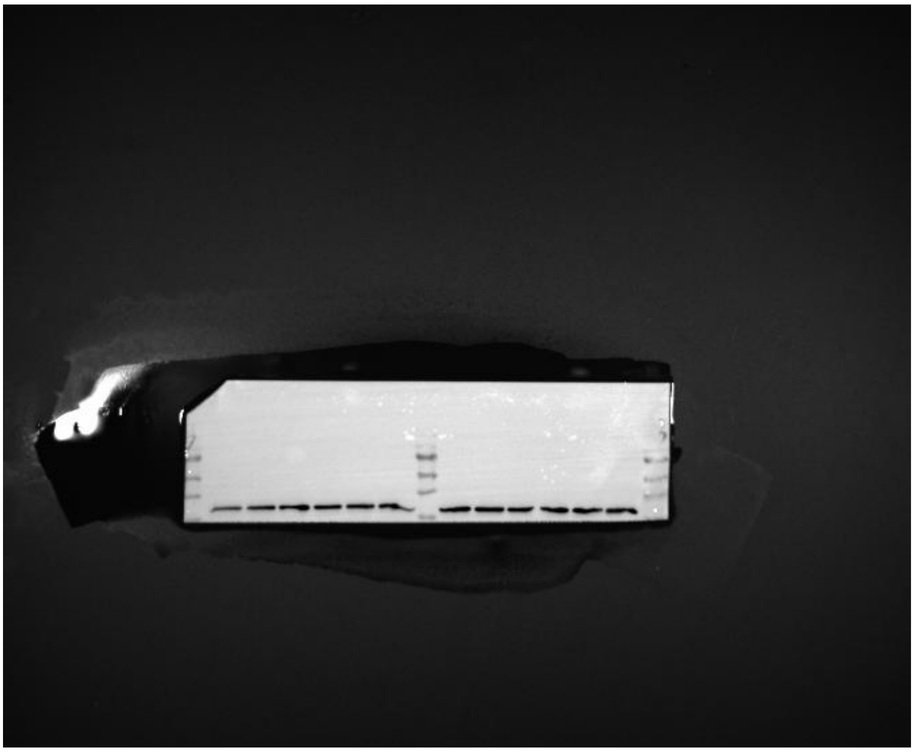

GAPDH for Supplement Fig. 5B.

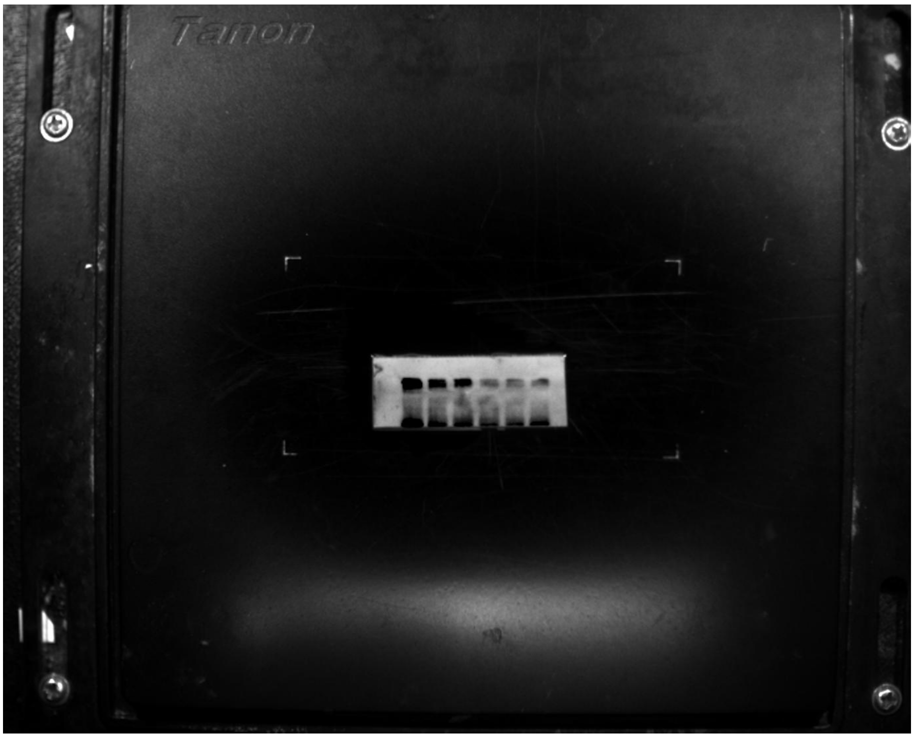

IP3R1 for Supplement Fig. 5B.

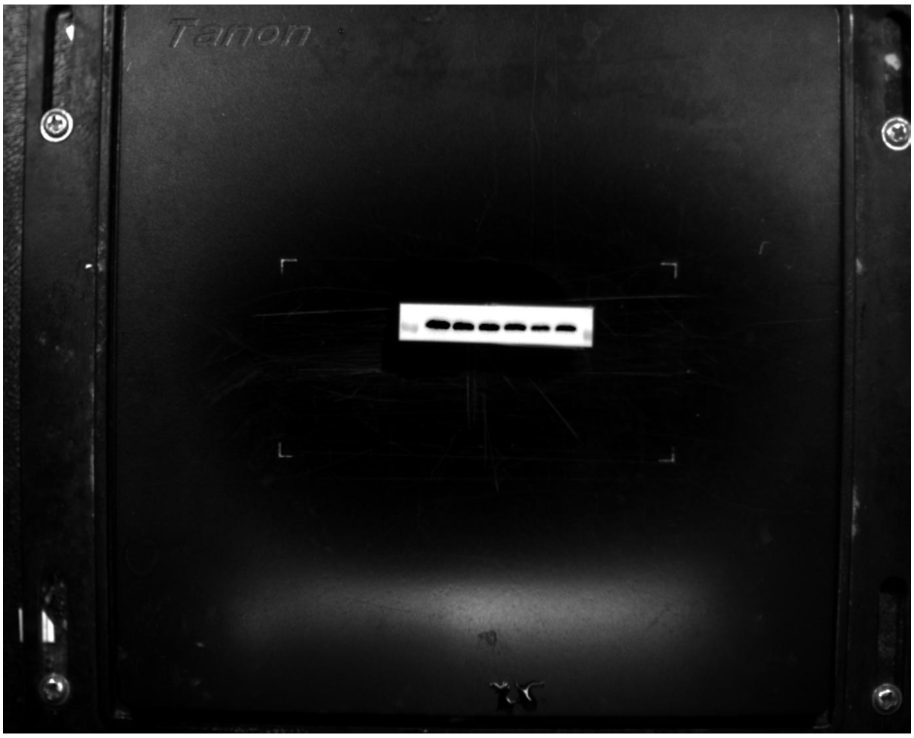

GRP75 for Supplement Fig. 5B.

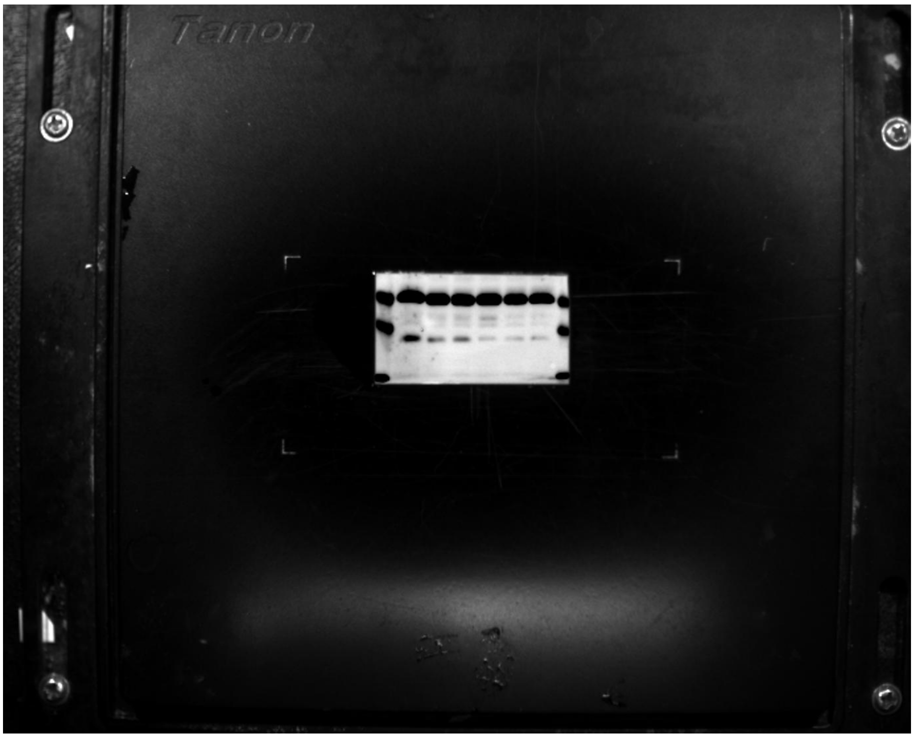

VDAC1 for Supplement Fig. 5B.

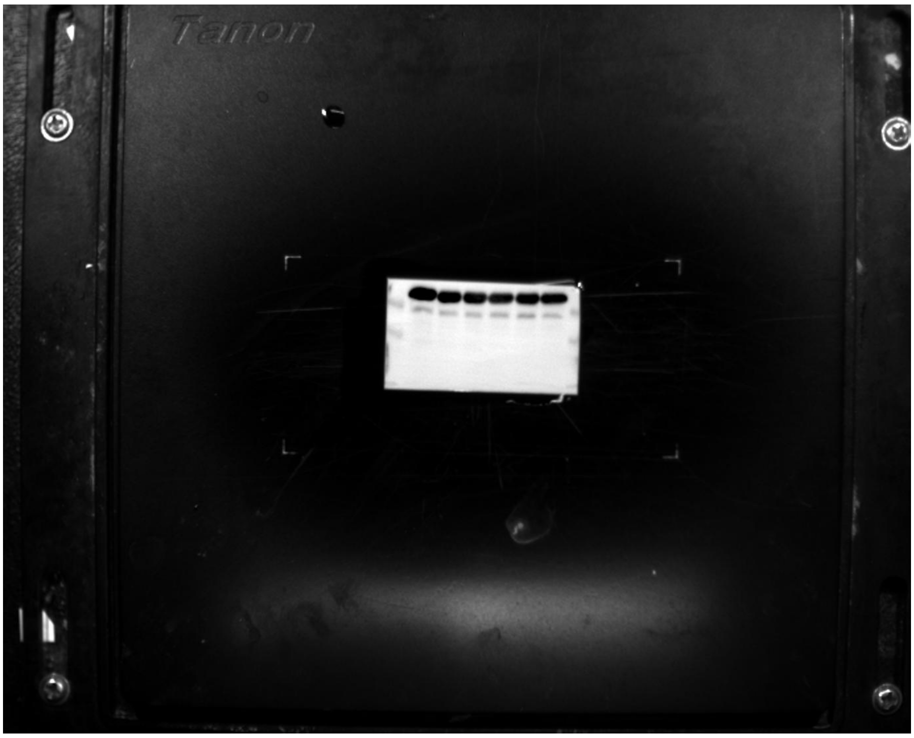

GAPDH for Supplement Fig. 5B.

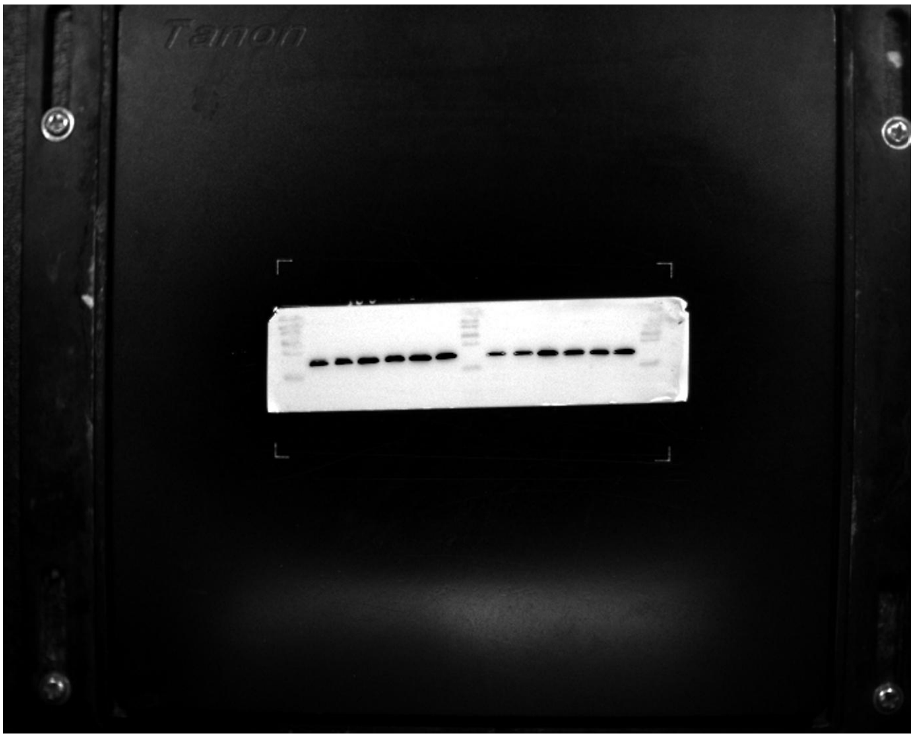

BAX for Supplement Fig. 5N.

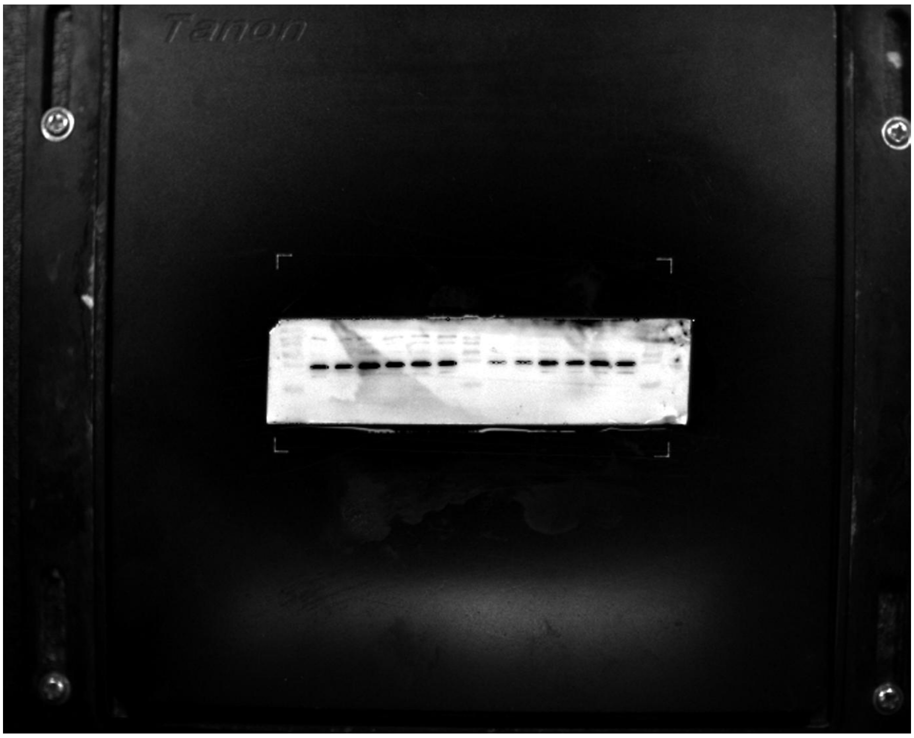

Bcl-2 for Supplement Fig. 5N.

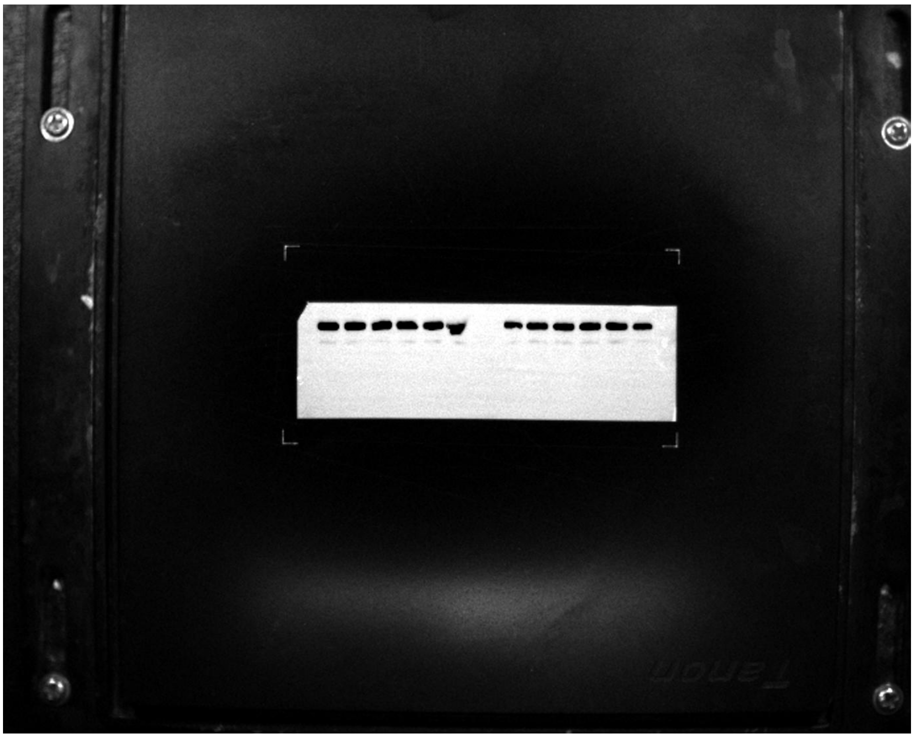

Cleaved-caspase3 and GAPDH for Supplement Fig. 5N.
