# Supplementary figures and images for: Ageing‐Dependent Thyroid Hormone Receptor α Reduction Activates IP3R1‐Meditated Ca2+ Transfer in MAM and Exacerbates Skeletal Muscle Atrophy in Mice
Source: Cell Prolif. 2025 Aug 24;59(5):e70120. doi: 10.1111/cpr.70120 (PMC13114768; doi:10.1111/cpr.70120)

**A**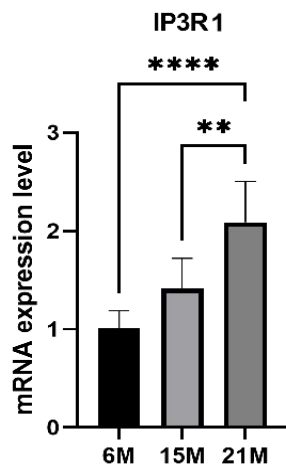**B**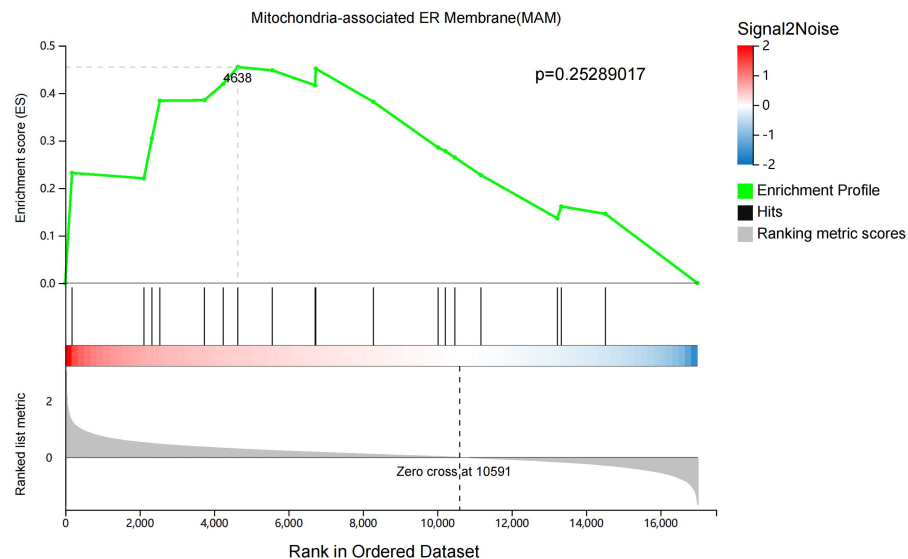**C**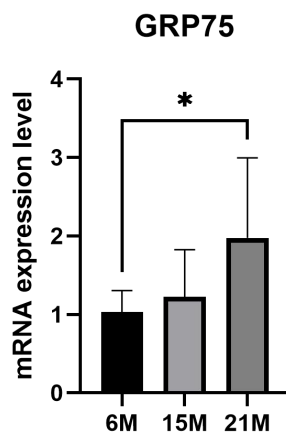**E**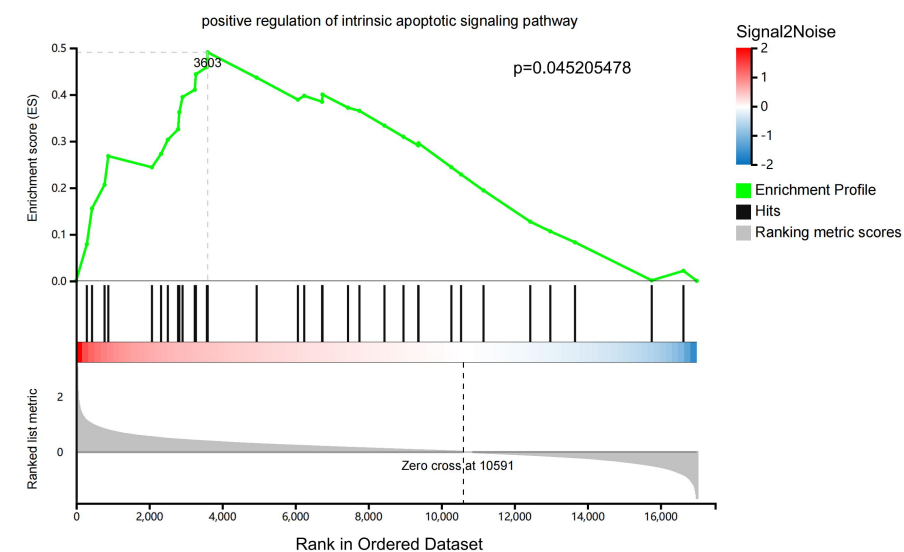**D**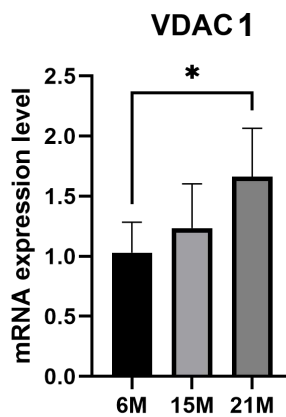

Supplement: Supplementary file 2 — FIGURE S1: (A) mRNA levels of IP3R1 in GA muscle of mice at different months of age (n = 8). (B) GSEA on the GO of “mitochondria‐associated ER membrane (MAM)” is shown. (C, D) mRNA levels of Grp75 and VDAC1 in GA muscle of mice at different months of age (n = 8). (E) GSEA on the GO of “positive regulation of intrinsic apoptotic signalling pathway” is shown. One‐way ANOVA; *p < 0.05, **p < 0.01 and ****p < 0.0001. [file CPR-59-e70120-s005.pdf]

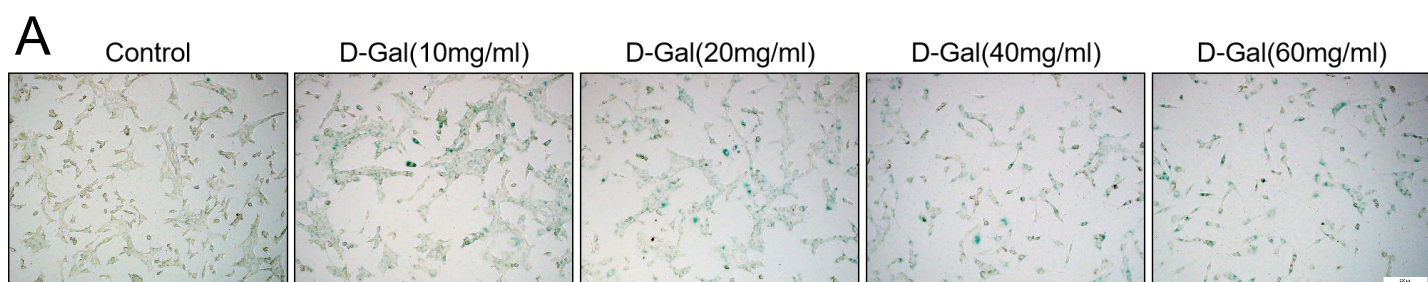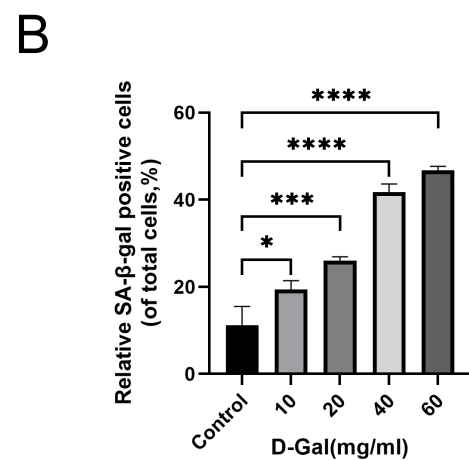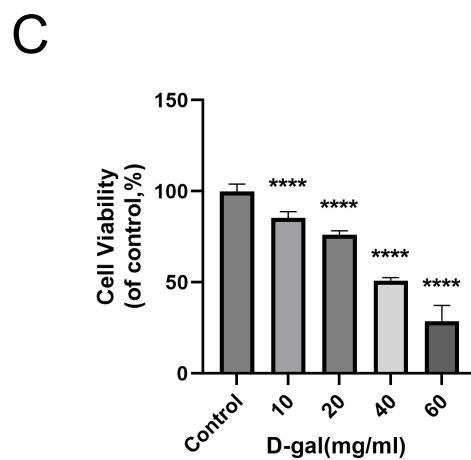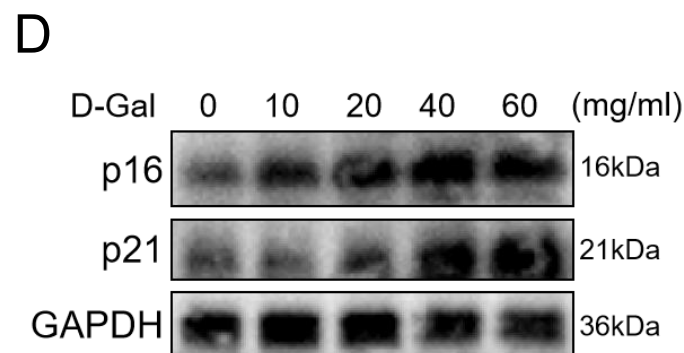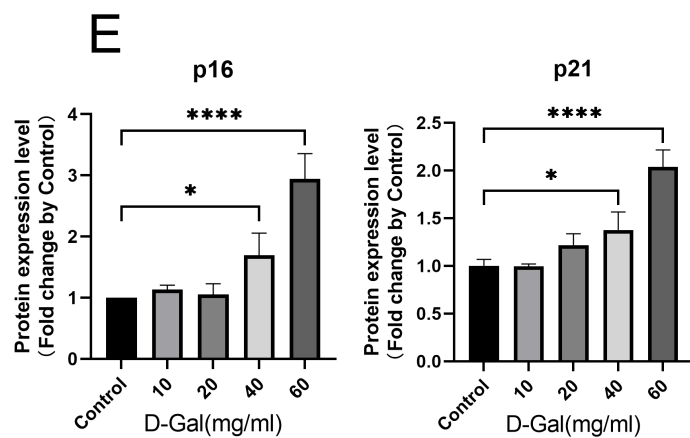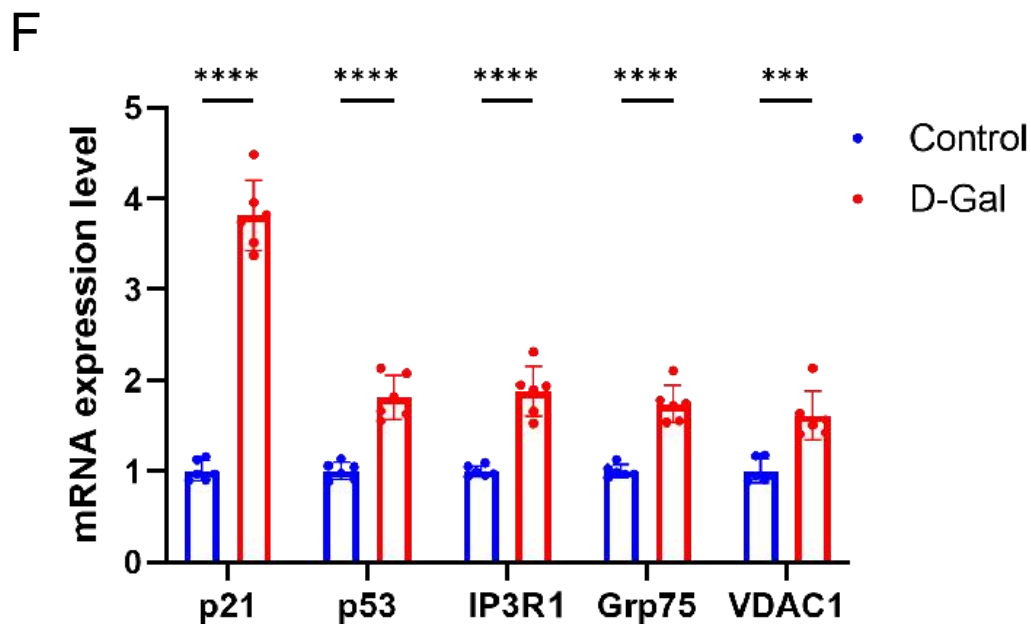

Supplement: Supplementary file 3 — FIGURE S2: 40 mg/mL is the optimal concentration of D‐Gal to induce C2C12 cell senescence. (A, B) Representative images of β‐galactosidase staining of C2C12 cells and corresponding statistics of the proportion of positive cells (scale bar: 200 μm, n = 3). (C) The cell survival rate was detected by CCK8 assay (n = 5). (D, E) p16, p21 and GAPDH protein expressions measured using WB in C2C12 cells with corresponding statistics (n = 3). (F) mRNA levels of p21, p53, IP3R1, Grp75 and VDAC1 of control and D‐Gal group in C2C12 cells (n = 6). *p < 0.05, ***p < 0.001 and ****p < 0.0001. [file CPR-59-e70120-s003.pdf]

**A**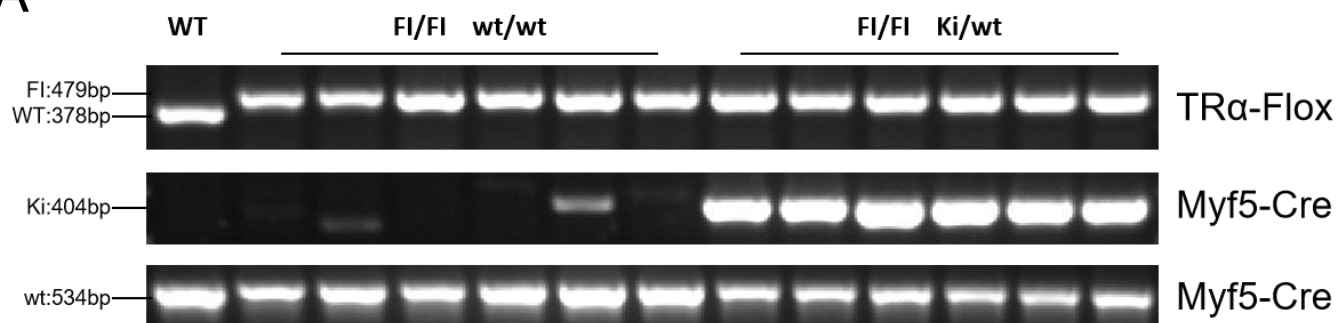**B**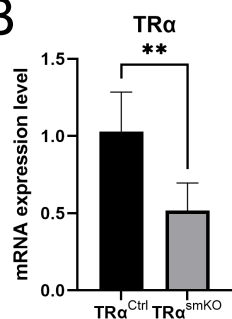**C**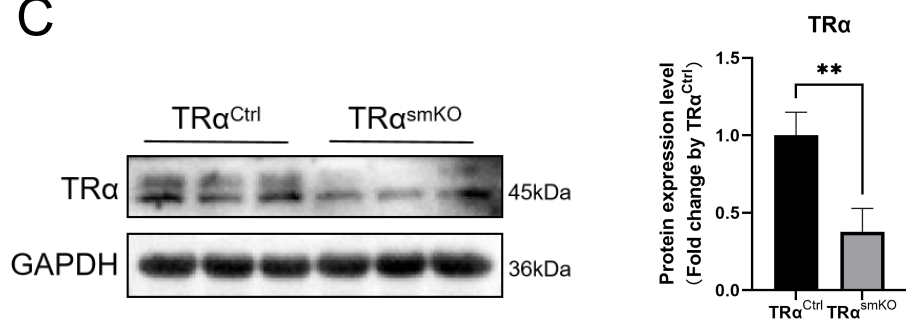**D**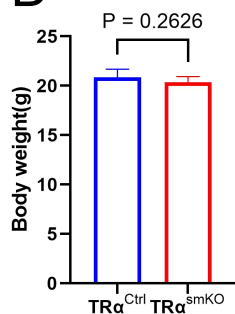**E**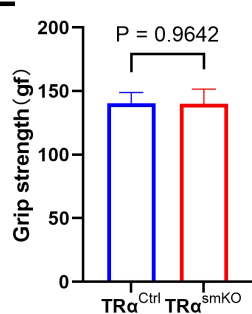**F**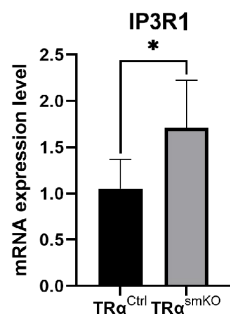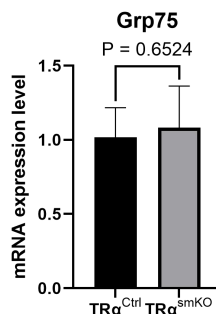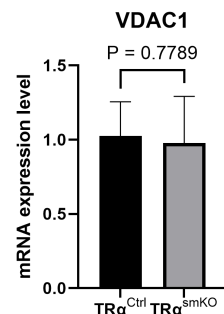**G**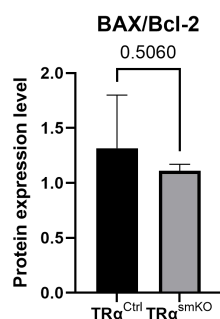**H**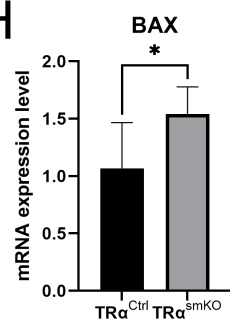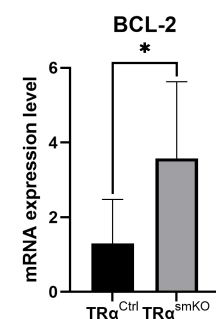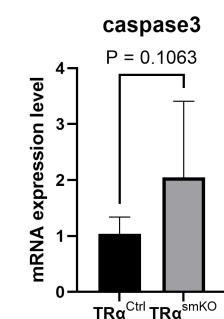**I**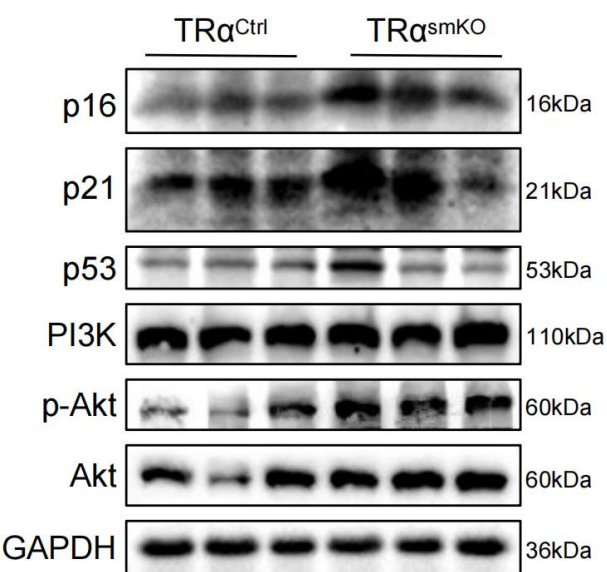**J**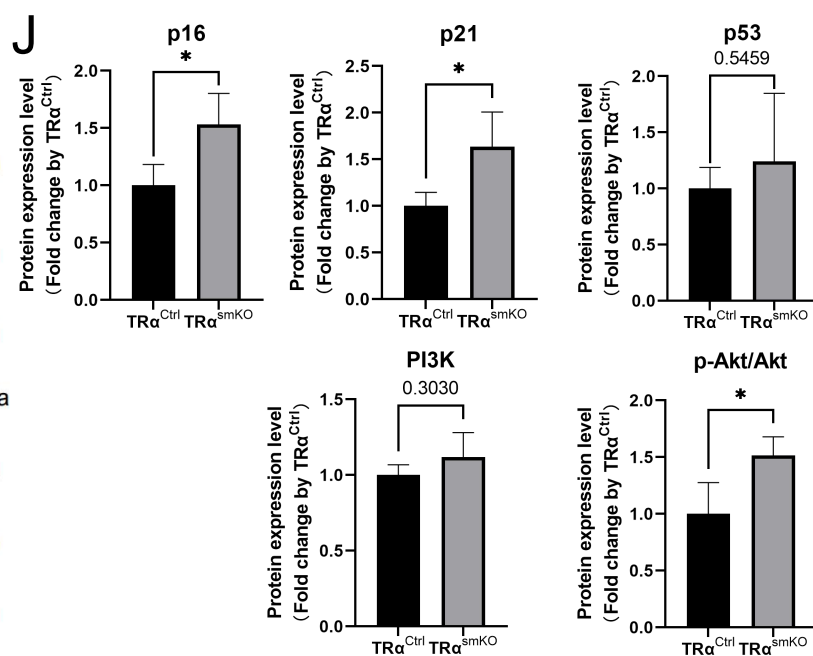

Supplement: Supplementary file 4 — FIGURE S3: Identification of TRα skeletal‐muscle‐specific knockout mice. (A) Gel plot of mouse tail genotype identification. (B) mRNA level of TRα in GA muscle of mice (n = 6). (C) Protein level of TRα in GA muscle of mice (n = 3). (D, E) Body weight and grip strength of mice (n = 6). (F) mRNA levels of IP3R1, Grp75 and VDAC1 in GA muscle (n = 6). (G) Protein expression ratio of BAX to Bcl‐2 in mice GA muscle (n = 3). (H) mRNA levels of BAX, Bcl‐2 and caspase3 in GA muscle (n = 6). (I, J) Protein level of p16, p21, p53, PI3K, p‐Akt and Akt in GA muscle of mice (n = 3). Unpaired Student's t‐tests; *p < 0.05 and **p < 0.01. [file CPR-59-e70120-s006.pdf]

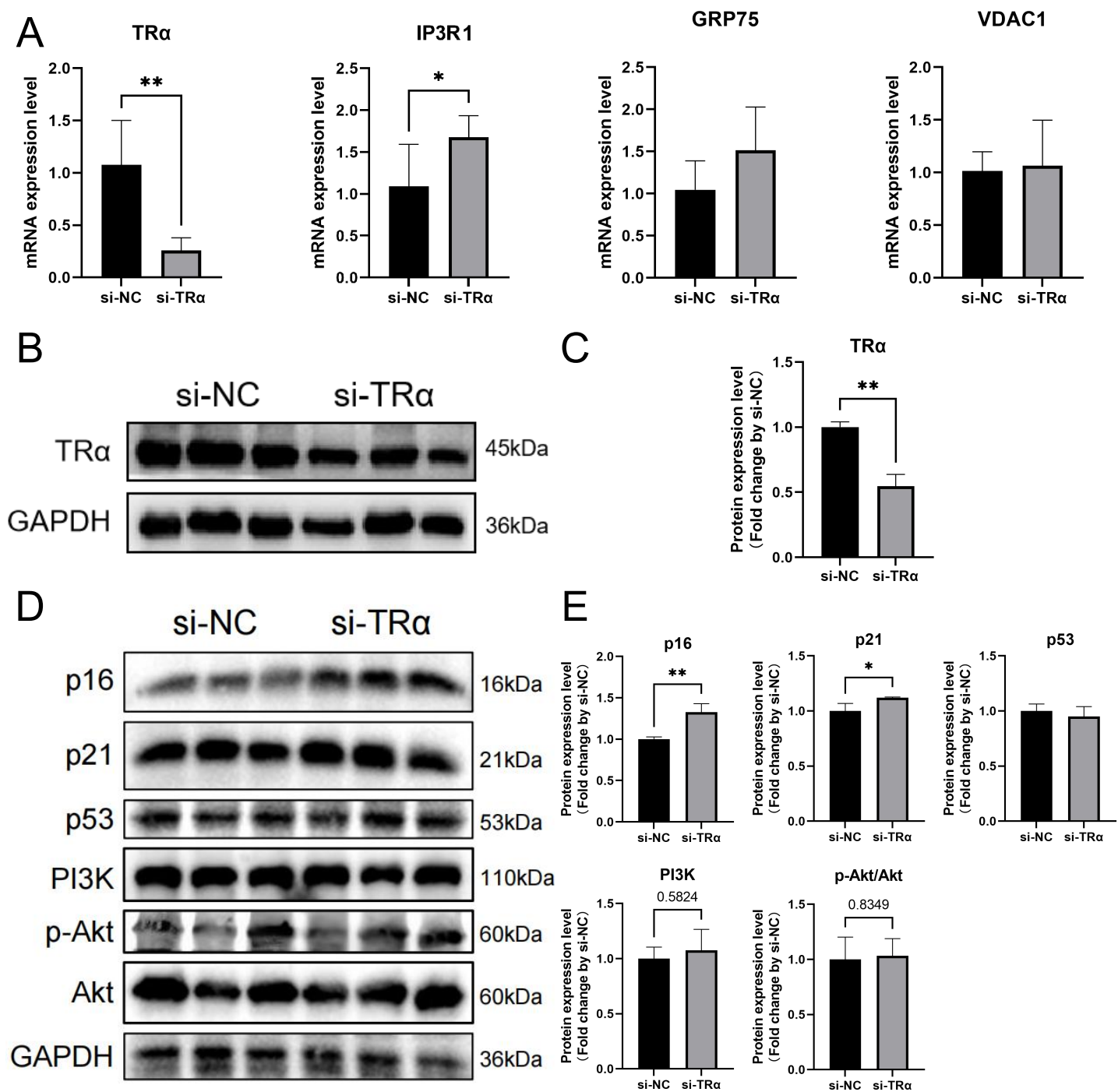

Supplement: Supplementary file 5 — FIGURE S4: (A) mRNA levels of TRα, IP3R1, Grp75 and VDAC1 in C2C12 cells (n = 6). (B, C) TRα and GAPDH protein expressions measured using WB in C2C12 cells with corresponding statistics (n = 3). (D, E) p16, p21, p53, PI3K, p‐Akt and Akt protein expressions measured using WB in C2C12 cells with corresponding statistics (n = 3). Unpaired Student's t‐tests; *p < 0.05 and **p < 0.01. [file CPR-59-e70120-s007.pdf]

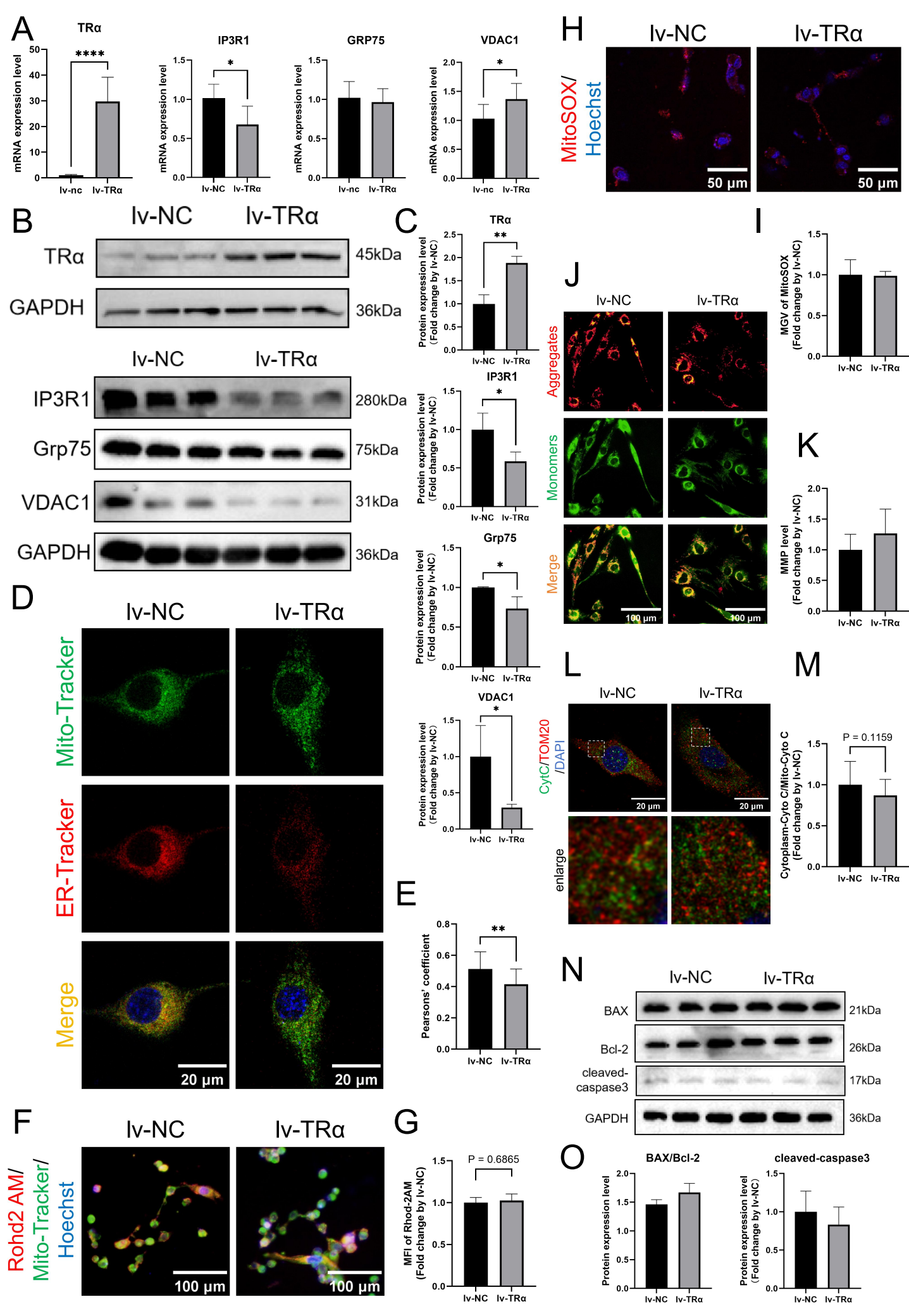

Supplement: Supplementary file 6 — FIGURE S5: Overexpression of TRα reduced IP3R1 expression and MAMs formation. (A) mRNA levels of TRα, IP3R1, Grp75 and VDAC1 in C2C12 cells (n = 6). (B) TRα, IP3R1, Grp75, VDAC1 and GAPDH protein expressions measured using WB in C2C12 cells with corresponding statistics (n = 3). (D, E) ER‐Tracker Red and Mito‐Tracker Green co‐labelling to observe MAMs, and Pearson's correlation coefficient was calculated (scale bar: 20 μm, n = 3). (F, G) Representative images of Mito‐Tracker green co‐stained with Rhod2 AM staining and corresponding red fluorescence intensity analysis (scale bar: 100 μm, n = 3). (H, I) Results of MitoSOX staining and corresponding red fluorescence intensity analysis (scale bar: 50 μm, n = 3). (J, K) Results of JC‐1 staining and corresponding red‐green fluorescence ratio analysis (scale bar: 100 μm, n = 3). (L, M) Representative images of TOM20 co‐stained with CytC in C2C12 cells, and the ratio of CytC fluorescence that did not colocalize with TOM20 was counted (scale bar: 20 μm, n = 3). (N, O) BAX, Bcl‐2, and cleaved‐caspase3 protein expressions measured using WB in C2C12 cells with corresponding statistics (n = 3). Unpaired Student's t‐tests; *p < 0.05, **p < 0.01 and ****p < 0.0001. [file CPR-59-e70120-s004.pdf]
