# Supplementary material for: Ageing‐Dependent Thyroid Hormone Receptor α Reduction Activates IP3R1‐Meditated Ca2+ Transfer in MAM and Exacerbates Skeletal Muscle Atrophy in Mice
Source: Cell Prolif. 2025 Aug 24;59(5):e70120. doi: 10.1111/cpr.70120 (PMC13114768; doi:10.1111/cpr.70120)
Supplement: Supplementary file 7 — APPENDIX S2: Supporting information. [file CPR-59-e70120-s002.docx]

**Supplementary Table 1. sequence of PCR primers used**.

|  |  | **Primer sequence(5’-3’)** |
| --- | --- | --- |
| **Thra-flox** | **Thra-5wt-tF** | **TACACACTCACACCACCAGAAGGC** |
|  | **Thra-5wt-tR** | **ACCACGCCAGAATCAGCCAGGAAA** |
| **Myf5-cre-ki** | **Forward** | **ATAACGCCCAGCTACAGGGACCAAC** |
|  | **Reverse** | **TGCACACAGACAGGAGCATCTTCC** |
| **Myf5-cre-ki/wt** | **Forward** | **ATAACGCCCAGCTACAGGGACCAAC** |
|  | **Reverse** | **TGGTTGACCTTCTTCAGGCGTCTA** |

**Supplementary Table 2. sequence of RT-qPCR primers used**.

| **mRNAs** |  | **Primer sequence(5’-3’)** |
| --- | --- | --- |
| **TRα** | **Forward** | **GGTCACCAGATGGAAAGCGAA** |
|  | **Reverse** | **CCTTGTCCCCACACACGA** |
| **p16** | **Forward** | **GCTCAACTACGGTGCAGATTC** |
|  | **Reverse** | **GCACGATGTCTTGATGTCCC** |
| **p21** | **Forward** | **CGAGAACGGTGGAACTTTGAC** |
|  | **Reverse** | **CCAGGGCTCAGGTAGACCTT** |
| **p53** | **Forward** | **TTCATTGGGACCATCCTGGC** |
|  | **Reverse** | **GGCAGTCATCCAGTCTTCGG** |
| **BAX** | **Forward** | **TGAAGACAGGGGCCTTTTTG** |
|  | **Reverse** | **AATTCGCCGGAGACACTCG** |
| **BCL-2** | **Forward** | **GTCGCTACCGTCGTGACTTC** |
|  | **Reverse** | **CAGACATGCACCTACCCAGC** |
| **caspase3** | **Forward** | **ATGGAGAACAACAAAACCTCAGT** |
|  | **Reverse** | **TTGCTCCCATGTATGGTCTTTAC** |
| **IP3R1** | **Forward** | **CGTTTTGAGTTTGAAGGCGTTT** |
|  | **Reverse** | **CATCTTGCGCCAATTCCCG** |
| **Grp75** | **Forward** | **ATGGCTGGAATGGCCTTAGC** |
|  | **Reverse** | **ACCCAAATCAATACCAACCACTG** |
| **VDAC1** | **Forward** | **CCCACATACGCCGATCTTGG** |
|  | **Reverse** | **GTGGTTTCCGTGTTGGCAGA** |
| **GAPDH** | **Forward** | **AGGTCGGTGTGAACGGATTTG** |
|  | **Reverse** | **TGTAGACCATGTAGTTGAGGTCA** |

**Supplementary Table 3. primary antibody information used**.

| **antibody** | **information** |
| --- | --- |
| **TRα** | **1:2000, 66703-1-Ig, Proteintech** |
| **p16** | **1:2000, 10883-1-AP, Proteintech** |
| **p21** | **1:2000, 10355-1-AP, Proteintech** |
| **P53** | **1:5000, 80077-1-RR, Proteintech** |
| **IP3R1** | **1:2000, 19962-1-AP, Proteintech** |
| **Grp75** | **1:5000, 14887-1-AP, Proteintech** |
| **VDAC1** | **1:2000, 55259-1-AP, Proteintech** |
| **BAX** | **1:2000, 50599-2-Ig, Proteintech** |
| **Bcl-2** | **1:2000, 26593-1-AP, Proteintech** |
| **cleaved-caspase3** | **1:2000, #9664, CST** |
| **TOM20** | **1:5000, 11802-1-AP, Proteintech** |
| **CytC** | **1:5000, 66264-1-Ig, Proteintech** |
| **GAPDH** | **1:5000, 10494-1-AP, Proteintech** |
| **PI3K** | **1:2000, #4249, CST** |
| **p-Akt** | **1:2000, #4060, CST** |
| **Akt** | **1:2000, #9272, CST** |

**Supplementary Table 4. sequence of ChIP-qPCR primers used**.

|  |  | **Primer sequence(5’-3’)** |
| --- | --- | --- |
| **IP3R1** | **Forward** | **GGCCCAAATGCTCAGAAAGACATGC** |
|  | **Reverse** | **TTTCATCCTCCCCACACTGTAGACAC** |
